# Elevated MANF expression in β cells protects mice from streptozotocin-induced diabetes by attenuating islet stress and immunogenicity

**DOI:** 10.1016/j.ymthe.2026.02.043

**Published:** 2026-02-27

**Authors:** Huini Li, Tatiana Danilova, Erik Palm, Emmi Pakarinen, Julia Kiva, Tõnis Org, Tapani K. Koppinen, Elina Hakonen, Indrek Teino, Merja H. Voutilainen, Timo Otonkoski, Maria Lindahl

**Affiliations:** 1 Institute of Biotechnology, HiLIFE, University of Helsinki, 00014 Helsinki, Finland; 2 Institute of Molecular and Cell Biology, University of Tartu, 51010 Tartu, Estonia; 3 Institute of Genomics, University of Tartu, 51010 Tartu, Estonia; 4 Division of Pharmacology and Pharmacotherapy, Faculty of Pharmacy, University of Helsinki, 00014 Helsinki, Finland; 5 Stem Cells and Metabolism Research Program, Faculty of Medicine, University of Helsinki, 00014 Helsinki, Finland; 6 Children’s Hospital, University of Helsinki and Helsinki University Hospital, 00290 Helsinki, Finland

**Keywords:** diabetes mellitus, β cell, MANF, streptozotocin, endoplasmic reticulum stress, unfolded protein response, immunogenicity, preclinical mouse model, β cell protection and regeneration

## Abstract

Type 1 diabetes (T1D) arises from autoimmune-mediated destruction of insulin-producing β cells, driven in part by endoplasmic reticulum (ER) stress and chronic unfolded protein response (UPR). We previously found that mesencephalic astrocyte-derived neurotrophic factor (MANF), an ER stress-regulating protein with protective and immunomodulatory roles, is essential for mouse and human β cell survival and proliferation. To assess the therapeutic potential of elevated endogenous MANF, we generated β cell-specific transgenic MANF-overexpressing mice and induced diabetes using multiple low-dose streptozotocin (MLDS) injections. In this study, we demonstrate that elevated MANF levels protected against MLDS-induced hyperglycemia, preserved β cell mass, enhanced proliferation, and reduced β cell DNA damage responses and islet lymphocyte infiltration. Transcriptomic profiling of MANF-overexpressing islets revealed downregulation of genes linked to ER and oxidative stress, inflammation, immune responses, antigen presentation, and p53-mediated senescence. Immunophenotyping further showed a reduction in CD4+ T cells in pancreatic lymph nodes. Mechanistically, elevated MANF suppressed MLDS-induced terminal UPR markers, including DNA damage inducible transcript 3 (*Ddit3*) and thioredoxin-interacting protein (TXNIP) expression, whereas MANF deficiency elevated their expression in β cells. Collectively, these findings identify MANF as a dual-acting therapeutic target that alleviates β cell stress and reduces immunogenicity in T1D.

## Graphical abstract

**Figure:**
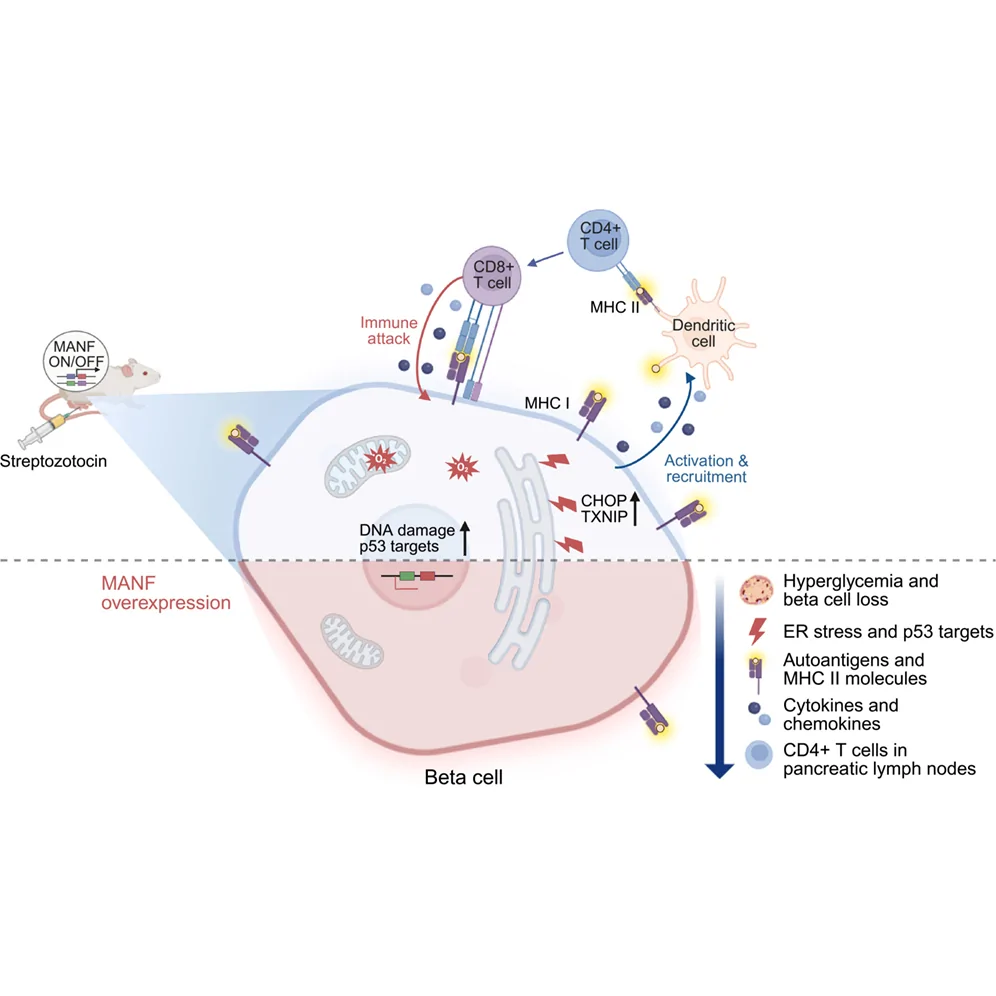

## Teaser

Lindahl and colleagues demonstrate that elevating MANF in pancreatic β cells protects transgenic mice from streptozotocin-induced insulitis and diabetes. Islet transcriptomics and immunophenotyping show that MANF overexpression modulates β cell stress, senescence, and pro-inflammatory signaling, lowering β cell immunogenicity thereby revealing mechanisms by which stress reduction preserves β cells in diabetes.

## Introduction

Type 1 diabetes (T1D) is an autoimmune disorder characterized by progressive destruction of pancreatic β cells due to islet infiltration by immune cells (insulitis), leading to absolute insulin deficiency. External insults such as viral infections, cytokines, and genetic predisposition contribute to chronic β cell endoplasmic reticulum (ER) stress, dysfunctional unfolded protein response (UPR), and eventual β cell death.^1^^,^^2^ Emerging evidence suggests that β cells are not just passive participants during T1D pathogenesis. Their intrinsic vulnerability and high demand of insulin production and folding make them more susceptible to ER stress, provoking the autoimmune attack.^3^^,^^4^ Therefore, novel therapeutic strategies emphasize the importance of reducing β cell stress in T1D in combination with immunotherapy.

Disruptions in ER homeostasis in β cells activate UPR ER transmembrane receptors, conserved inositol-requiring protein 1α (IRE1α), protein kinase RNA (PKR)-like ER kinase (PERK), and activating transcription factor 6 (ATF6).^5^ Initially, activation of these receptors promotes an adaptive UPR to restore protein-folding homeostasis.^6^ However, under irremediable ER stress, the UPR shifts to a chronic terminal UPR, leading to cell death.^7^ Hyperactivated IRE1α engages adapter proteins, triggering apoptosis through the activation of pro-inflammatory genes in pancreatic β cells.^8^ Furthermore, regulated IRE1α-dependent decay increases the stability of mRNA encoding thioredoxin-interacting protein (TXNIP), reducing the antioxidant response and activating the nucleotide-binding oligomerization domain-like receptor family pyrin domain-containing 3 (NLRP3) inflammasome and interleukin-1β (IL-1β) secretion.^9^ Hyperactivated PERK phosphorylates eukaryotic translation initiation factor 2 subunit α (eIF2α) leading to translation arrest, sustained ATF4 translation, and increased expression of pro-apoptotic C/EBP homologous protein (CHOP), TXNIP, and nuclear factor kappa B (NF-κB), contributing to the autoimmune response and β cell death in T1D.^5^^,^^10^ Sustained ER stress has been implicated to modulate the immunopeptidome and immunogenicity of β cells.^2^^,^^3^^,^^11^^,^^12^^,^^13^^,^^14^ In contrast, mild ER stress increases β cell proliferation by reducing tribbles pseudokinase 3 (TRIB3), a negative regulator of protein kinase B (AKT).^15^ Therefore, means of reducing ER stress leading to endogenous β cell protection and regeneration are currently under intensive investigation as therapeutic approaches for diabetes.^3^

Mesencephalic astrocyte-derived neurotrophic factor (MANF) is a small 18 kDa ER-localized protein, highly expressed in metabolically active tissues, that can be secreted from cells in stress.^16^^,^^17^^,^^18^^,^^19^ Recombinant MANF protein protects and restores damaged cells in various animal models of neurodegenerative, inflammatory, and metabolic diseases.^20^ Although its mechanism of action is still unclear, it binds to lipid sulfatides in cell membranes and is suggested to be endocytosed and transported back to the ER.^21^ Additionally, it binds the neuroplastin receptor, alleviates NF-κB-regulated inflammatory responses and promotes insulin secretion in β cells.^22^ MANF engages UPR regulating ER chaperone protein BiP (GRP78/BiP) to increase protein folding.^23^^,^^24^ It also binds all three main UPR receptors and reduces IRE1α activation, oligomerization, and downstream signaling.^25^ Thus, the quantity of MANF may be critical for regulating cellular ER stress levels.

We have previously shown that MANF is needed for the postnatal survival and expansion of pancreatic β cells in mice, as loss of MANF resulted in diabetes due to reduced β cell proliferation and increased β cell death caused by chronic ER stress.^18^^,^^26^ Importantly, loss-of-function mutations in the *MANF* gene in humans cause childhood-onset syndromic diabetes due to increased β cell ER stress, and impaired insulin production and secretion.^27^ Recombinant MANF protein induced proliferation of human and mouse β cells and protected against chemical- and pro-inflammatory cytokine-induced ER stress and cell death *in vitro*.^17^^,^^18^^,^^26^^,^^28^ In addition, exogenous MANF reduced NF-κB activation and its target genes in cytokine-treated human primary β cells.^17^ In our gene therapy experiment, increased MANF in the pancreas, delivered by adeno-associated virus vector, was found to partially protect β cells in a multiple low-dose streptozotocin (MLDS) model. However, due to low transduction efficiency, no therapeutic effect was detected.^18^ For this study, we developed an inducible bi-transgenic mouse model (INS-MANF) overexpressing MANF specifically in β cells. Streptozotocin (STZ) is a toxic glucose analog that enters β cells through glucose transporter 2 (GLUT2), causing DNA damage, oxidative stress, ER stress, and β cell death.^29^^,^^30^ It is a well-established model for studying β cell-intrinsic stress responses that precede autoimmunity in T1D and for evaluating preclinical therapeutic strategies in rodents.^30^ We induced diabetes in INS-MANF and control mice using both the single high-dose STZ (SHDS) model, causing acute β cell cytotoxicity and death,^31^ and the milder MLDS model mimicking immune-mediated T1D through progressive β cell stress and subsequent islet immune cell infiltration.^32^

Together, our results show that elevated MANF efficiently protects against MLDS-induced β cell death, insulitis, and hyperglycemia. RNA sequencing (RNA-seq) of islets from prediabetic mice revealed that the protective effect of MANF was associated with reduced ER and oxidative stress, DNA double-strand break (DSB) repair, p53-mediated senescence, and immune cell recruitment and activation. Importantly, these changes correlated with enhanced β cell survival and regeneration, suggesting that elevating MANF levels in β cells simultaneously resolves β cell stress and reduces immunogenicity in T1D.

## Results

### Elevated MANF does not affect β cell mass or β cell function

**Figure 1:**
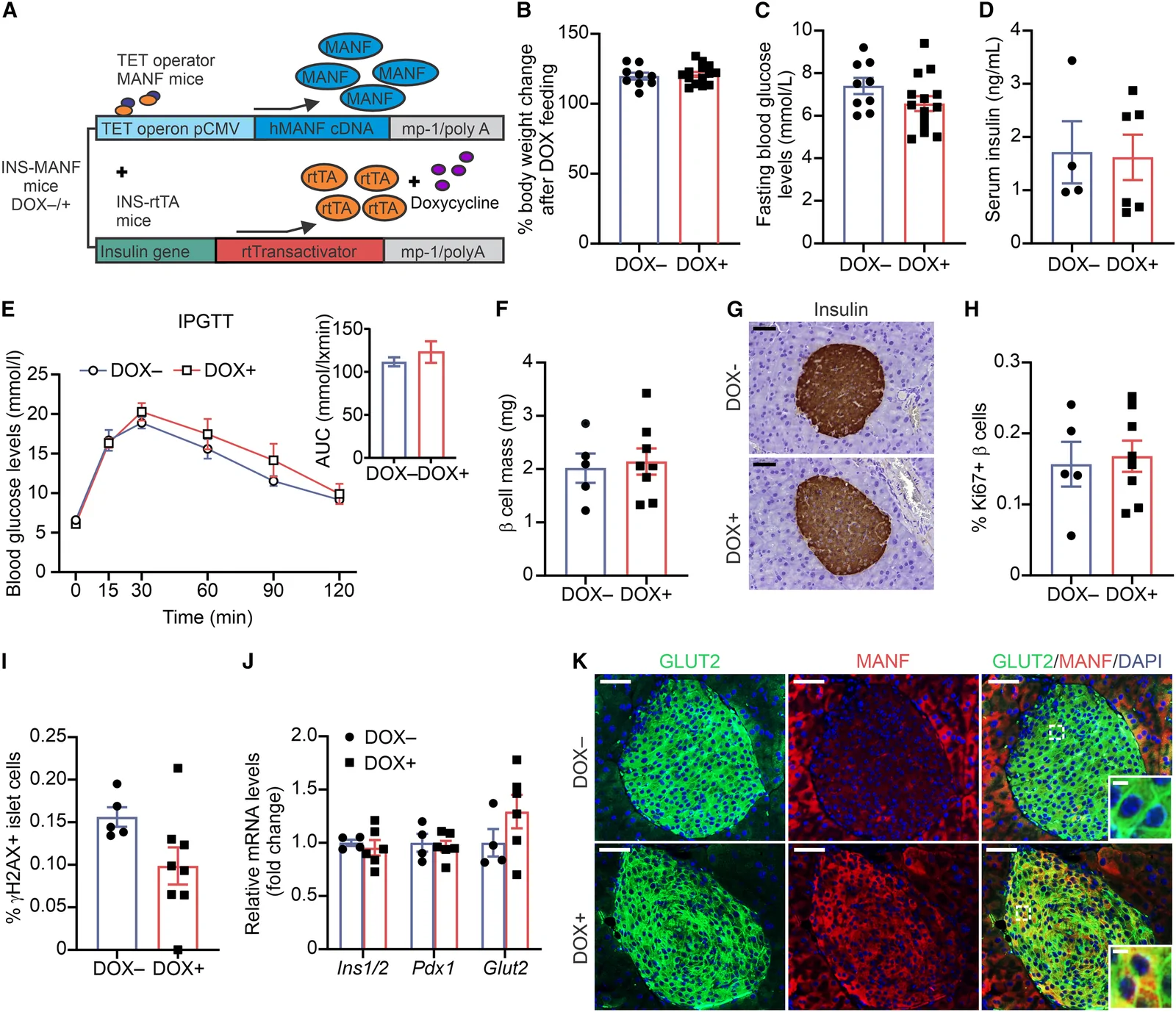
Characterization of male bi-transgenic INS-MANF mice on doxycycline chow for 3 months (A) Schematic illustration of the transgenic constructs for INS-rtTA and TET-MANF single-transgenic mice used for generating bi-transgenic INS-MANF mice. (B) Body weight gain (%) in MANF-overexpressing (INS-MANF DOX+, *n* = 14) compared with INS-MANF DOX– (*n* = 9) mice 3 months after DOX+ or normal chow feeding. (C) Blood glucose levels from fasted mice (DOX+, *n* = 14; DOX–, *n* = 9). (D) *Ad libitum* serum insulin levels (DOX+, *n* = 6; DOX–, *n* = 4), and (E) intraperitoneal glucose tolerance test (IPGTT) and area under the curve (AUC) of the mean blood glucose levels for each group (DOX+, *n* = 14; DOX–, *n* = 9). (F and G) β cell mass quantified from insulin antibody-stained pancreas sections of INS-MANF DOX+ (*n* = 8) and DOX– (*n* = 5) mice. Scale bars, 50 μm (G). (H) Relative number of proliferating β cells quantified from pancreas sections stained with Ki67 and insulin antibodies (DOX+, *n* = 8; DOX–, *n* = 5). (I) Relative number of γH2AX-positive cells in islets (DOX+, *n* = 8; DOX–, *n* = 5). (J) RT-qPCR analysis of β cell identity genes in islets (DOX+, *n* = 6; DOX–, *n* = 4). (K) Membrane localization of GLUT2 in pancreas sections stained with anti-MANF (red) and anti-GLUT2 (green) antibodies. Nuclei were stained by DAPI (blue). Scale bars, 50 and 5 μm (insets). Data are expressed as mean ± SEM, and scatterplots show all individual data points. Statistical analyses were performed using Student’s two-tailed unpaired *t* test for (B–D), AUC in (E), (F), and (H–J) and two-way ANOVA followed by Bonferroni’s post hoc test for the different time points in (E).

To test the therapeutic effect of local overexpression of human MANF in β cells of STZ-treated diabetic mice, we generated a MANF transgenic mouse line (TET-MANF) utilizing the TET-On system. By crossing this line with an insulin promoter-dependent reverse tetracycline transactivator (INS-rtTA)-expressing mouse line, human MANF expression can be specifically induced in β cells by doxycycline (DOX) administration (Figure 1A). First, the bi-transgenic mice were examined in more detail. We confirmed a significant and specific overexpression of the human transgenic MANF in insulin-positive β cells of bi-transgenic INS-MANF mice on a DOX+ diet compared with INS-MANF DOX– control mice (Figures S1A–S1G). Immunostaining of pancreas sections confirmed that transgenic MANF expression was specific to β cells but not in α cells (Figures S1G and S1H). A small increase in MANF expression was seen in islets from a few bi-transgenic INS-MANF DOX– mice (not shown), indicating minor leakiness of the TET-On system, as previously reported.^33^ Continuous MANF overexpression in β cells for 3 months did not lead to detectable systemic or local islet adverse effects, including body weight change, glycemic status, serum insulin levels, β cell function, β cell mass, proliferation or survival, or the expression of β cell-specific genes including *Glut2* (Figures 1B–1K). In some transgenic lines utilizing the insulin 2 promoter, ectopic transgene expression has been found induced in nutrient-sensing neurons of the hypothalamus.^34^ However, we confirmed no change in transgenic human MANF expression in the hypothalami of INS-MANF DOX+ mice (Figures S1I and S1J). We further tested the long-term effects of β cell MANF overexpression for 5 months (Figures S2A–S2F) and 11 months (Figure S2G) in male mice and found no β cell hypertrophy or islet morphological or pathological changes *in vivo*.

### Transgenic MANF protects against single high-dose STZ-induced diabetes

**Figure 2:**
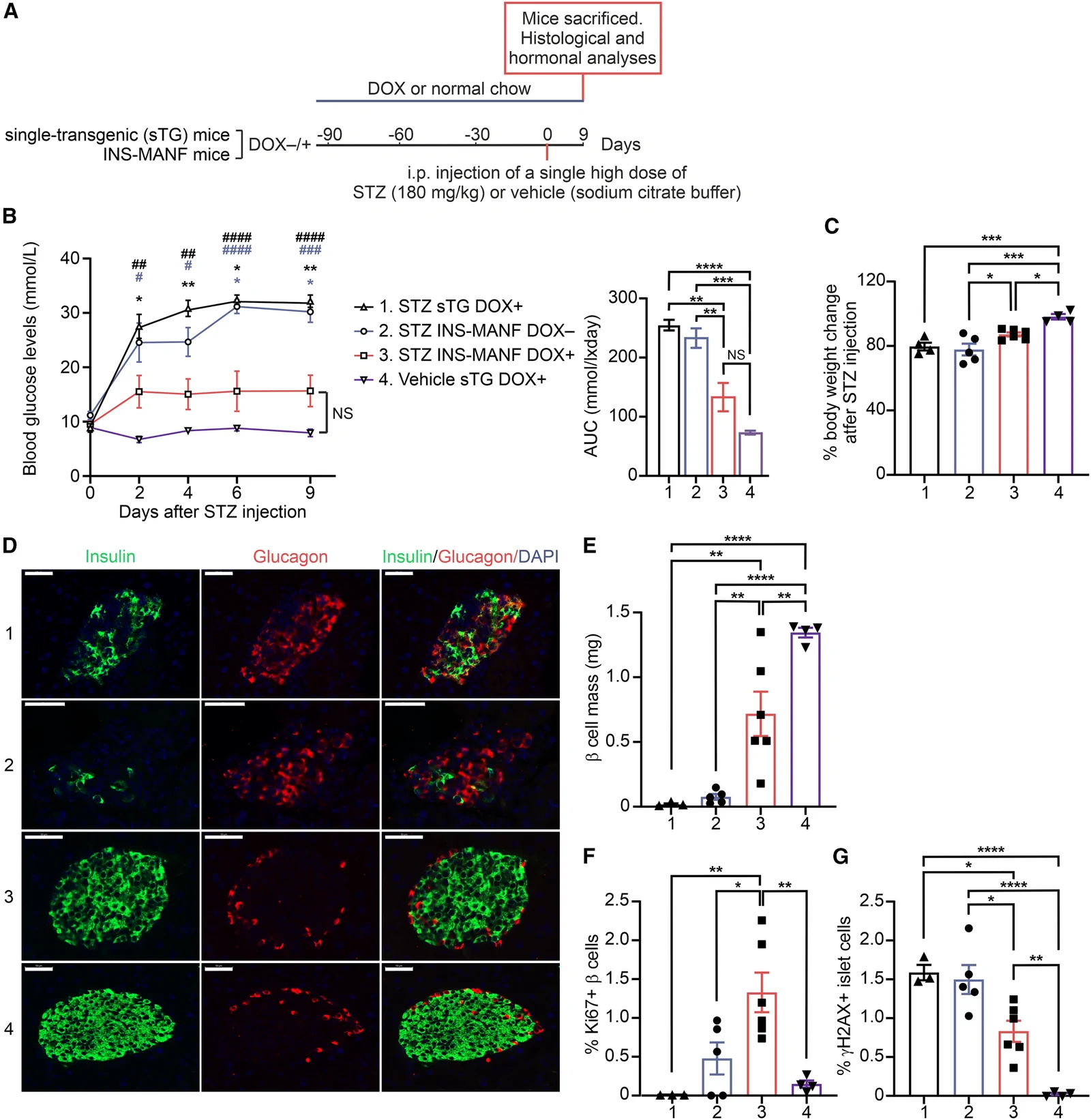
Elevated MANF expression in β cells protects from a single high dose of streptozotocin-induced diabetes (A) Timeline for DOX or normal chow feeding, followed by a single high dose of STZ injection (180 mg/kg). Non-diabetic controls (INS-rtTA DOX+) received equivalent 25 mM sodium citrate buffer (pH 4.5). (B) *Ad libitum* blood glucose levels measured before STZ injection and at four time points up to 9 days after injection and area under the curve (AUC) of the mean blood glucose levels for each group: (1) STZ TET-MANF sTG DOX+ (*n* = 4), triangles, black line; (2) STZ INS-MANF DOX– (*n* = 5), circles, blue line; (3) STZ INS-MANF DOX+ (*n* = 6), squares, red line; (4) vehicle INS-rtTA sTG DOX+ (*n* = 4), triangles, purple line. Statistical significance is indicated as follows: black ∗ for group 3 vs. group 1; blue ∗ for group 3 vs. group 2; black # for group 4 vs. group 1; and blue # for group 4 vs. group 2. No significant difference (NS) was observed between group 3 and group 4. (C) Body weight change (%) 9 days after STZ or vehicle injection. (D) Anti-insulin and anti-glucagon antibody-stained pancreatic sections from STZ-treated and control mice. Scale bars, 50 μm. (E) β cell mass calculated from insulin-stained pancreas sections. (F) β cell proliferation calculated from Ki67- and insulin-stained pancreas sections. (G) Number of γH2AX-positive cells compared with total islets cells. Data are expressed as mean ± SEM, and scatterplots show all individual data points. Statistical analyses were performed using two-way ANOVA followed by Tukey's post hoc test for the different time points in (B), and one-way ANOVA followed by Tukey's post hoc test for AUC in (B), (C), and (E–G). ∗*p* < 0.05, ∗∗*p* < 0.01, ∗∗∗*p* < 0.001, ∗∗∗∗*p* < 0.0001, #*p* < 0.05, ##*p* < 0.01, ###*p* < 0.001, ####*p* < 0.0001, NS > 0.05.

We then tested whether increased transgenic MANF in β cells protects against SHDS-induced diabetes. Male INS-MANF and control single-transgenic (sTG) mice were fed with a DOX+ diet for 3 months, whereas control INS-MANF mice received normal food (DOX–) prior to SHDS injection (Figure 2A). SHDS resulted in loss of β cells and hyperglycemia in both control single- and bi-transgenic STZ-injected mice within 2 days (Figure 2B). In contrast, MANF-overexpressing mice (INS-MANF DOX+) showed significantly lower blood glucose levels throughout the experiment, which were not significantly higher than the control sTG DOX+ mice not injected with STZ (Figure 2B). Due to hyperglycemia, control STZ-injected mice lost a significant amount of weight (20%–22%) and all mice were sacrificed at day 9 after the STZ injection (Figure 2C). As expected, the weight loss of MANF-overexpressing mice was significantly smaller (13%) compared with the control STZ mice. Immunohistochemical analysis of pancreas sections revealed conserved islet architecture in the MANF-overexpressing mice compared with STZ-treated control mice (Figure 2D). Quantification of the β cell mass revealed preserved β cell mass in the STZ-treated MANF-overexpressing pancreases compared with the STZ-treated control mice (Figure 2E). Interestingly, increased β cell proliferation was found in islets from MANF-overexpressing STZ-treated mice compared with control mice (Figure 2F). DNA damage response (DDR) is activated in β cells of T1D patients.^35^ It is also induced by STZ in mice, which leads to DNA DSBs, islet inflammation, and β cell death.^35^ Phosphorylation of histone variant H2AX at serine 139 (γH2AX) is a widely used marker for DSBs, DDR, senescence, and apoptotic DNA fragmentation.^36^ We found less-frequent γH2AX-positive islet cells in STZ-treated MANF-overexpressing pancreases compared with controls (Figure 2G). Importantly, DOX feeding alone did not prevent the STZ-induced β cell death and diabetes in control sTG DOX+ mice (Figures 2B–2G).

### Elevated MANF in β cells protects against multiple low doses of STZ-induced diabetes

**Figure 3:**
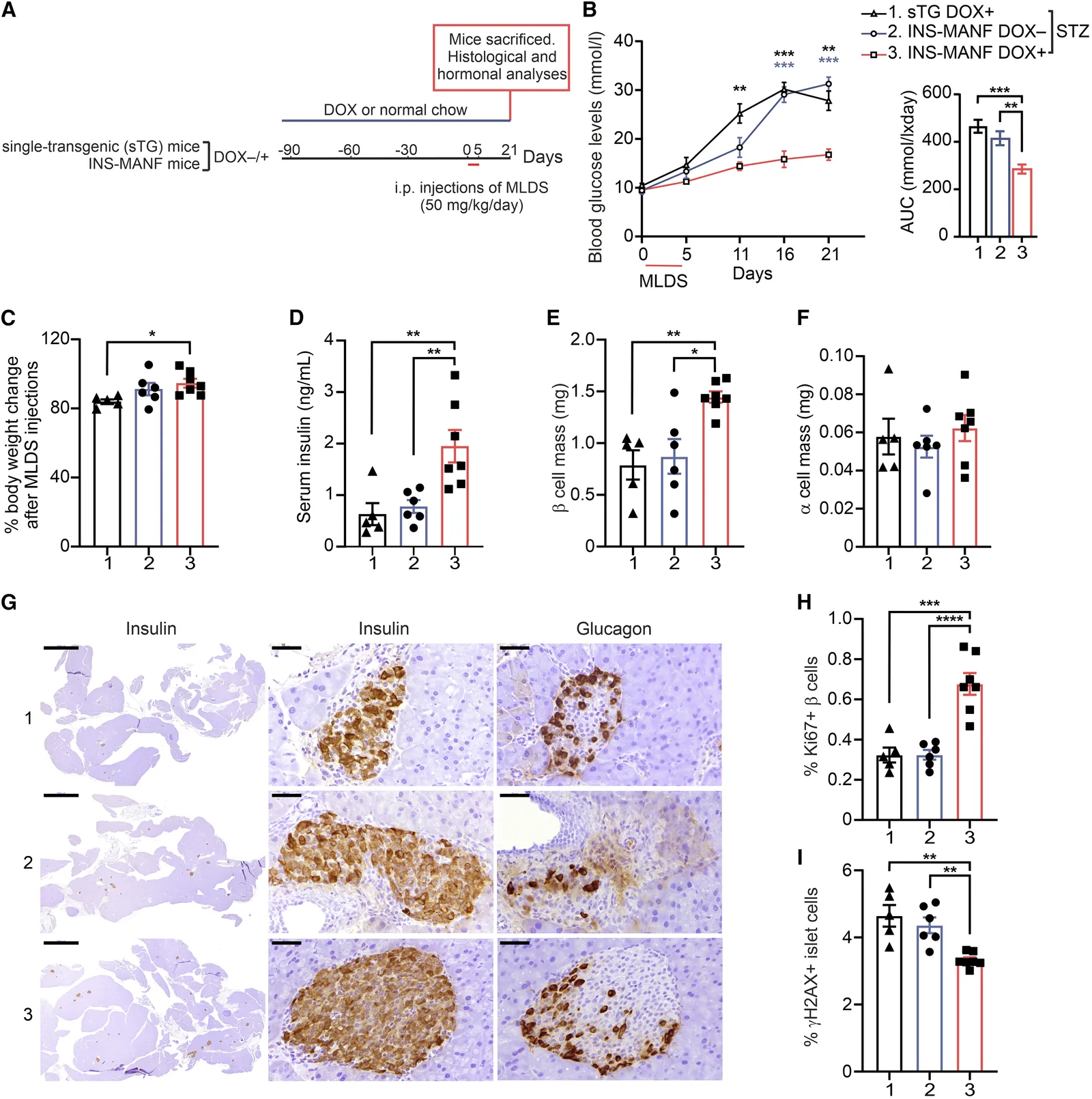
Elevated β cell-specific MANF expression protects from multiple low doses of STZ-induced hyperglycemia, β cell loss, and induces β cell regeneration (A) Timeline for DOX+ and normal diet feeding, followed by MLDS injections (50 mg/kg/day for 5 consecutive days). (B) Measurements of blood glucose levels of *ad libitum* fed animals and area under the curve (AUC) of the mean blood glucose levels for each group: (1) STZ sTG DOX+ mice including TET-MANF DOX+ (*n* = 3) and INS-rtTA DOX+ mice (*n* = 2), triangles, black line; (2) STZ INS-MANF DOX– mice (*n* = 6), circles, blue line; and (3) STZ INS-MANF DOX+ mice (*n* = 7), squares, red line. Statistical significance is indicated as follows: black ∗ for group 3 vs. group 1; and blue ∗ for group 3 vs. group 2. (C) Body weight change (%) 21 days after the first STZ injection. (D) *Ad libitum* serum insulin levels from terminal blood. (E) β cell mass quantified from insulin-stained pancreas sections of mice sacrificed 21 days after the first STZ injection. (F) Quantification of α cell mass from glucagon-stained sections. (G) Images from insulin and glucagon antibody-stained pancreatic sections of STZ-treated mice. Scale bars, 2,000, 50, and 50 μm (from left to right). (H) β cell proliferation quantified from insulin and Ki67 antibody-stained sections in STZ-treated mice. (I) β cell DDR assessed by anti-γH2AX antibody staining in islets from STZ-injected mice. Data are expressed as mean ± SEM, and scatterplots show all individual data points. Statistical analyses were performed using two-way ANOVA with Tukey’s post hoc test for the different time points in (B) and one-way ANOVA followed by Tukey’s post hoc test for AUC in (B), (C–F), and (H–I). ∗*p* < 0.05, ∗∗*p* < 0.01, ∗∗∗*p* < 0.001, ∗∗∗∗*p* < 0.0001.

Next, we studied whether elevated MANF levels in β cells confer protection against MLDS-induced T1D. As previously, INS-MANF mice and control sTG mice were pre-fed with a DOX diet, whereas control INS-MANF mice received normal food (Figure 3A). As expected, the development of hyperglycemia in this model was slower in control mice compared with the SHDS model. Mice were sacrificed when control mice reached severe hyperglycemia 21 days after the first STZ injection (Figure 3B). Blood glucose levels in MANF-overexpressing mice increased marginally but remained low compared with STZ-injected control mice throughout the experiment (Figure 3B). The weight loss of MANF-overexpressing mice was significantly smaller (5.4%) than in the STZ-injected control sTG mice (16%) (Figure 3C). Consequently, endpoint serum insulin levels in fed state animals were significantly higher in MANF-overexpressing mice compared with control mice (Figure 3D). Similarly, the β cell mass was higher in MANF-overexpressing mice compared with control STZ-treated mice (Figure 3E), whereas no difference in α cell mass was detected (Figure 3F). The islet architecture was relatively intact in MANF-overexpressing pancreases (Figure 3G). This result was in line with the increased β cell proliferation (Figure 3H) and reduced β cell DSBs (Figure 3I) in the MANF-overexpressing islets compared with STZ-treated controls, suggesting a protective but also a regenerative capacity for elevated MANF after insult.

A separate set of MANF-overexpressing mice were treated with MLDS and followed for 4.5 months to analyze whether β cell-specific MANF overexpression normalizes blood glucose levels after MLDS injections with time (Figure S3A). Control mice needed to be sacrificed earlier when reaching hyperglycemia (Figure S3B). MANF-overexpressing mice had a transient episode of slightly elevated blood glucose levels but returned to normoglycemia with normal serum insulin levels at 4.5 months after the first STZ injection (Figures S3B and S3C). A relatively higher β cell mass with a trend for increased β cell proliferation and reduced β cell death was detected in the pancreases of MANF-overexpressing mice, indicating sustained β cell protection over time compared with control mice at 45 days after the first STZ injection (Figures S3D–S3F).

### MANF-induced β cell protection is associated with reduced islet insulitis, terminal UPR, and CD4+ T cells in pancreatic lymph nodes

**Figure 4:**
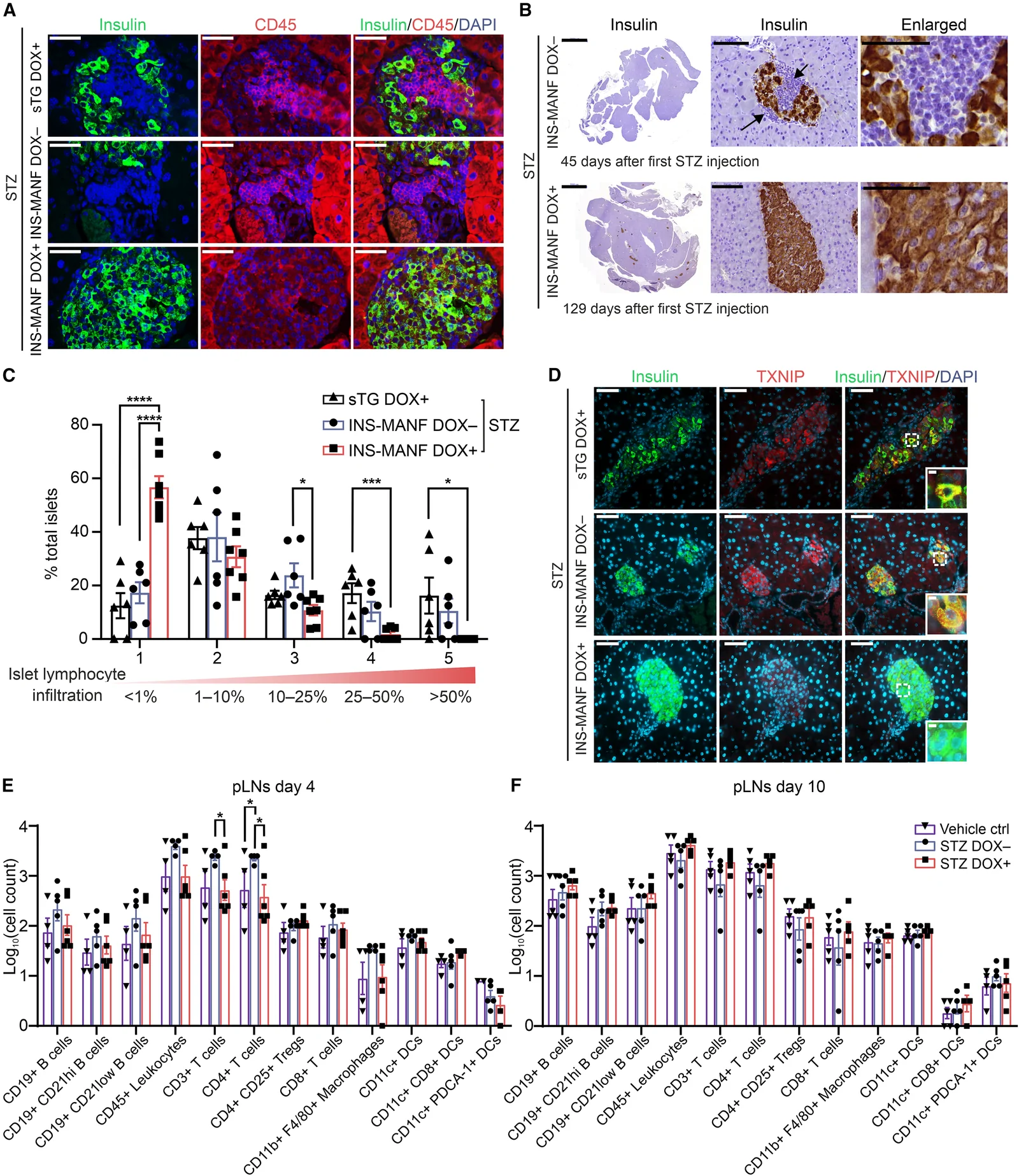
Elevated MANF expression in β cells efficiently protects against islet insulitis, TXNIP upregulation and reduces the number of CD4+ T cells in pancreatic lymph nodes (A) Representative pictures of CD45- and insulin-stained pancreatic sections from MLDS-injected mice 21 days after the first STZ injection. Scale bars, 50 μm. (B) Representative pictures of insulin- and hematoxylin-counterstained pancreas with islet lymphocyte infiltration (arrows) in MLDS-injected DOX– compared with DOX+ islets. Scale bars, 2 mm, 100 μm, and 50 μm (left to right). (C) Islet insulitis 21 days after the first STZ injection was scored and calculated as percent of islets per mouse at each stage of insulitis, from hematoxylin and eosin-stained pancreas sections, using severity scaling 1–5. Treatment groups include STZ-treated sTG DOX+ mice (STZ sTG DOX+, *n* = 5), triangles, black bar; STZ INS-MANF DOX– (*n* = 6), circles, blue bar; and STZ INS-MANF DOX+ (*n* = 7), squares, red bar. (D) Representative pictures of TXNIP (red) co-stained with insulin (green) in pancreatic sections from MLDS-injected INS-MANF and control mice 21 days after first STZ injection. Scale bars, 50 and 10 μm (insets). (E and F) Immune cell profiling in pLNs on day 4 (E) and day 10 (F) after the first STZ (45 mg/kg/day) or vehicle injections. Treatment groups include non-diabetic control mice injected with vehicle (Vehicle ctrl, *n* = 4), triangles, purple bar including TET-MANF DOX– (*n* = 1), INS-rtTA DOX– (*n* = 2), and INS-MANF DOX– (*n* = 1), INS-MANF DOX– mice injected with STZ (STZ DOX–, *n* = 5), circles, blue bar, and INS-MANF DOX+ mice injected with STZ (STZ DOX+, *n* = 6), squares, red bar. Cell counts were log10-transformed prior to analysis. Data are expressed as mean ± SEM, and scatterplots show all individual data points. Statistical analyses were performed using one-way ANOVA with Tukey’s post hoc test for the different scores in (C) and two-way ANOVA followed by Holm-Šidák’s multiple comparison test for (E) and (F). ∗*p* < 0.05, ∗∗∗*p* < 0.001, ∗∗∗∗*p* < 0.0001.

Immunostaining revealed that MLDS-injected control mice developed insulitis with destroyed islet architecture infiltrated by CD45+ lymphocytes and a dramatic loss of pancreatic β cells, whereas MLDS-injected MANF-overexpressing mice had more intact islets and β cells (Figure 4A), similarly to MANF-overexpressing mice maintained for 4.5 months after the first STZ injection (Figure 4B). The severity of insulitis (Figure S3G) was measured 3 weeks after the first STZ injection in the MLDS model from hematoxylin and eosin-stained pancreas sections (Figure 4C). Results from scoring revealed that 57.7% ± 4% of the islets in the MANF-overexpressing mice were intact with no insulitis (score 1, Figure 4C), compared with 9.2% ± 4% of the control sTG mice and 17.3% ± 4% of the INS-MANF DOX– mice. In addition, islets from MANF-overexpressing mice rarely showed major insulitis (score 5, Figure 4C). Insulitis assessment of islets from MANF-overexpressing mice, 4.5 months after the first MLDS injection, confirmed that elevated MANF in β cells effectively protects against lymphocyte islet infiltration in the MLDS model (Figure S3H). TXNIP, a key mediator of oxidative stress and inflammasome activation in β cells, is upregulated in terminal UPR driving both β cell pro-inflammatory signaling and apoptosis.^37^^,^^38^ In contrast, loss of TXNIP in mice leads to increased β cell mass and resistance to STZ-induced diabetes.^39^ Co-staining of pancreatic sections from control mice 21 days after the first STZ injection revealed increased expression of TXNIP and IL-1β in insulin-positive β cells, whereas TXNIP and IL-1β staining were weak in MANF-overexpressing islets (Figures 4D and S4).

To further investigate the early effects of elevated β cell-specific MANF on the immune response in the MLDS model, immunophenotyping by flow cytometry was performed. Immune cell populations in the pancreatic lymph nodes (pLNs) and spleens of vehicle-treated non-diabetic control, STZ-treated MANF-overexpressing, and INS-MANF DOX– mice were quantified at days 4 and 10 after the first MLDS injection according to a previous study.^40^ A panel of 13 antibodies was used to profile most of the immune cell types described to contribute to the immunopathology in T1D (gating strategy, Figure S5).^41^ Islet antigens carried by antigen-presenting cells from inflamed islets to the pLNs are critical contributors to the pathogenesis of T1D by initiating proliferation and priming of autoreactive T cells.^42^ As expected, most immune cell populations showed a trend toward increased number in the pLNs at day 4 after STZ treatment. Notably, the number of CD4+ T helper cells was significantly increased after STZ treatment (Figure 4E). Conversely, β cell-specific MANF overexpression significantly reduced the STZ-induced increase in both CD3+ T cells and CD4+ T helper cells in the pLNs (Figure 4E). The number of B cells, CD8+ T cells, macrophages, and pan-dendritic cells showed a similar trend in the pLNs (Figure 4E). However, on day 10 after the STZ treatment no significant alterations in different immune cells in the pLNs were detected (Figure 4F). In addition, elevated β cell-specific MANF expression did not affect immune cell populations in the spleen, except for an increase in the number of CD11c+ CD8+ dendritic cells at day 4 (Figures S6A and S6B).

### β cell-specific MANF lowers STZ-induced islet expression of immune, oxidative stress, and p53-mediated senescence genes in prediabetic mice

To further investigate the molecular mechanisms of MANF-induced β cell protection against STZ, we performed bulk RNA-seq following poly(A) selection of mRNA on islets isolated from the pancreases of vehicle- and STZ-injected control mice (INS-MANF DOX–, sTG DOX+, and sTG DOX–) and MANF-overexpressing mice (INS-MANF DOX+) on the 4th day after 3 consecutive days of low doses of STZ or vehicle injections. We confirmed that transgenic human *MANF* was expressed in all replicates isolated from each INS-MANF DOX+ mouse by reverse-transcription quantitative PCR (RT-qPCR) (Figure S7A). Using principal-component analysis, we demonstrated that DOX treatment itself did not induce major global changes in gene expression compared with the strong effect of STZ (Figure S7B).

**Figure 5:**
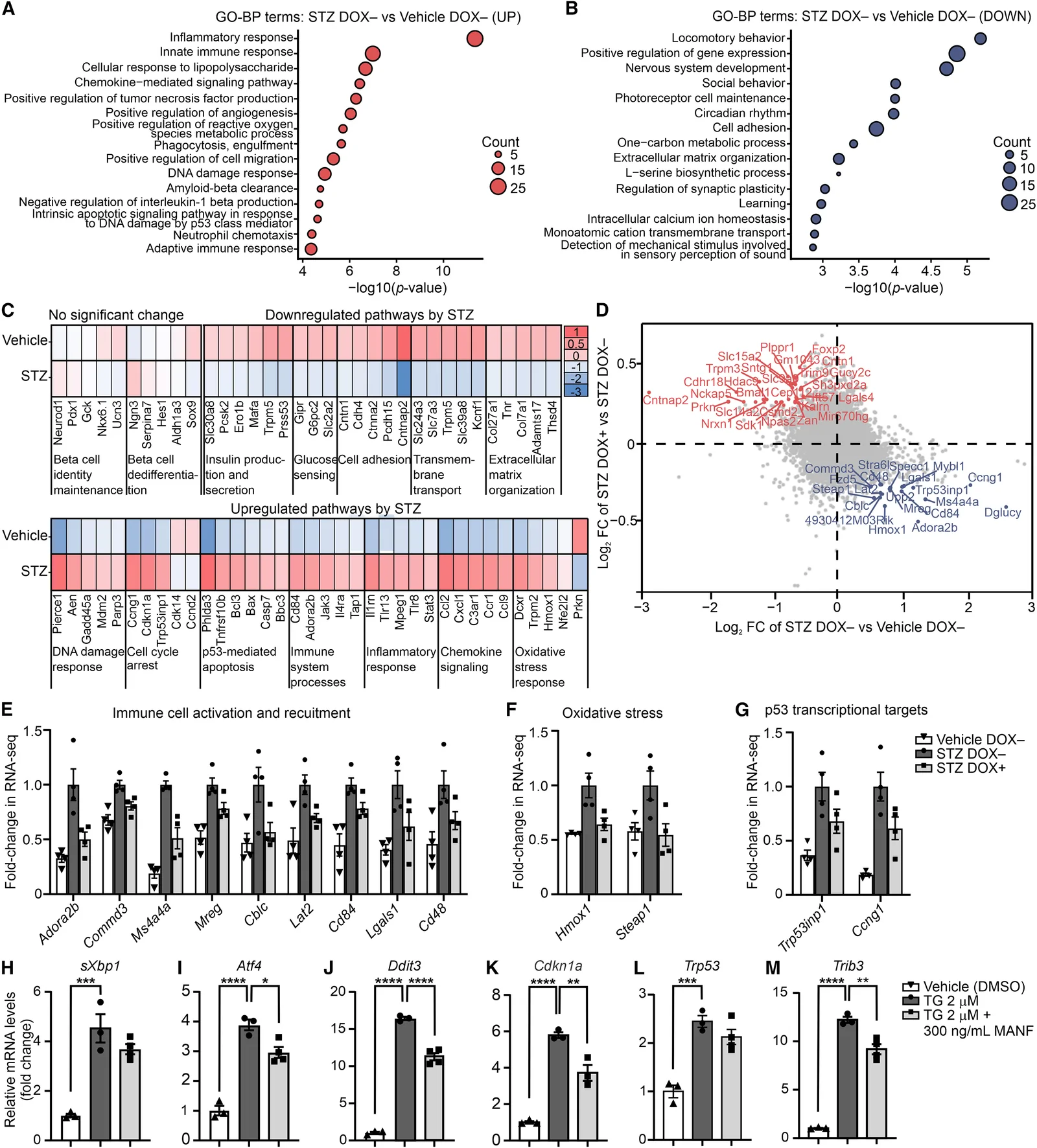
Reduced expression of genes associated with immune cell activation and recruitment, oxidative stress, and p53 transcriptional targets in islets from STZ-injected INS-MANF DOX+ mice (A and B) Bubble diagrams of the GO enrichment analyses for biological processes (BP) terms by DAVID, on islet mRNA using 322 significantly upregulated DEGs (A) and 321 significantly downregulated DEGs (B) with |Log2(FC)| > 0.5 and FDR < 0.05 showing the effect of STZ in INS-MANF DOX– islets (STZ DOX–, *n* = 4) compared with vehicle-treated mice (Vehicle DOX–, *n* = 4). (C) Heatmap and relative average expression of genes and pathways differentially regulated by STZ compared with vehicle treatment in islets from INS-MANF DOX– control mice selected from the 643 DEGs (|Log_2_(FC)| > 0.5, FDR < 0.05, *n* = 4). (D) DEGs in islets between STZ and vehicle-injected INS-MANF DOX– mice (|Log_2_(FC)| > 0.5, FDR < 0.05), plotted against islet mRNA from STZ-injected INS-MANF DOX+ and INS-MANF DOX– mice (STZ DOX+ vs. STZ DOX–, |Log_2_(FC)| > 0.25, *p* < 0.05). Red dots mark the 29 genes downregulated by STZ alone but upregulated by MANF in the STZ DOX+ vs. STZ DOX– group. Blue dots mark the 21 genes that were upregulated by STZ alone but downregulated by MANF in the same groups. (E–G) mRNA expression levels of representative genes from RNA-seq data involved in immune cell activation and recruitment (E), oxidative stress (F), and p53 transcriptional targets (G) among the 21 genes that were upregulated by STZ alone and downregulated by MANF overexpression. (H–M) The relative mRNA expression levels of marker genes for the UPR (H–J), senescence genes *Cdkn1a* (p21) (K) and *Trp53* (p53) (L), and *Trib3* (M) measured by RT-qPCR in isolated WT mouse islets treated with ER stressor thapsigargin (TG) or TG together with rhMANF protein for 16 h. Data are expressed as mean ± SEM, and scatterplots show all individual data points. Statistical analyses were performed using one-way ANOVA with Tukey’s post hoc test for (H–M). ∗*p* < 0.05, ∗∗*p* < 0.01, ∗∗∗*p* < 0.001, ∗∗∗∗*p* < 0.0001.

The transcriptional effect of STZ alone was first compared between islets from vehicle- and STZ-treated INS-MANF DOX– control mice. We identified 643 differentially expressed genes (DEGs) with Log2 fold change (|Log_2_FC|) > 0.5 and FDR < 0.05 (Table S1), including 322 upregulated and 321 downregulated genes in response to STZ. Gene Ontology (GO) enrichment analyses of 322 upregulated DEGs by STZ using the DAVID functional annotation tool showed that the top enriched terms describing biological processes (BPs) were involved in inflammatory and innate immune responses, chemokine-mediated signaling, tumor necrosis factor production, ROS metabolic processes, DDRs, and intrinsic apoptotic signaling in response to DNA damage by the p53 class mediator (Figure 5A; Table S1). Among the 321 downregulated genes by STZ, the top BP terms involved locomotor behavior, positive regulation of gene expression, cell adhesion, extracellular matrix organization, intracellular calcium ion homeostasis, and monoatomic cation transmembrane transport (Figure 5B; Table S1). Results on GO-BP terms and KEGG enrichment pathway analyses are shown in more detail in Figures S7C–S7F and Table S1. A heatmap was generated to highlight the relative average expression levels of key islet genes affected by STZ alone, selected from the 643 DEGs (Figure 5C). STZ treatment significantly reduced the expression of genes involved in insulin production, secretion, and glucose sensing, indicating early β cell dysfunction. Meanwhile, genes essential for β cell identity and progenitor markers remained unchanged (Figure 5C). Thus, the time point set for RNA-seq in our prediabetic MLDS model detects stressed β cells without complete loss of β cell identity, but alongside immune responses associated with markers for senescence and apoptosis (supplemental information).^40^^,^^43^^,^^44^

When comparing the DEGs altered by STZ alone to genes affected by MANF overexpression (|Log_2_FC| > 0.25, *p* < 0.05), we found 21 genes increased by STZ but decreased by MANF overexpression in the presence of STZ (Figure 5D; Table S1). The genes upregulated by STZ but downregulated by MANF were mostly related to the regulation of immune cell activation and recruitment, cytokine and chemokine production, as well as immune cell adhesion and migration, which are essential processes modulating immune cell interaction with β cells (Figures 5D and 5E). This aligns with our observations of reduced islet infiltration of immune cells and decreased numbers of CD4+ T cells in pLNs of MLDS-injected MANF-overexpressing mice (Figure 4E). In addition, reduced expression of *Hmox1* and *Steap1*, two oxidative response genes, was found in the MANF-overexpressing islets, suggesting a lowered oxidative burden and diminished activation of cellular antioxidant defenses (Figures 5D and 5F). The expression of *Ccng1*, encoding cyclin G1, and *Trp53inp1*, encoding tumor protein 53-inducible nuclear protein 1, both key p53 transcriptional targets involved in G1 cell-cycle arrest and senescence in response to cellular stress, was also downregulated by elevated MANF expression (Figures 5D and 5G). To further investigate whether MANF could reduce targets for cell-cycle arrest and senescence by alleviating ER stress, we treated primary mouse islets with ER stressor thapsigargin (TG), an inhibitor of the sarco/endoplasmic reticulum Ca^2+^-ATPase (SERCA) pump, alone or in combination with recombinant human MANF (rhMANF) protein. In addition to reducing UPR markers (Figures 5H–5J), MANF also attenuated TG-induced expression of the senescence marker *Cdkn1a* (p21) and showed a trend toward reduced *Trp53* (p53) expression (Figure 5K and 5L) as well as *Trib3*, an AKT inhibitor (Figure 5M).

RNA-seq revealed that many of the genes downregulated by STZ but upregulated by MANF were involved in cell adhesion (*Cntnap2*, *Sdk1*, *Cdhr18*, *Nrxn1*, *Cntn1)*, synaptic function and neurotransmission (*Trim9*, *Sntg1*, *Cntn1*, *Nrxn1*), neuronal plasticity (*Foxp2*, *Npas2*, *Kalrn*, *Cntnap2*, *Sh3pxd2a*), Ca^2+^ signaling (*Trpm3*, *Gucy2c*, *Slc9a9*, *Slc15a2*), and transcriptional regulation and gene expression (*Foxp2*, *Hdac9*, *Mir670hg*) (Figure 5D). The increased expression of neuronal genes is not surprising as the exocytic system involving pulsatile hormonal secretion in islets is very similar to those of neuronal signaling.^45^ MANF, as a neurotrophic factor, may also maintain neuronal innervation and survival, and thereby preserve islet endocrine function.^46^

### Elevated β cell-specific MANF reduces STZ-induced UPR, antigen presentation, and autoantigen genes

**Figure 6:**
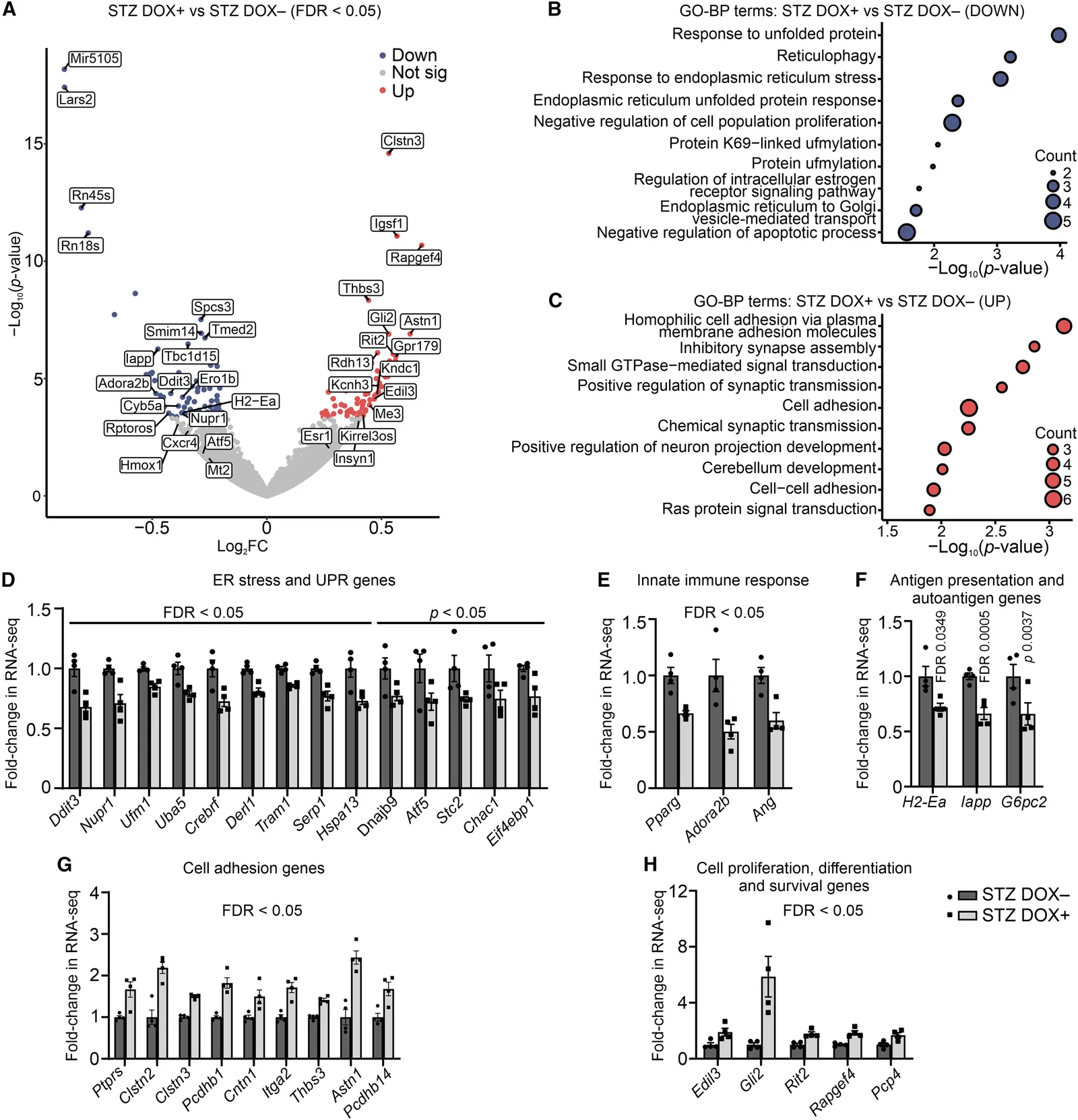
Islets from MANF-overexpressing mice have altered gene expression associated with the UPR, autoantigens, antigen-presenting MHC class II molecule, cell adhesion, and proliferation upon STZ (A) Volcano plot of the differentially expressed genes in islets analyzed with DESeq2 between STZ (45 mg/kg/day)-treated INS-MANF DOX +/– mice (STZ DOX+ vs. STZ DOX–, *n* = 4 per group). Significantly downregulated DEGs (FDR < 0.05) by MANF overexpression are labeled in blue, while upregulated DEGs are labeled in red. Genes of interest are shown. (B and C) Bubble diagrams of GO enrichment analyses in terms of BP using DAVID tool for the 64 genes downregulated (B), FDR < 0.05, and 75 genes upregulated (C), FDR < 0.05, by MANF overexpression compared with the STZ DOX– group ranked according to *p* values. (D–H) mRNA expression levels of representative genes from RNA-seq data involved in ER stress and UPR (D), innate immune response (E), antigen presentation and autoantigens (F), cell adhesion (G), and cell proliferation, differentiation, and survival (H). Data are expressed as mean ± SEM, and scatterplots show all individual data points. FDR and *p* values shown were obtained from differential expression analysis using DESeq2 on RNA-seq data.

To study the effect of MANF overexpression upon STZ treatment, we compared DEGs solely between STZ-injected INS-MANF DOX+ and DOX– mice. DEG analyses with DESeq2 identified 64 significantly downregulated genes and 75 upregulated genes by MANF (FDR < 0.05) shown in a volcano plot (Figure 6A; Table S2). Using the DAVID tool, we further performed GO enrichment analyses separately for the downregulated and upregulated genes affected by elevated MANF between the STZ-injected INS-MANF DOX± groups (FDR < 0.05) (Figures 6B and 6C; Table S2). Interestingly, the top enriched terms describing BP using the downregulated genes were mainly involved in ER stress response, UPR, protein UFMylation, reticulophagy, and ER to Golgi vesicle-mediated transport (Figure 6B). Importantly, the pro-apoptotic UPR genes, *Ddit3* (CHOP) and *Nupr1*, were significantly downregulated by MANF overexpression upon STZ (Figures 6A and 6D). In addition, ER stress-related genes encoding proteins involved in UFMylation (*Ufm1*, *Uba5*), reticulophagy (*Crebrf*, *Hspa13)*, ER-associated degradation (*Derl1*, *Serp1*), and ER translocation (*Tram1*) were significantly downregulated by MANF with FDR < 0.05 (Figure 6D). Additional genes associated with ER translocation and translational arrest (*Eif4ebp1*), protein folding (*Dnajb9*), and ER stress-responsive genes (*Stc2*, *Chac1*) were also downregulated (*p* < 0.05). Moreover, *Atf5*, encoding for a regulator of TXNIP expression via the PERK-eIF2α signaling pathway during ER stress, was similarly reduced.^47^ In addition, genes regulating innate immune response (*Pparg*, *Adora2b*, and *Ang*, FDR < 0.05) were downregulated by MANF (Figure 6E). Interestingly, a significant decrease in the expression of *H2-Ea* (histocompatibility 2, class II antigen E α, FDR < 0.05), encoding for a member of the major histocompatibility complex (MHC) class II genes and involved in antigen presentation to CD4+ T cells,^48^ was found in islets from STZ-injected MANF-overexpressing mice (Figure 6F). In addition, the expression of *Iapp* (islet amyloid peptide, FDR < 0.05) and *G6pc2* (islet-specific glucose-6-phosphatase catalytic subunit-related protein, *p* < 0.05), encoding for autoantigens recognized by autoreactive T cells in both human T1D patients and non-obese diabetic (NOD) mice,^49^^,^^50^ was reduced by MANF upon STZ (Figure 6F).

Top enriched BP terms in the group of 75 significantly upregulated genes by MANF were involved in cell adhesion and synaptic function (Figures 6C and 6G), as well as small GTPase-mediated and Ras protein signal transduction, related to cell survival, differentiation, adhesion, migration, and proliferation (Figures 6C and 6H). *Gli2*, a Hedgehog (Hh) signaling mediator involved in β cell differentiation and proliferation,^51^ was among the top 5 upregulated genes by MANF (Figures 6A and 6H). *Rit2* and *Rapgef4*,^52^ regulators of RAS protein signal transduction associated with cell survival and proliferation, were also upregulated by MANF (Figures 6A and 6H).

### Elevated β cell-specific MANF protects islets *in vitro* against STZ-induced ER stress, TXNIP upregulation, and inflammation

**Figure 7:**
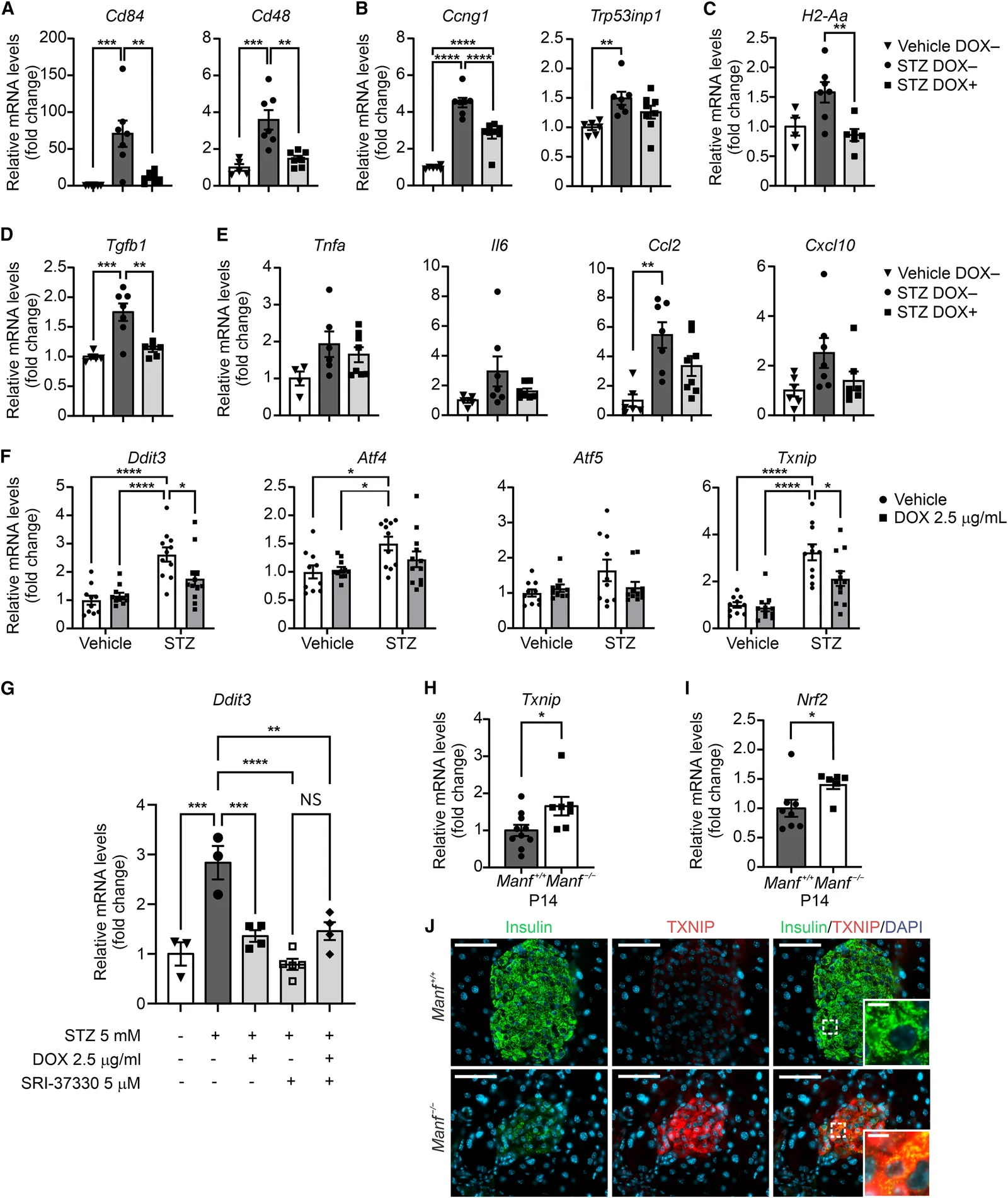
Elevated β cell-specific MANF attenuates STZ-induced expression of genes associated with immune activation, p53 targets, ER stress, and oxidative stress, whereas loss of MANF results in increased pro-inflammatory TXNIP expression and oxidative stress in islets (A–E) Validation of the mRNA levels of marker genes for immune cells (A), p53 transcriptional targets (B), MHC class II molecule *H2-Aa* (C), and cytokines and chemokines (D–E) measured by RT-qPCR in islets isolated from vehicle-injected INS-MANF DOX– mice (Vehicle DOX–, *n* = 6), MLDS-injected (45 mg/kg/day) INS-MANF DOX– (STZ DOX–, *n* = 7), and INS-MANF DOX+ (STZ DOX+, *n* = 8) mice on day 4 after the first MLDS injection. (F) Relative mRNA levels of *Ddit3*, *Atf4*, *Atf5*, and *Txnip* analyzed by RT-qPCR from isolated islets of INS-MANF mice fed with DOX+ or normal chow for 3 months. After recovery in medium ± DOX, islets were exposed to 5 mM STZ for 1 h *in vitro* (*n* = 10–12 per group). (G) Relative mRNA levels of *Ddit3* after STZ, DOX, and TXNIP inhibitor SRI-37330 treatment (*n* = 3–5 per group). (H and I) RT-qPCR of *Txnip* (H) and oxidative stress marker *Nrf2* (I) expression in P14 *Manf*^*+/+*^ (*n* = 8–10) and *Manf*^−/−^ (*n* = 7) islets. (J) Representative pictures of TXNIP (red) and insulin (green) antibody-stained pancreas sections from 5-week-old *Manf*^*+/+*^ and littermate *Manf*^−/−^ mice. Scale bars, 50 and 10 μm (insets). Data are expressed as mean ± SEM, and scatterplots show all individual data points. Statistical analyses were performed using one-way ANOVA with Tukey’s post hoc test for (A–E) and (G), two-way ANOVA with Tukey’s post hoc test for (F) and Student’s two-tailed unpaired *t* tests for (H) and (I). ∗*p* < 0.05, ∗∗*p* < 0.01, ∗∗∗*p* < 0.001, ∗∗∗∗*p* < 0.0001, NS > 0.05.

We validated our mRNA-seq data by RT-qPCR analyses of islets from a new set of STZ-injected mice using the same *in vivo* protocol as above. We confirmed that increased MANF in islets led to a reduced expression of *Cd48* and *Cd84* compared with islets from STZ-injected control mice (Figure 7A). In addition, there was a reduced expression of p53 targets, *Ccng1* and a trend toward reduced expression of *Trp53inp1* in MANF-overexpressing islets (Figure 7B). Elevated MANF in β cells also significantly attenuated STZ-induced upregulation of *H2-Aa*, a component of MHC class II (Figure 7C), and *Tgfb1* (Figure 7D), with a trend toward reduced expression of cytokines and chemokines, including *Tnfa*, *Il6*, *Ccl2*, and *Cxcl10* (Figure 7E), while the expression of *H2-Kb*, an MHC class I molecule, remained unchanged (Figure S8A). To further depict the signaling pathways underlying the protective effect of overexpressed MANF, we induced β cell dysfunction and death in primary mouse β cells using STZ *in vitro*. Islets isolated from INS-MANF DOX+ mice were cultured in the presence of DOX *in vitro*, whereas INS-MANF DOX– islets were cultured in normal medium and treated with or without STZ. In accordance with our RNA-seq data, overexpression of MANF in islets reduced the expression of UPR pro-apoptotic marker *Ddit3*, along with a trend toward decreased expression of *Atf4* and *Atf5* (Figure 7F). Additionally, MANF overexpression reduced the STZ-induced upregulation of *Txnip in vitro* (Figure 7F). This aligns with our *in vivo* observations, showing increased TXNIP staining in MLDS-injected control, compared with MANF-overexpressing β cells (Figure 4D). To further investigate the relationship between MANF and TXNIP, isolated mouse islets from INS-MANF mice were treated with STZ in the presence or absence of a TXNIP inhibitor SRI-37330^53^ or DOX. Both MANF overexpression and TXNIP inhibition attenuated STZ-induced *Ddit3* and *Txnip* expression, with no additional reduction upon combined treatment compared with either treatment alone (Figure 7G, S8B, and S8C). Notably, chronic UPR activation in MANF-deficient islets,^18^^,^^26^ was associated with increased *Txnip* and *Nrf2* expression (Figures 7H, 7I, S8D, and S8E). Furthermore, the TXNIP protein levels were significantly increased in insulin-positive β cells of 5-week-old *Manf*^−/−^ mice (Figure 7J). These results suggest that increasing MANF levels in β cells alleviate ER stress and oxidative stress, hindering TXNIP-induced terminal UPR activation, thus promoting β cell survival.

### Exogenous MANF protein protects mouse β cells against STZ-induced β cell death *in vitro* and *in vivo*

**Figure 8:**
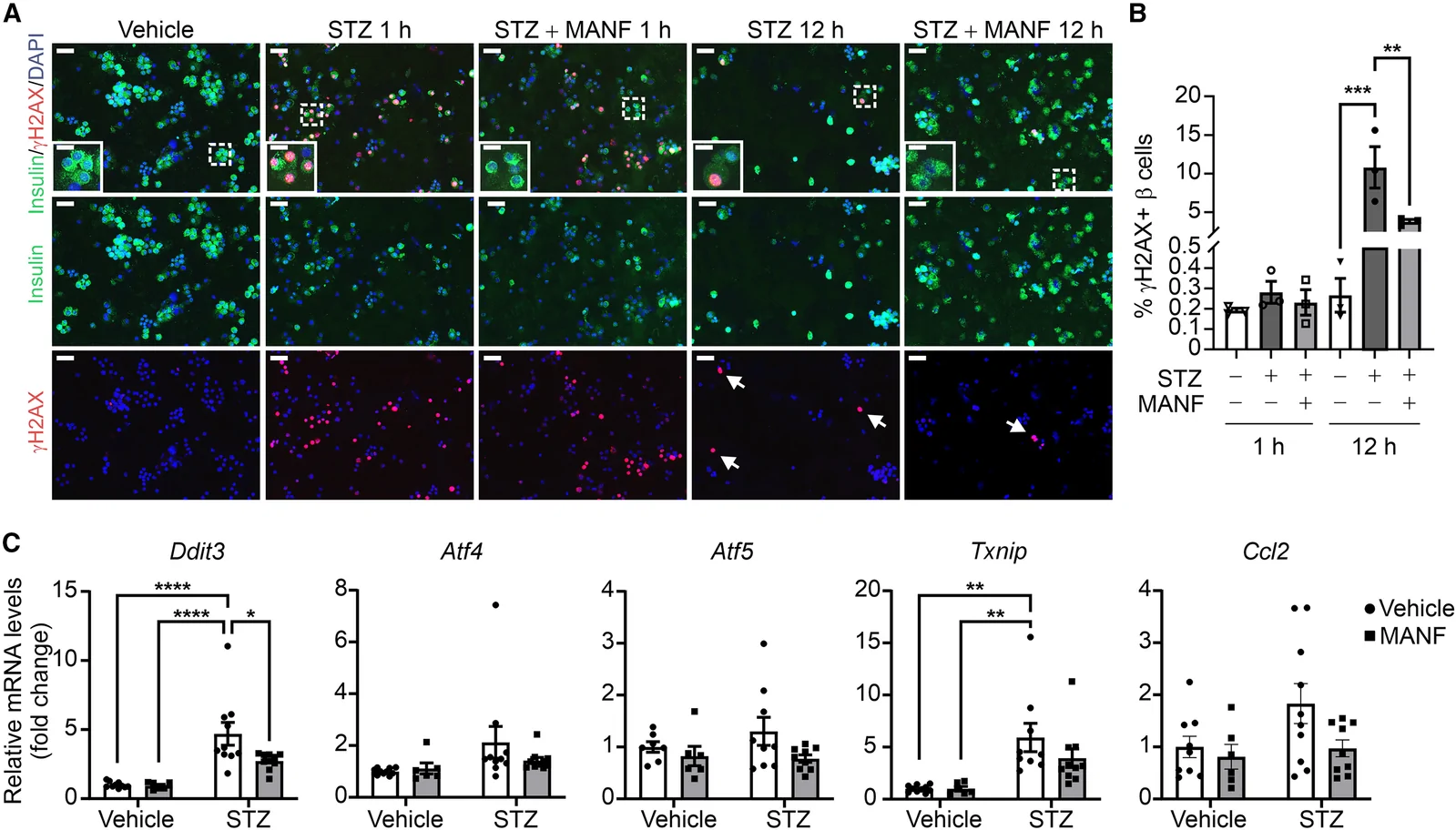
Exogenous MANF protein reduces STZ-induced β cell death, ER stress, and *Txnip* expression *in vitro* (A) Insulin-stained (green) and γH2AX antibody-stained (red) dissociated islet cells treated with 5 mM STZ in the presence or absence of 200 ng/mL MANF protein for 1 and 12 h *in vitro*. γH2AX-positive cells are marked by white arrows. Scale bars, 50 and 20 μm (insets). (B) Quantification of the ratio of γH2AX-positive β cells 1 and 12 h after STZ treatment (*n* = 3 per group). (C) Relative mRNA expression levels of *Ddit3*, *Atf4*, *Atf5*, *Txnip*, and *Ccl2* in islets isolated from WT C57BL/6JRccHsd mice stimulated simultaneously with 5 mM STZ with or without 200 ng/mL MANF protein for 1 h *in vitro* (*n* = 6–10 per group). Data are expressed as mean ± SEM, and scatterplots show all individual data points. Statistical analyses were performed using one-way ANOVA with Tukey’s post hoc test for (B) and two-way ANOVA with Tukey’s post hoc test for (C). ∗*p* < 0.05, ∗∗*p* < 0.01, ∗∗∗∗*p* < 0.0001.

Last, we aimed to study whether exogenously added rhMANF protein, similarly to endogenously overexpressed MANF, confers protection against STZ *in vitro* and *in vivo*. We first evaluated the protective effect of exogenous MANF by treating primary mouse islets isolated from adult wild-type (WT) mice with STZ in the presence of rhMANF protein *in vitro*. Quantification of γH2AX-positive β cells revealed that exogenous MANF efficiently protected against STZ-induced β cell DSBs and death compared with control (Figures 8A and 8B). Similarly, exogenous MANF was able to reduce the expression levels of *Ddit3*, and showed a trend to decrease the expression of *Atf4*, *Atf5*, and *Txnip* in islets treated with STZ (Figure 8C).^9^^,^^47^ MANF was also found to reduce the expression of the chemokine *Ccl2*, known to recruit immune cells into islets (Figure 8C).^54^^,^^55^

We next investigated whether systemic administration of MANF could, similarly to β cell-specific MANF overexpression, confer a therapeutic effect in the MLDS mouse model *in vivo*. Daily subcutaneous MANF treatment for 3 weeks after the first MLDS injection transiently reduced blood glucose levels compared with WT mice, whereas Exendin-4 (Ex-4), a glucagon-like peptide-1 receptor agonist,^56^ fully protected against diabetes as previously reported (Figures S9A and S9B). No significant changes in body weight loss were found between groups (Figure S9C). Interestingly, the β cell mass in MANF-injected mice was, similarly to the Ex-4-injected mice, significantly higher than in STZ-injected vehicle-treated mice (Figure S9D). In addition, a tendency toward increased β cell proliferation (Figure S9E), along with significantly reduced β cell death (Figure S9F), was found in the MANF-treated MLDS mice.

## Discussion

Our previous studies showed that MANF is indispensable for the survival, proliferation, and function of pancreatic β cells.^17^^,^^18^^,^^26^^,^^27^ Interestingly, variants of transcription factor *GLIS3*, associated with both T1D and type 2 diabetes, are known to impede MANF upregulation in stressed β cells resulting in more vulnerable β cells.^57^^,^^58^ These results indicate that appropriate MANF levels may protect stressed β cells from dysfunction and diabetes. Using our inducible, β cell-specific MANF-overexpressing mouse model, we first showed that MANF overexpressed in β cells effectively hindered β cell death and diabetes after STZ-induced injury, both in the harsher SHDS and the MLDS models resembling T1D. In both models, MANF overexpression preserved the β cell mass, and β cell proliferation was increased after injury compared with control mice. Thus, increased MANF possesses a β cell protective, regenerative, and therapeutic capacity after STZ-induced injury *in vivo*.^18^ Furthermore, MANF overexpression in β cells effectively also hindered β cell-mediated insulitis development in the MLDS model. Our data are supported by another report demonstrating that β cell-specific MANF overexpression by gene therapy lowered the rate of insulitis and diabetes incidence in the autoimmune NOD mouse model.^59^ In addition, immunophenotyping revealed a reduced number of adaptive immune cells, specifically CD4+ T cells, and a trend toward reduced numbers of macrophages in pLNs of MANF-overexpressing mice at day 4 after the first STZ injection. This likely resulted from attenuated β cell stress and local inflammation, supporting a protective effect of MANF in β cells, limiting early immune recruitment. These results suggest that MANF primarily protects against STZ-induced diabetes by preserving β cell mass, promoting β cell regeneration, and mitigating the local immune response.

STZ induces excess accumulation of ROS causing oxidative and ER stress, DNA damage, and apoptosis.^30^ MANF is relatively well studied for its protective effects on damaged cells through alleviation of ER stress.^20^ GO enrichment analysis from our RNA-seq data confirmed that the top pathways downregulated by MANF overexpression in islets of STZ-injected mice were related to the UPR and ER stress response. MANF overexpression was associated with reduced expression of genes in the PERK pathway, including pro-apoptotic transcription factor *Ddit3* and stress-inducible *Atf5*, known to regulate the transcription of eukaryotic translation factor 4E-binding protein 1 (*Eif4ebp1*) and *Txnip*. *In vitro*, we confirmed that both exogenous MANF protein and endogenous overexpressed MANF alleviated STZ-induced *Ddit3* and *Txnip* expression in islets. Notably, there is a regulatory interplay between the ER and oxidative stress pathways.^60^ IRE1α and PERK hyperactivation induce NRF2, a transcription factor and master regulator of antioxidant cell response.^61^^,^^62^ In addition, our RNA-seq data showed that the expression of NRF2 targets, *Hmox1* and *Steap1*, induced by STZ, was reduced by MANF overexpression, suggesting attenuated oxidative burden and downregulated compensatory antioxidant defenses. In contrast, MANF-deficient islets showed increased expression of *Nrf2* and *Txnip*. TXNIP is upregulated upon oxidative and ER stress, and binds and oxidizes thioredoxin, a part of the thiol-dependent antioxidant system.^63^ Importantly, TXNIP is a promising therapeutic target in diabetes. Verapamil, a calcium channel blocker with antidiabetic effect, has been shown to inhibit TXNIP expression.^64^ A specific TXNIP inhibitor, TIX100 (SRI-37330), rescues mice from diabetes and is entering phase 1 clinical trials.^53^ Using this inhibitor, we observed that MANF overexpression, similarly to TIX100 alone, attenuated STZ-induced *Ddit3* and *Txnip* upregulation, and that combined treatment did not result in further reduction. Our data demonstrate a previously unknown important link between MANF and TXNIP in regulating β cell stress responses. IRE1α and PERK hyperactivation elevates TXNIP expression, leading to a switch from adaptive to terminal UPR and β cell death.^9^^,^^47^ As MANF binds IRE1α and PERK, and reduces IRE1α oligomerization,^25^ our results suggest that appropriate MANF levels in β cells hinder IRE1α and PERK oligomerization, and TXNIP upregulation, thereby preventing terminal UPR, oxidative stress, and β cell death.

STZ directly induces DNA alkylation and the production of free radicals leading to DNA DSBs.^30^^,^^65^ Phosphorylation of H2AX is one of the first responses to DSBs followed by DDR and phosphorylation of transcription factor p53 to activate p53 responsive genes involved cell-cycle arrest, senescence, and apoptosis in β cells of STZ-injected mice.^66^ Importantly, our islet transcriptomics analyses revealed reduced expression of p53-responsive genes in MANF-overexpressing islets, in accordance with the reduced number of γH2AX-positive β cells in pancreas tissue of MLDS-treated MANF-overexpressing mice. In addition, exogenous MANF protein reduced TG-induced *p21* expression in islets *in vitro*. Increased expression of p21 and DDR leads to the accumulation of senescent β cells in T1D.^67^ DDR induces an inflammatory response leading to NF-κB activation and the production of pro-inflammatory cytokines and chemokines, contributing to the senescence-associated secretory phenotype (SASP), promoting the innate immune response.^68^ Our RNA-seq data show early signs of increased SASP marker expression in islets of MLDS mice (supplemental information). Importantly, transcripts for SASP markers such as *Ccl2*, *Il6* and *Tnfa* were reduced in MANF-overexpressing MLDS islets*.* In addition, the transcript for TGF-β1, a stress-induced cytokine known to accelerate p53/p21-induced senescence, was reduced.^69^ Thus, we have unique evidence that elevated MANF may lead to reduced β cell senescence in T1D. In addition, STZ-induced accumulation of p53 has been shown to inhibit Parkin leading to defect mitophagy, mitochondrial dysfunction, and reduced insulin secretion from β cells.^70^ In our MLDS model, increased MANF in β cells was found to increase the expression of Parkin (*Prkn*) and other autophagy-related genes. Thus, increased MANF may stimulate mitophagy and ER-phagy, thereby relieving cellular stress, in line with our recent study in a tubulointerstitial kidney disease mouse model.^71^

Increased MANF was shown to induce β cell proliferation following STZ-induced injury. Decreasing ER stress and UPR activation by reducing insulin expression in β cells has been shown to promote proliferation by reducing TRIB3 levels, a negative regulator of AKT.^15^ Our previous study shows that MANF deficiency in β cells increases ER stress-induced *Trib3*.^26^ Therefore, increased MANF may reduce *Trib3* expression and cell-cycle arrest by relieving ER stress, thus inducing β cell proliferation. Additionally, MANF overexpression promoted the expression of components in the RHO/RAS GTPase, RAS/MAPK, and Hedgehog signaling pathways, which are critical for insulin secretion, cell-cycle progression, adhesion, and regeneration.^51^^,^^52^ Thus, MANF does not only shield β cells from damage but also promotes their functional capacity and proliferation. Clearly, more detailed studies are needed to clarify mitogenic roles for MANF in islets.

STZ triggers a robust autoimmune and inflammatory response, as shown by increased islet expression of immune-related genes in our mRNA-seq data. Several markers for innate and adaptive immune cells and inflammation were upregulated by STZ, in contrast to MANF-overexpressing islets. This is in line with our histological findings of reduced insulitis in MANF-overexpressing islets several weeks after the first STZ injection. Immunophenotyping further revealed a reduced number of activated immune cells in the pLNs. Increased UPR activation in prediabetic T1D human and mouse β cells has been shown to upregulate human leukocyte antigen class I and II expression and increase antigen and neoantigen formation and presentation.^3^^,^^14^^,^^72^^,^^73^ Importantly, our data revealed reduced expression of antigen-presenting MHC class II molecules *H2-Ea* and *H2-Aa* in MANF-overexpressing STZ islets. In addition, expression of *G6pc2* and *Iapp*, encoding common autoantigens in T1D,^49^^,^^50^ was downregulated in islets from STZ-injected MANF-overexpressing mice compared with controls. A trend toward reduced pro-inflammatory cytokine and chemokine expression was detected in MANF-overexpressing islets, consistent with prior findings showing that MANF reduces NF-κB-regulated genes and pathways in human islets *in vitro*.^17^ Together, these results confirm that elevated MANF reduces the UPR signaling, autoantigen expression, and antigen presentation, thereby limiting adaptive T cell recruitment, accompanied by the reduced number of CD4+ T cells and macrophages in pLNs of STZ-induced prediabetic mice. Similar data were found in a collaborative study showing the protective effect of the C-terminal fragment of MANF in an *ex vivo* model of multiple sclerosis with transcriptional changes highly similar to our RNA-seq data, confirming the effect of MANF to attenuate stress- and inflammation-related gene programs.^74^

Last, we wanted to explore the protective effect of exogenous MANF compared with endogenously overexpressed MANF in β cells. Consistent with the elevated transgenic MANF, exogenous MANF protected against STZ-induced DSBs, ER stress, and *Txnip* expression in β cells *in vitro*. Despite only a transient and modest reduction in blood glucose levels upon systemic delivery of recombinant MANF *in vivo*, we detected an increase in β cell mass and reduced β cell death, indicating a spatial but insufficient therapeutic benefit in the MLDS model. Nonetheless, we highlight functional similarities between endogenous and exogenous MANF, yet also underscore important limitations related to protein stability and uptake in systemic delivery. Together, these results suggest that sustained, localized expression of MANF may be required for optimal therapeutic efficacy in preserving β cell function and preventing diabetes progression.

However, translation of β cell-specific MANF overexpression to humans is challenged by species-specific immune and metabolic differences, the multifactorial nature of human diabetes, and unresolved issues related to gene delivery, safety, and long-term efficacy. Identifying small-molecule compounds that mimic MANF activity by relieving cellular stress and TXNIP-mediated dysfunction may provide a more readily translatable therapeutic strategy. Furthermore, efforts to restore β cell mass in T1D must combine strategies that stimulate regeneration of residual β cells with approaches that attenuate the β cell-directed autoimmunity. Our data demonstrate that elevated MANF protects β cells by alleviating cellular stress, limiting stress-induced autoimmune responses, and enhancing β cell proliferation. These data position MANF as an attractive candidate for next-generation therapeutic approaches, including engineering stem cell-derived islets with increased stress resilience for replacement therapies or developing gene therapy approaches to selectively increase MANF levels in remaining human β cells.

## Materials and methods

### Animals and generation of transgenic mice

The cDNA for human MANF (540 bp) was amplified by PCR from a plasmid previously described,^75^ using the following primers, F 5′-CCT GCA GCT ACA AAT CGG TCC GTG-3′, R 5′-CGC TGC AGC TAC AAA TCG GTC CGT G-3′, cloned into pCRII (Thermo Fisher Scientific, Waltham, MA) and subcloned into a Tet-op-mp1 pBS-SK vector (a gift from Prof. Katerina Politi, New Haven, CT, and Prof. Harold E. Varmus, New York, NY).^76^^,^^77^ Functionality of the TET-On system was tested in CHO cells co-transfected with TET-MANF and PB-CA-rtTA-On vectors (Addgene, Cambridge, MA) in the presence of 2.5 μg/mL of DOX (D9891, Sigma-Aldrich, St. Louis, MO) in 70% ethanol, followed by western blotting (Figures S1C and S1D). A transgene fragment of 1,600 bp was purified and injected into fertilized oocytes from FVB mice. TET-MANF transgenic founders were identified by Southern blotting and PCR genotyping, and copy number was estimated by Southern blotting and RT-qPCR using primer sequences for human MANF F 5′-ATG ATG CAG CCA AAA TC-3′, R 5′-CAG ATC TTC TCC ACA GGG ATG-3′ (amplicon 73 nt), and actin F 5′-CAG CCA ACT TTA CGC CTA GC-3′, R 5′-GGG CCC ACG AGT GTC TAC-3′. To generate bi-transgenic INS-MANF mice, hemizygous TET-MANF mice were crossbred with hemi- or homozygous INS-rtTA mice in an FVB background (gift from Timo Otonkoski, Biomedicum Stem Cell Center, University of Helsinki, Helsinki, Finland).^78^^,^^79^ MANF overexpression was induced in β cells by a DOX diet (Envigo TD.01306 irradiated Teklad rodent diet 2018 with 625 mg/kg DOX). One line with 4–6 MANF gene copies showing the highest expression was selected.

The WT C57BL/6JRccHsd mice were purchased from Envigo, Indiana. Global *Manf*^−/−^ mice and pancreas-specific MANF KO (*Pdx-1Cre*^*+/−*^*::Manf*^*fl/fl*^) were generated as previously described.^18^^,^^26^ Since we used STZ to induce diabetes and male mice are more sensitive to STZ than female mice, likely due to hormonal differences,^30^ we focused on characterizing bi-transgenic male mice with higher β cell-specific MANF overexpression (Figure 1). MANF expression levels were about three times higher in islets isolated from male mice compared with female mice (Figures S1A–S1F).

All experimental procedures involving mice were conducted in accordance with the ARRIVE 2.0 guidelines and Declaration of Helsinki and approved by the Regional State Administrative Agency for Southern Finland, protocols ESAVI/11997/2023, ESAVI/9523/2020, ESAVI/3117/04.10.07/2017, and ESAVI/2629/04.10.07/2017.

### Genotyping

Genomic DNA was isolated from earmarks using an Extracta DNA Prep for PCR kit (95091, Quantabio, Beverly, MA) and genotyping was carried out by PCR using the following primers, TET-MANF mice: F 5′-ATC CAC GCT GTT TTG ACC TC-3′, R 5′-ACG CAG GAG TTT TGA TGG AC-3′ generating a 991-bp transgene band, INS-rtTA mice: F 5′-TAG ATG TGC TTT ACT AAG TCA TCG CG-3′, R 5′-GAG ATC GAG CAG GCC CTC GAT GGT AG-3′ generating a 400-bp transgene band. *Pdx-1Cre*^*+/−*^*::Manf*^*fl/fl*^, *Manf*^*fl/fl*^, and *Manf*^−/−^ mice were genotyped as previously described.^18^

### Blood parameters and ELISAs

Blood was collected from the tail tip for immediate glucose measurement using a clinical glucometer (Accucheck Aviva Glucometer, Roche Diagnostics, Basel, Switzerland). Fasting glucose levels were measured after 4 h fasting. At endpoint, terminal blood was collected via cardiac puncture for insulin measurement (ultrasensitive mouse insulin ELISA, 90080, Crystal Chem, Elk Grove Village, IL), and human MANF quantification in islets using an in-house sandwich ELISA specific for human MANF as previously described.^80^

### Intraperitoneal glucose tolerance tests

Intraperitoneal glucose tolerance tests were performed as previously described.^18^ Mice fasted for 6–12 h before intraperitoneal injection of glucose (2 mg/g body weight, 20%, w/v) and tail vein glucose levels were monitored at 0, 30, 60, 90, and 120 min using the Accucheck Aviva Glucometer.

### *In vivo* STZ injections and systemic MANF administration in mice

INS-MANF and sTG mice were fed with DOX or normal chow for 3 months before STZ injections. STZ (S0130, Sigma-Aldrich) was freshly diluted in a 25 mmol/L sodium citrate buffer (pH 4.5) and injected intraperitoneally at 180 mg/kg body weight as a single dose, with buffer as control. In the MLDS model, STZ was administered at 45–50 mg/kg body weight per day for 5 consecutive days. For exogenous MANF therapy, daily subcutaneous injections of MANF (P-101-100, Icosagen, Tartu, Estonia, 0.7 mg/kg/day in PBS) were started 5 days before MLDS injections and continued for 21 days in 12-week-old male C57BL/6JRcc mice. Ex-4 (E7144, Sigma-Aldrich, 0.1 mg/kg/day) was used as a positive control. Mice were maintained for an additional week without MANF or Ex-4 and sacrificed 23 days after the first STZ injection. To prevent hypoglycemia, 10% (w/v) sucrose (S0389, Sigma-Aldrich) water was provided for 1–2 days post-injection. Blood glucose levels and body weight were measured before the first STZ injection and two to three times weekly until sacrifice.

### Immunohistochemistry, immunofluorescence, and analyses of α or β cell mass

Pancreas and brain tissues were post-fixed in 4% paraformaldehyde (PFA) for 2–4 days, paraffin-embedded, and sectioned at 5 μm. Sections were subjected to antigen retrieval in 10 mM sodium citrate buffer (pH 6.0) followed by staining with primary antibodies (Table S3). Biotinylated or Alexa Fluor 488/568-conjugated secondary antibodies were used for immunohistochemistry or immunofluorescence, respectively. α and β cell mass was analyzed from four serial pancreatic sections (one per 200 μm) stained with anti-glucagon or anti-insulin antibodies, as described (Table S3), and analyzed using the Image-Pro Plus program. β Cell proliferation and STZ-induced active DDR ultimately leading to apoptosis were assessed via Ki67- or γH2AX co-staining with insulin (Table S3). Images were captured with a 3DHISTECH Panoramic 250 FLASH II (Budapest, Hungary) or a Zeiss AxioImager M2 microscope (Zeiss, Oberkochen, Germany) and analyzed with CaseViewer or ZEN 3.1 software (Zeiss).

### Insulitis scoring

Pancreases were dissected, fixed in 4% PFA for 2–4 days, paraffin-embedded, and sectioned in 5 μm sections. Four sections per pancreas, 200 μm apart, were used for hematoxylin and eosin staining to score islet insulitis. From each pancreas, 16–32 islets (4–8 randomly selected per section) were scored blindly by two independent observers. Islets (>20 cells) were classified as: (1) normal (<1% infiltration), (2) minor infiltration (1%–10%), (3) minor peri-insulitis (10%–25%), (4) peri-insulitis (25%–50%), or (5) insulitis (50%–100%). The percentage of islets in each score was calculated relative to the total evaluated.

### Islets isolation and *in vitro* treatment

Pancreases were digested with 1 mg/mL collagenase P (11213857001, Roche Diagnostics) and 0.25 mg/mL DNase I (11284932001, Roche Diagnostics), and islets were handpicked as previously described.^18^ Islets from 10- to 12-week-old C57BL/6JRcc male mice were recovered overnight in RPMI 1640 with 10% FBS and 1% PenStrep (100 U/mL penicillin and 100 μg/mL streptomycin), then starved in 0.5% BSA for 3 h. They were stimulated with or without 5 mM STZ (in 10 mM citrate buffer [pH 4.5]) with or without 200 ng/mL MANF for 1 or 12 h. After 1 h, islets were collected for RNA isolation and RT-qPCR; after 12 h, cells were dispersed, cytospun, fixed,^18^ and stained for insulin and γH2AX (Table S3) to quantify DNA damage in β cells. Islets from INS-MANF mice fed with DOX or normal chow were treated similarly for STZ experiments, followed by RNA isolation and RT-qPCR. For TXNIP inhibitor treatment, islets isolated from INS-MANF or control mice were treated with 5 mM STZ and 5 μM SRI-37330 (HY-142114, MedChemExpress, Monmouth Junction, NJ) with or without 2.5 μg/mL DOX for 1 h. For TG treatment, islets were treated with 2 μM TG (T7459, Invitrogen, Thermo Fisher Scientific) with or without MANF protein for 16 h.

### RT-qPCR

Hypothalamic tissue was collected via cryostat-assisted punches guided by Allen Mouse Brain Atlas (2008) for precise localization. RNA from islets or hypothalamic tissue was isolated by TRIzol Reagent (15596026, Invitrogen, Thermo Fisher Scientific) and quantitated by NanoDrop (NanoDrop Technologies, Wilmington, DE). Reverse transcription was performed with RevertAid Premium Reverse Transcriptase (EP0753, Thermo Fisher Scientific) and oligo(dT)18 Primer (500 μg/mL) at 55°C for 40 min. Quantitative RT-PCR was performed using LightCycler 480 SYBR Green I Master mix (04887352001, Roche Diagnostics) on a LightCycler 480 Real-Time PCR system (Roche Diagnostics, Rotkreuz, Switzerland), with expression levels normalized to β-actin. Primer sequences are listed in Table S4.

### Bulk RNA-seq

INS-MANF and sTG mice were fed with DOX or normal chow for 12 weeks, followed by MLDS (45 mg/kg body weight/day) or vehicle for 3 days. Islets were isolated on day 4, and total RNA was extracted using the QIAGEN RNeasy Micro kit (74004, QIAGEN, Hilden, Germany) according to the manufacturer’s instructions. Poly(A)-selected mRNA-seq was performed by Azenta Life Sciences (Leipzig, Germany). Data were processed on the Galaxy Community platform^81^; fastq files were quality checked with fastp v.0.23.2, aligned to the built-in mouse mm10 reference genome using HISAT2 v.2.2.1, and transcripts counted with FeatureCounts v.1.6.4. Differential gene expression analyses were performed using DESeq2 v.22.11.40.6 and gene IDs converted via SynGO portal.^82^ GO enrichment analysis was performed using DAVID,^83^^,^^84^ and graphs were created using R package ggplot2. Heatmaps in Figure 5C show relative average expression of selected genes, calculated from the 643 DEGs between STZ DOX– and vehicle DOX– mice (Table S1).

### Immunophenotyping by flow cytometry

INS-MANF and sTG mice were fed with DOX or normal chow for 12 weeks, followed by MLDS (45 mg/kg body weight/day) or vehicle injections for 3 or 5 consecutive days. pLNs and spleens were collected on days 4 or 10, and single-cell suspensions were prepared by physical dissociation through 100-μm cell strainers (Corning, New York), followed by red blood cell lysis using RBC Lysis Buffer (00-4300-54, eBioscience, Thermo Fisher Scientific) lysis. Cells (1 × 10^6^/sample) were Fc blocked using Mouse BD Fc Block (BD Biosciences, San Jose, CA) and stained with antibodies (Table S5). Data were acquired on an LSRFortessa Cell Analyzer (BD Biosciences) and analyzed with BD FACSDiva (BD Biosciences) and FlowJo software (Tree Star) using appropriate controls for gating (Figure S5).

### Statistical analysis

Data are presented as mean ± SEM. Sample size and statistics for each experiment are included in the figure legends. Statistical comparisons between two groups were performed with Student’s unpaired *t* test. One-way analysis of variance (ANOVA) followed by Tukey’s post hoc test was used for three or more groups. We conducted two-way ANOVA to evaluate the effects of two independent factors, treatment and time, followed by Bonferroni’s post hoc test for comparisons involving two groups, Tukey’s post hoc test or Holm-Šidák’s multiple comparison test when more than two groups were involved. The *p* values < 0.05 were considered significant. Statistical analyses were performed with GraphPad Prism 10.

## Data and code availability

• Data are available within the published article and supplemental information. RNA-seq data are available in Tables S1 and S2.
• Additional data are available from the corresponding author upon reasonable request.

## Acknowledgments

We thank Sari Tynkkynen for valuable technical assistance; NIH KOMP for the MANF-targeted embryonic stem cell clone used to develop the MANF knockout mice; T.O. for INSrtTA mice; Diego Balboa (University of Helsinki, Finland) for the PB-CA-rtTA-On vector; Katerina Politi (New Haven, CT) and Harold E. Varmus (New York, NY) for the Tet-op-mp1 pBS-SK vector; Mart Saarma (10.13039/100007797University of Helsinki, Finland) for valuable support and sharing reagents and equipment; Päivi Lindholm-Pulkkila (10.13039/100007797University of Helsinki, Finland) for providing in-house sandwich human MANF ELISA and critical comments; Ulla Pirvola (10.13039/100007797University of Helsinki, Finland) for use of the Zeiss AxioImager M2 epifluorescence microscope. This work was carried out with the support of 10.13039/100015735HiLIFE Laboratory Animal Center Core Facility and 10.13039/100015735HiLIFE Viikki Flow Cytometry unit, both at the 10.13039/100007797University of Helsinki, Finland. This work was supported by grants from the Research Council of Finland (10.13039/501100002341Academy of Finland 333974), the 10.13039/501100013500Finnish Diabetes Research Foundation, and Breakthrough T1D (formerly the 10.13039/100022690Juvenile Diabetes Research Foundation) (17-2013-410, 2-SRA-2018-496-A-B, 2-SRA-2022-1202-S-B). The graphical abstract was created in BioRender (https://BioRender.com/5kus9hz).

## Author contributions

H.L., T.D., E. Pakarinen, J.K., and M.L. performed *in vivo* experiments and *in vitro* islet experiments, RT-qPCR, and immunostaining. E. Palm generated the TET-MANF transgenic plasmid and tested genotyping primers. E. Pakarinen performed *in vitro* experiments, mouse brain dissections, and antibody staining of brain sections. J.K., E. Pakarinen, and M.L. isolated primary mouse islets for RNA-seq. I.T. provided expertise and practical information on RNA-seq. H.L. and T. Org analyzed and visualized RNA-seq data. T.K.K. and M.H.V. provided expertise in immunophenotyping. H.L. and J.K. performed the immunophenotyping experiment and T.K.K. analyzed the results. E.H. contributed with information for INS-rtTA mice and insulitis-scoring methodologies. T. Otonkoski provided INS-rtTA mice and the initial idea for creating MANF transgenic mice. H.L. performed image processing, statistical analysis of data and interpretation, and figure preparation. M.L. supervised the project, designed experiments, TET-MANF transgenic plasmid construct, animal breeding, performed experiments, and interpreted data. H.L. and M.L. drafted the manuscript. All authors reviewed and edited the manuscript.

## Declaration of interests

The authors declare no competing interests.

## Supplemental information

Document S1. Figures S1–S9 and Tables S3–S5

Table S1. Lists of differentially expressed genes, along with GO enrichment and KEGG pathway analyses, for the comparisons shown in Figure 5

Table S2. Lists of differentially expressed genes, along with GO enrichment and KEGG pathway analyses, for the comparisons shown in Figure 6

Document S2. Article plus supplemental information
