# Supplementary material for: Elevated MANF expression in β cells protects mice from streptozotocin-induced diabetes by attenuating islet stress and immunogenicity
Source: Mol Ther. 2026 Feb 27;34(6):3548–67. doi: 10.1016/j.ymthe.2026.02.043 (PMC13239798; doi:10.1016/j.ymthe.2026.02.043)
Supplement: Supplementary file 1 [file mmc1.pdf]

YMTHE, Volume *34*

Supplemental Information

Elevated MANF expression in β cells protects mice from streptozotocin-induced diabetes by attenuating islet stress and immunogenicity

Huini Li, Tatiana Danilova, Erik Palm, Emmi Pakarinen, Julia Kiva, Tõnis Org, Tapani K. Koppinen, Elina Hakonen, Indrek Teino, Merja H. Voutilainen, Timo Otonkoski, and Maria Lindahl

Supplemental Text

Characterization of islet early gene responses in the prediabetic MLDS model by RNA-seq

Despite the extensive use of the multiple low doses of streptozotocin (MLDS) model, the early effects of streptozotocin (STZ) treatment on the global gene expression profile in islets have remained obscure. To investigate the direct effects of STZ on islet gene expression during the early stages of diabetes, we performed bulk RNA-seq analysis on isolated islets from MANF-overexpressing or control mice administered with low-doses of STZ or vehicle (buffer). Islets were harvested on day 4 post-injection to study early transcriptional changes prior to overt β cell loss. We used STZ (S0130, Sigma-Aldrich), which proved to be highly effective in inducing β cell death and hyperglycemia within 3 weeks after the first STZ injection.[1] We reasoned that transcriptomics analyses of islets from prediabetic STZ-injected transgenic mice *in vivo* would reveal the physiological interplay between injured islet β cells and the immune system.

The effect of STZ alone was compared by transcriptional differences between islets from vehicle- and STZ-treated INS-MANF DOX– control mice. Differentially expressed genes (DEGs) were identified using DESeq2, revealing 643 genes with an absolute Log2 fold change ($|Log_2FC|$) greater than 0.5 and FDR less than 0.05 (Table S1), with 322 genes upregulated and 321 genes downregulated in response to STZ.

A heatmap was generated to highlight the expression levels of key islet genes affected by STZ alone, selected from the 643 DEGs (Figure 5C). We first confirmed that the expression of most genes critical for insulin production, processing, secretion and glucose sensing, were significantly reduced by STZ treatment, suggesting a decline in insulin production and compromised β cell function. However, the expression of genes essential for adult β cell identity and maintenance, including *Pdx1*, *Neurod1, Ucn3, Nkx6.1, Gck, Pax6* as well as progenitor cell markers, *Sox9, Hes1, Ngn3, Aldh1a3, Serpina7* did not differ significantly between groups, indicating that β cells remained present and retained their identity in our STZ model. These results suggest that our prediabetic MLDS model resembles an

early disease stage, with β cell dysfunction but not yet a complete loss of β cell identity. Conversely, similar studies have shown that at five to seven days after the first MLDS injection most of the β cell markers are significantly downregulated with vast β cell dysfunction and death.[2]

Gene Ontology (GO) enrichment analyses of 322 upregulated DEGs by STZ using DAVID tool revealed that biological processes (BP) regulating inflammatory and innate immune responses, chemokine-mediated signaling, tumor necrosis factor production, reactive oxygen species metabolic processes, DNA damage response (DDR), and intrinsic apoptotic signaling in response to DNA damage by p53 class mediator, were among the top enriched terms (Figure 5A and Table S1). In addition, KEGG pathway analyses of the 322 upregulated DEGs by STZ revealed that the p53 signaling pathway was the most enriched (Figure S7E and Table S1). STZ directly leads to DNA Alkylation damage.[3] DNA damage response genes are clearly increased in the islets of our STZ-induced diabetic mouse model (*Gadd45a, Parp3, Ddias, Zc3h12a, Pierce1*) as well as p53-mediated cell-cycle arrest and apoptosis genes (*Tnfrs10b, Bax, Bcl3, Casp7, Bbc3 (PUMA), Ccng1, Trp53inp1, Trp53cor1, Wee1, Zmat3, Mdm2*). These features are shared with increased replicative senescence (*Cdkn1a*). Persistent DDR leads to chromatin reorganization (*H2aj, H4c8, H1f3*) and acquisition of a secretome composed of cytokines (*Il6*, $Log_2(FC) < 0.5$), chemokines (*Ccl2, Cxcl1*), growth factors (*Igfbp3*), extracellular matrix factors (*Mmp12*) and other senescence-associated secretory phenotype (SASP) factors (*Serpine1*), and p21-associated secretory phenotype (PASP) factors (*Csf1, Ctsd*).[4] Conversely, GO analysis of 321 downregulated genes by STZ highlighted the top BPs involving positive regulation of gene expression, cell adhesion, extracellular matrix organization, intracellular calcium ion homeostasis, and monoatomic cation transmembrane transport (Figure 5B and Table S1). KEGG pathway analyses further revealed the biosynthesis of amino acids as the top downregulated pathway (Figure S7F and Table S1). In addition, oxidative stress response (*Dcxr, Hmox1, Nfe2l2*) was highly upregulated in response to STZ treatment (Figure 5C), while the expression of UPR genes was not significantly altered at this early stage. Thus, STZ-induced DNA alkylation damage triggers

a robust p53-mediated DNA damage response in β cells, driving cell cycle arrest, apoptosis, and the establishment of a potent senescence-associated inflammatory phenotype characterized by SASP/PASP factor expression. Concurrently, a significant reduction of gene expression and amino acid biosynthesis drives progressive β cell dysfunction and decline in our prediabetic MLDS model. Furthermore, despite the initial DNA damage, β cell dysfunction and p53-mediated cell death, immune response was the most significantly enriched GO pathway from the upregulated genes (Figure 5A and Table S1). The heatmap showed that the expression of genes involved in immune system processes (*Cd84, Jak3, Il4ra, Tap1*), inflammatory response (*Il1rn, Tlr13, Tlr8, Stat3*) and chemokine-mediated signaling pathways (*Ccl2, Cxcl1, Ccr1, Ccl9*) were highly upregulated in response to STZ treatment (Figure 5C). These upregulated genes may be derived from intrinsic β cell inflammation, SASP and invading immune cells. Overall, these results suggest that the prediabetic MLDS model resembles key initiating events of β cell dysfunction, destruction and local immune response associated with T1D β cell pathology as previously reported.[1,5,6]

Supplemental Figures

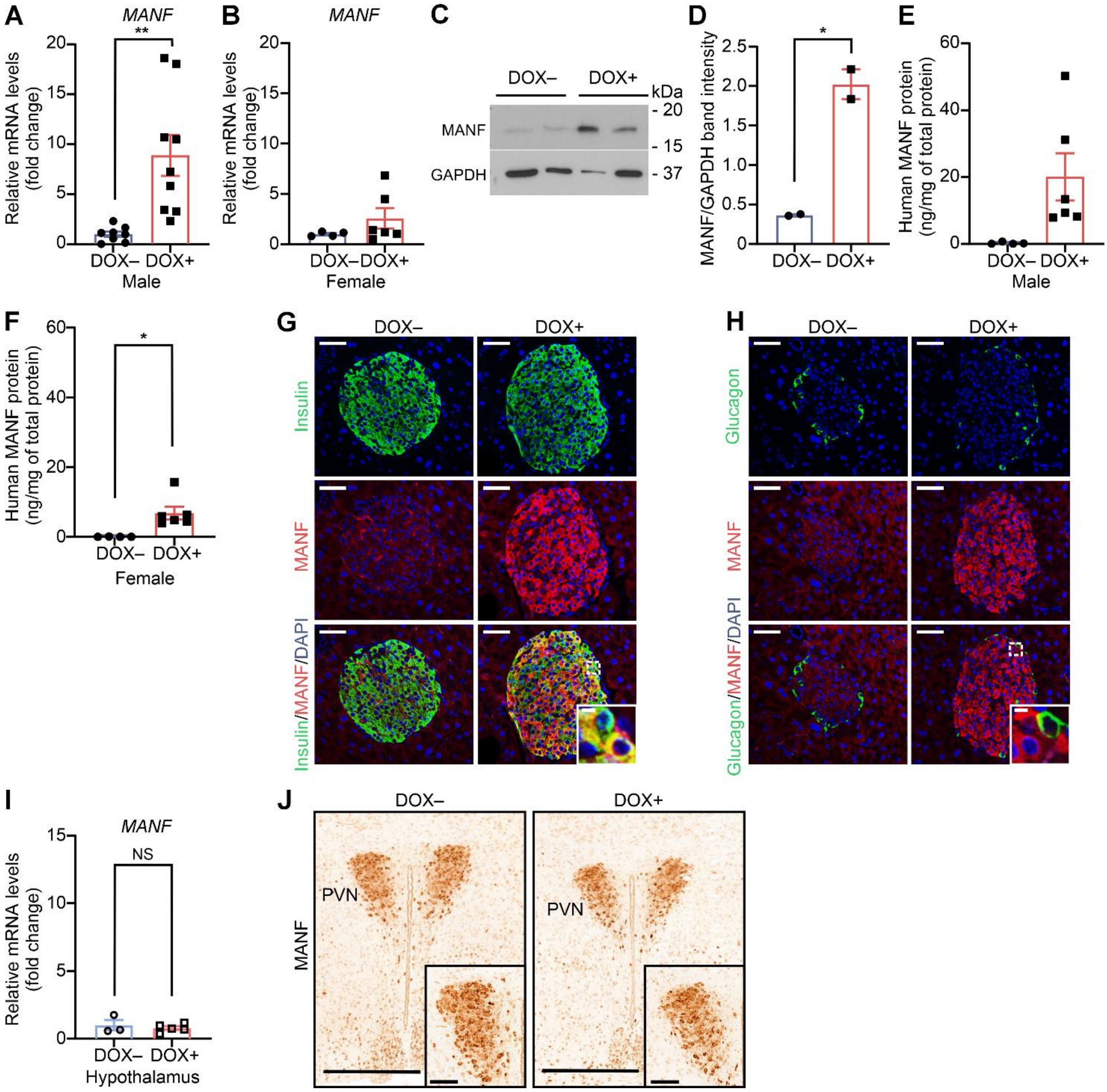

Figure S1. MANF is overexpressed in insulin-positive β cells, but not in glucagon-positive α cells nor the hypothalamic regions of male INS-MANF DOX+ mice. (**A**) A 8.9-fold increase in human *MANF* mRNA levels in β cells of islets from MANF-overexpressing (DOX+, *n* = 9) male mice compared to INS-MANF DOX– (DOX–, *n* = 8) islets, quantified by RT-qPCR. (**B**) Islets isolated from female DOX+ mice express less transgenic human *MANF* (*n* = 6) compared to male islets but 2.6-fold more than control DOX– (*n* = 4) female islets. (**C** and **D**) Representative Western blot showing a 4-fold increase in MANF protein levels in CHO cells transfected with TET-MANF construct and a PB-CA-rtTA-On vector in the presence and absence of

doxycycline ($n = 2$ per group). (**E** and **F**) Human MANF levels measured by human-specific MANF ELISA in the DOX+ islets from (**E**) male ($n = 6$) and (**F**) female ($n = 6$) mice compared to DOX– male (**E**, $n = 4$) and female mice (**F**, $n = 4$). (**G** and **H**) Transgenic MANF is expressed in β cells (**G**, yellow) but not in glucagon-positive α cells (**H**, green) in the islets of DOX+ mice shown by immunofluorescence staining of pancreas. Nuclei with DAPI (blue). Scale bar, 50 µm, 5 µm (insets). (**I**) Human *MANF* mRNA expression was not increased in punches dissected from hypothalamic regions of male mice (DOX+, $n = 5$; DOX–, $n = 3$). (**J**) MANF immunohistochemistry on brain sections showed no difference in intensity of staining between DOX+ and DOX– mice. Scale bar, 500 µm, 100 µm (insets). Data are expressed as mean ± SEM, and scatter plots show all individual data points. Statistical analyses were performed using Student's two-tailed unpaired *t*-tests. *$p < 0.05$, **$p < 0.01$, NS > 0.05.

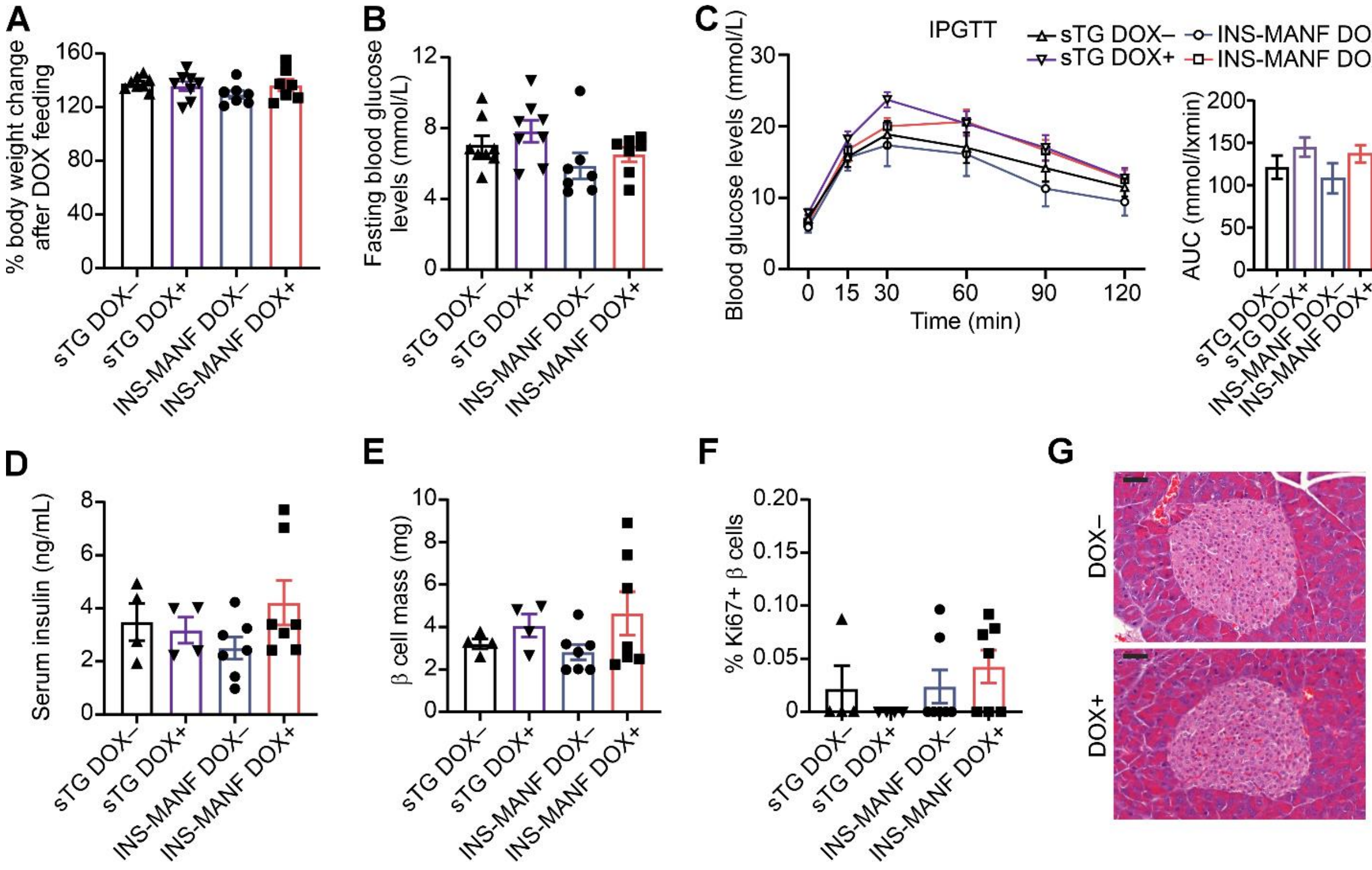

Figure S2. Prolonged MANF overexpression does not lead to significant changes in β cell proliferation nor β cell mass in male INS-MANF DOX+ mice. (**A**) No difference in the body weight change (%) between INS-MANF male mice on doxycycline (DOX) chow for 5 months (INS-MANF DOX+, *n* = 7) compared to INS-MANF mice on normal chow (INS-MANF DOX–, *n* = 7), and control single-transgenic (sTG) mice, on normal (sTG DOX–, *n* = 8), and DOX chow (sTG DOX+, *n* = 8). (**B**) No significant difference in fasting blood glucose levels. (**C**) Intraperitoneal glucose tolerance test (IPGTT) and area under the curve (AUC) of the mean blood glucose levels for each group revealed no significant difference in glucose clearing. (**D**) A trend for increased serum insulin levels in male INS-MANF DOX+ mice (*n* = 7), compared to control mice (sTG DOX–, *n* = 4; sTG DOX+, *n* = 4; INS-MANF DOX–, *n* = 7). (**E** and **F**) Quantification of the β cell mass from insulin antibody-stained sections after 5 months DOX-feeding revealed a trend for increased β cell mass (**E**) and β cell proliferation (**F**) in INS-MANF DOX+ male mice, compared to control mice. (**G**) Hematoxylin-eosin-stained pancreas sections showed no obvious pathological changes in pancreases of INS-MANF mice fed for one year with DOX-containing chow, compared to control. Scale bar, 50 μm. Data are expressed as mean ± SEM, and scatter plots show all individual data points. Statistical analyses were performed using one-way

ANOVA followed by Tukey's post hoc test for (**A**), (**B**), AUC in (**C**), (**D**–**F**) and two-way ANOVA with Tukey's post hoc test for different time points in (**C**).

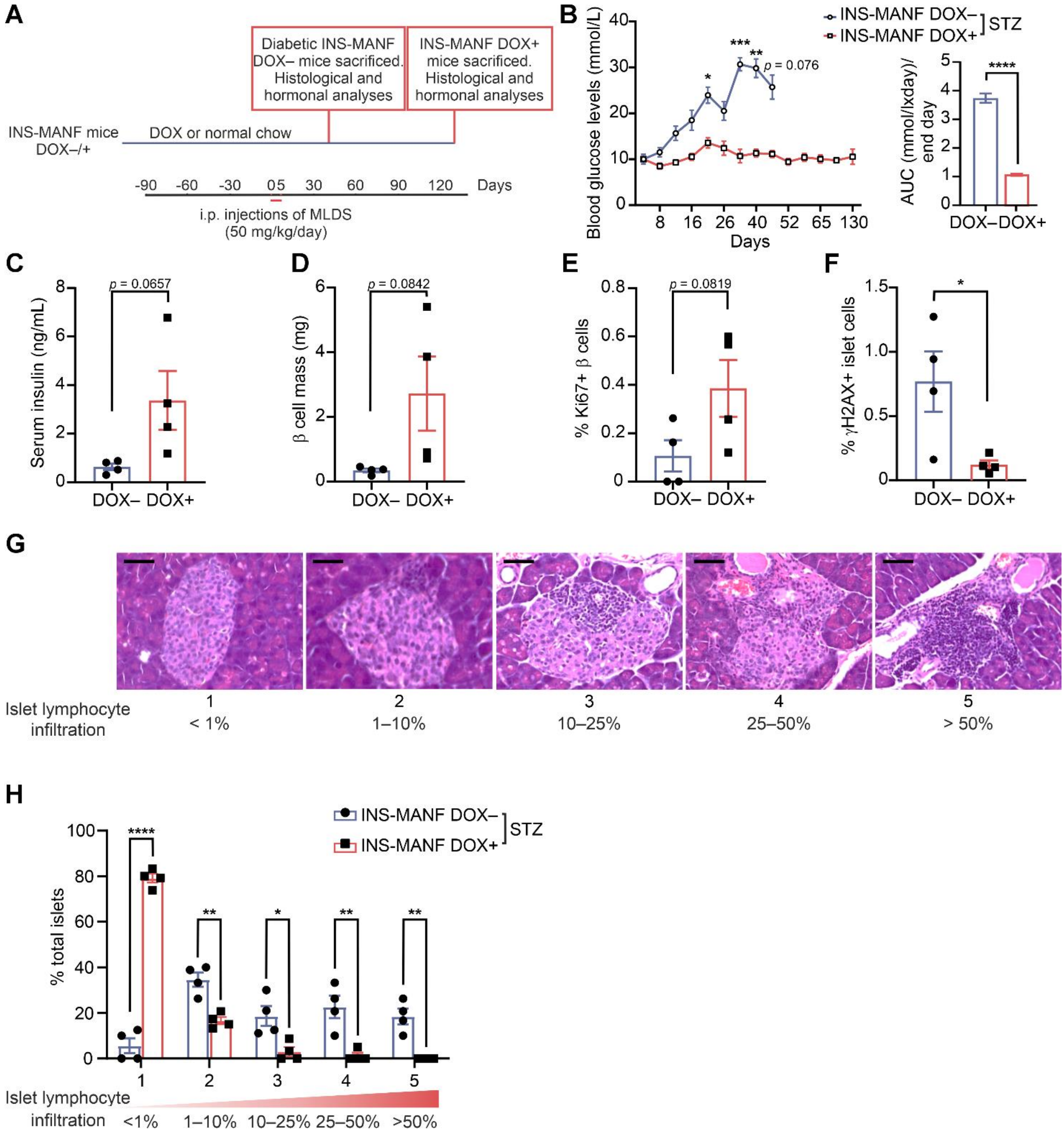

Figure S3. MANF overexpression in β cells restores normoglycemia over the long term associated with reduced insulitis. (**A**) Timeline for the doxycycline (DOX) chow-induced MANF overexpression in β cells 90 days before the multiple low dose STZ injections (MLDS, 50 mg/kg/day) followed by 129 days of DOX+ feeding of INS-MANF mice (DOX+, $n = 4$). Control INS-MANF DOX– mice (DOX–, $n = 4$) were sacrificed 45 days after the first STZ injection. (**B**) Transient small increase in blood glucose levels of DOX+ mice after

MLDS returned to normal about 50 days after the first STZ injection and area under the curve (AUC) of the mean blood glucose levels for each group. (**C**) *Ad libitum* serum insulin levels and (**D**) β cell mass in MLDS injected DOX+ compared to DOX– mice. The lack of significance might be due to variable levels of MANF-overexpression in DOX+ mice. (**E**) A trend for increased β cell proliferation quantified from Ki67 and insulin antibody-stained pancreas sections in MLDS injected DOX+ compared to DOX– mice. (**F**) Significant less γH2AX positive islet cells in pancreases from MLDS injected DOX+ compared to DOX– mice. (**G**) Representative pictures of hematoxylin-eosin-stained pancreas sections showing different grades of islet lymphocyte infiltration. Scale bar, 50 µm. (**H**) Islet insulitis scores in hyperglycemic DOX– and DOX+ mice. Data expressed as mean ± SEM, and scatter plots show all individual data points. Statistical analyses were performed using a two-way ANOVA followed by Bonferroni's post hoc test for different time points in (**B**), Student's two-tailed unpaired *t*-tests for AUC in (**B**), (**C**–**F**) and different scores for (**H**). *$p < 0.05$, **$p < 0.01$, ***$p < 0.001$, ****$p < 0.0001$.

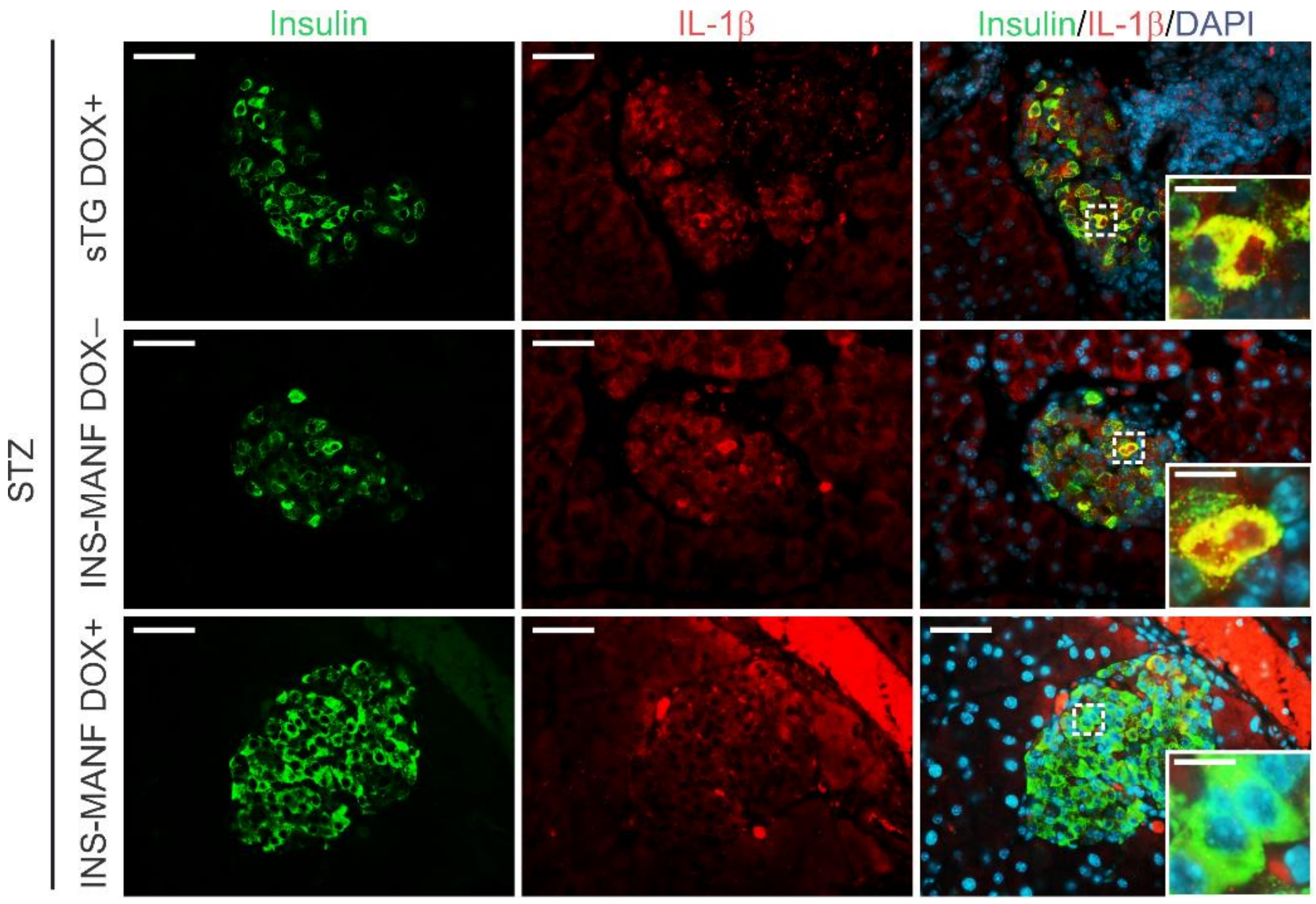

Figure S4. IL-1β staining of pancreatic sections from MLDS injected MANF-overexpressing mice compared to control mice. Representative images of IL-1β (red) and insulin (green) staining from MANF overexpressing and control mice in the MLDS model. Scale bar, 50 µm, 10 µm (insets).

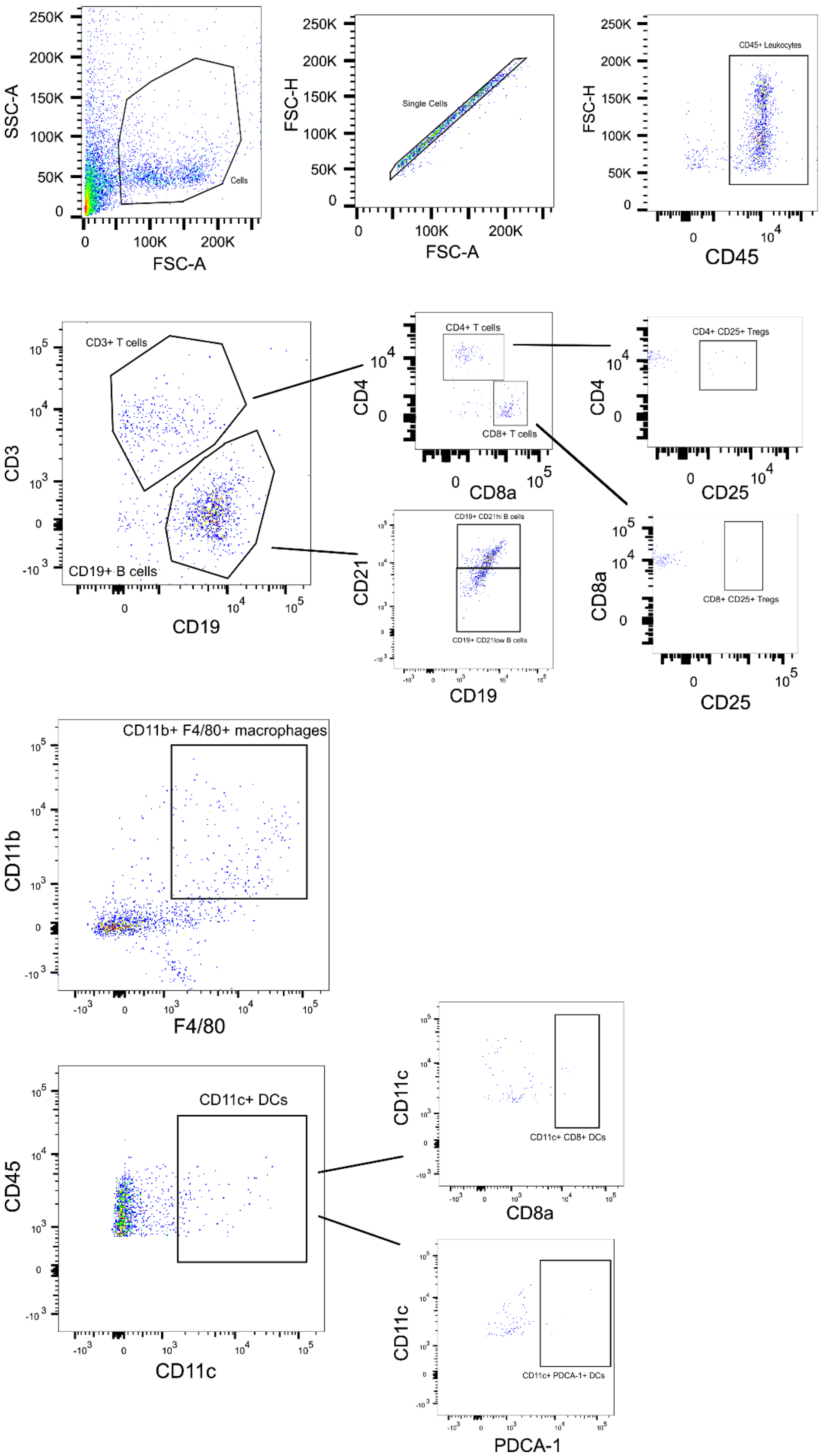

Figure S5. Flow cytometry gating strategy for immunophenotyping analysis.

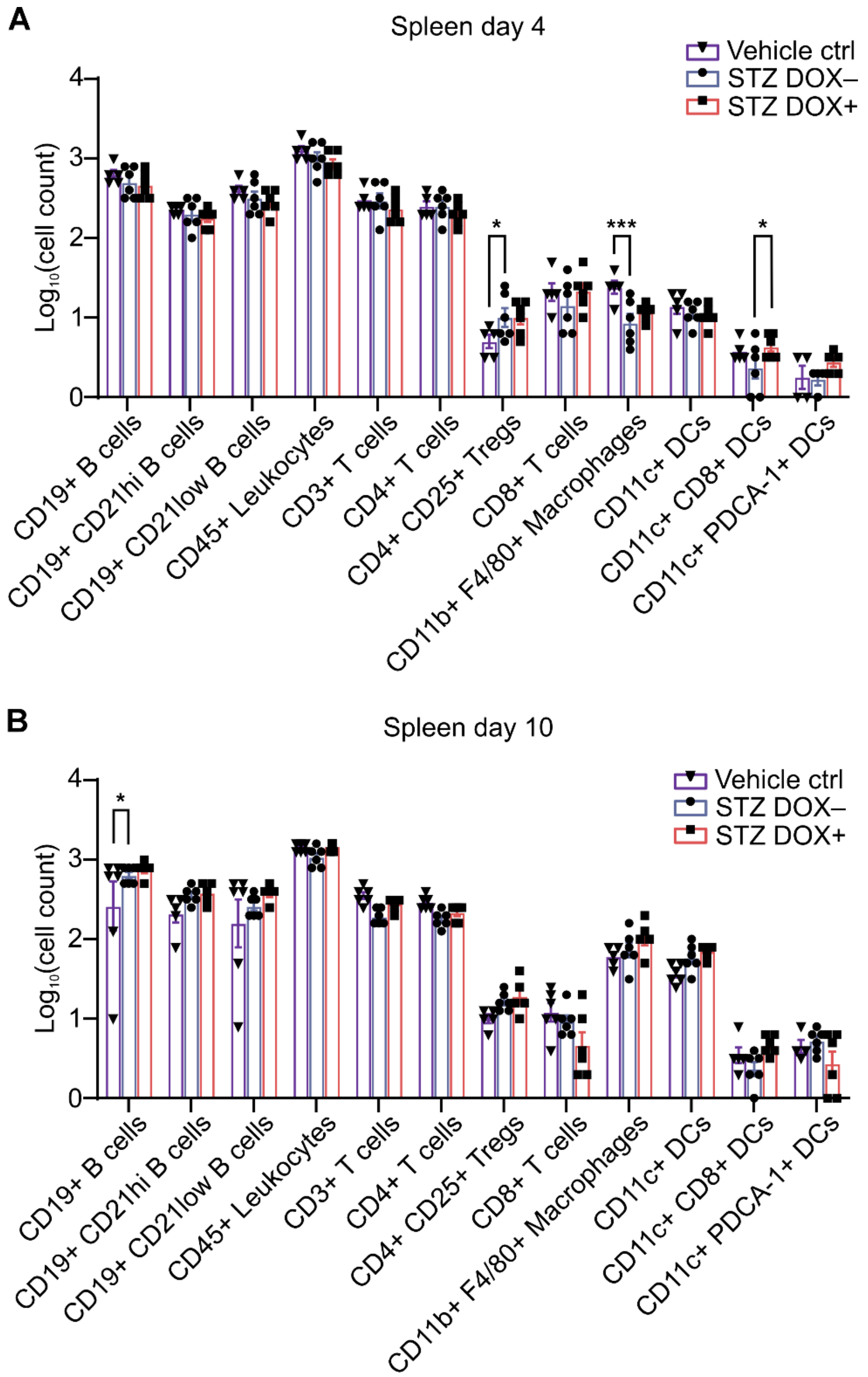

Figure S6. Immunophenotyping analysis of spleen tissue. (**A** and **B**) Immune cell profiling of spleens on day 4 (**A**) and 10 (**B**) after the first STZ (45mg/kg/day) or vehicle injection. Treatment groups include non-diabetic control mice injected with vehicle (Vehicle ctrl, *n* = 5), triangles, purple bar including TET-MANF DOX– (*n* = 1), INSrt-TA DOX– (*n* = 2) and INS-MANF DOX– (*n* = 2) mice treated with STZ (STZ DOX–, *n* = 6), circles, blue bar and INS-MANF DOX+ mice treated with STZ (STZ DOX+, *n* = 6), squares, red bar. Data expressed as mean ± SEM, and scatter plots show all individual data points. Statistical analyses were

performed using two-way ANOVA followed by Holm-Sidak's multiple comparison test. *$p < 0.05$, ***$p < 0.001$.

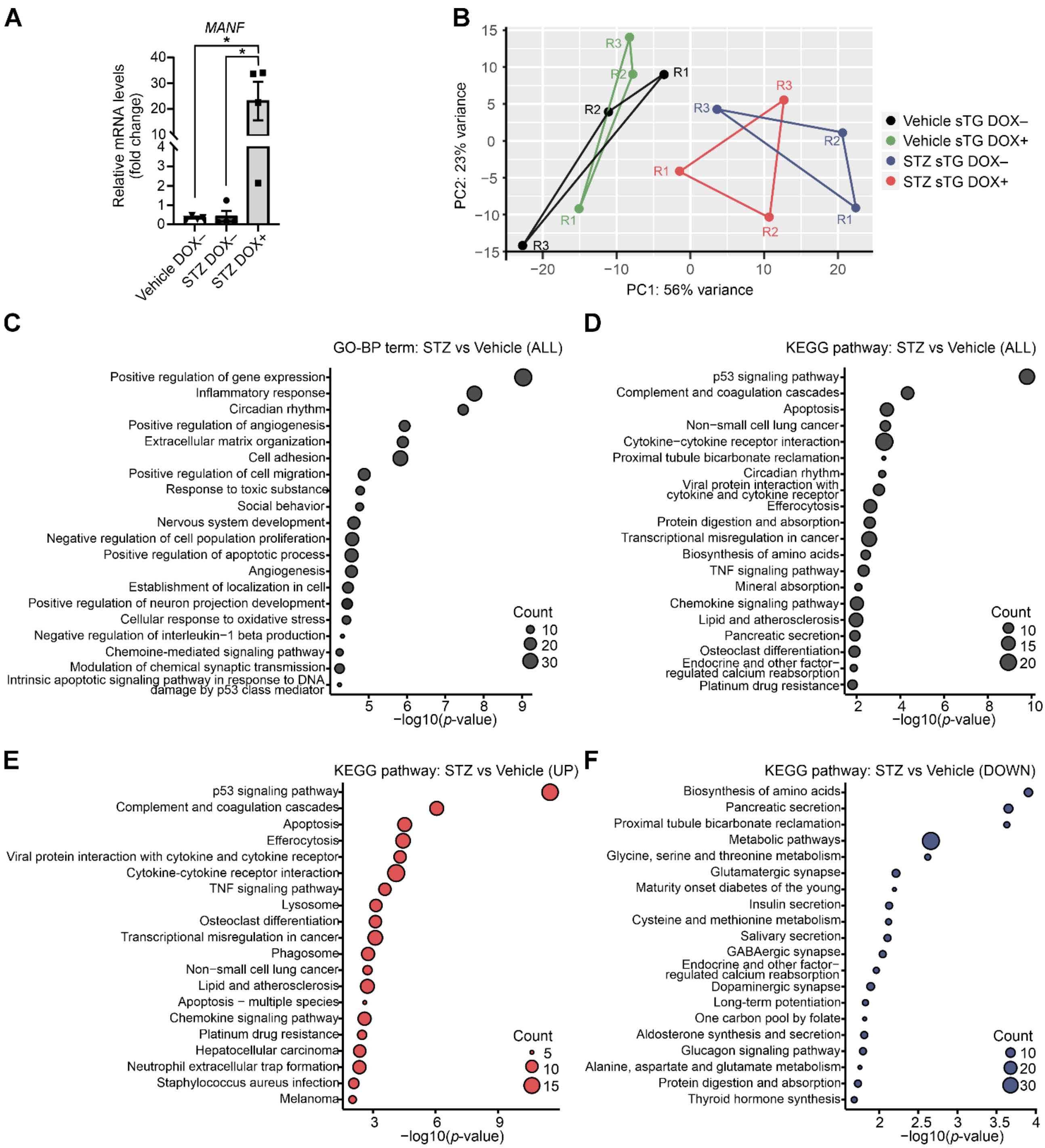

Figure S7. GO-BP terms and KEGG pathway enrichment analysis of differentially expressed genes in islets between STZ- and vehicle-injected INS-MANF DOX– control mice. (**A**) The mRNA levels of human *MANF* in the pancreatic islets of vehicle and STZ-injected INS-MANF DOX+/– mice used in RNA-seq (n = 4 per group), quantified by RT-qPCR. (**B**) PCA of all 12 sTG samples with 3 replicates (R1, R2, R3) in each 4 groups either treated with STZ or vehicle, fed by DOX+ or normal chow (DOX–) to represent the distribution

and relationships of samples in a high-dimensional space reduced to two principal components, PC1 and PC2 ($n = 3$ per group). No major changes in the overall gene expression in islets from mice fed by doxycycline diet compared to controls in the RNA-seq data. Each treatment group is presented in different color indicated in the figure. (**C**) Bubble diagrams of the GO enrichment analyses for BP terms by DAVID, on islet mRNA using 643 significantly altered DEGs with |Log2(FC)| > 0.5 and FDR < 0.05 on the effect of STZ in INS-MANF DOX– islets (STZ DOX–, $n = 4$) compared to islets from buffer treated mice (Vehicle DOX–, $n = 4$). (**D**–**F**) KEGG pathways analyses with all 643 altered DEGs (**D**), 322 significantly upregulated DEGs (**E**) and 321 downregulated DEGs (**F**) with $|Log_2(FC)| > 0.5$ and FDR < 0.05 by STZ alone between Vehicle DOX– ($n = 4$) and STZ DOX– ($n = 4$), analyzed by DAVID. Data expressed as mean ± SEM, and scatter plots show all individual data points. Statistical analyses were performed using one-way ANOVA followed by Tukey's post hoc test for (**A**). *$p < 0.05$.

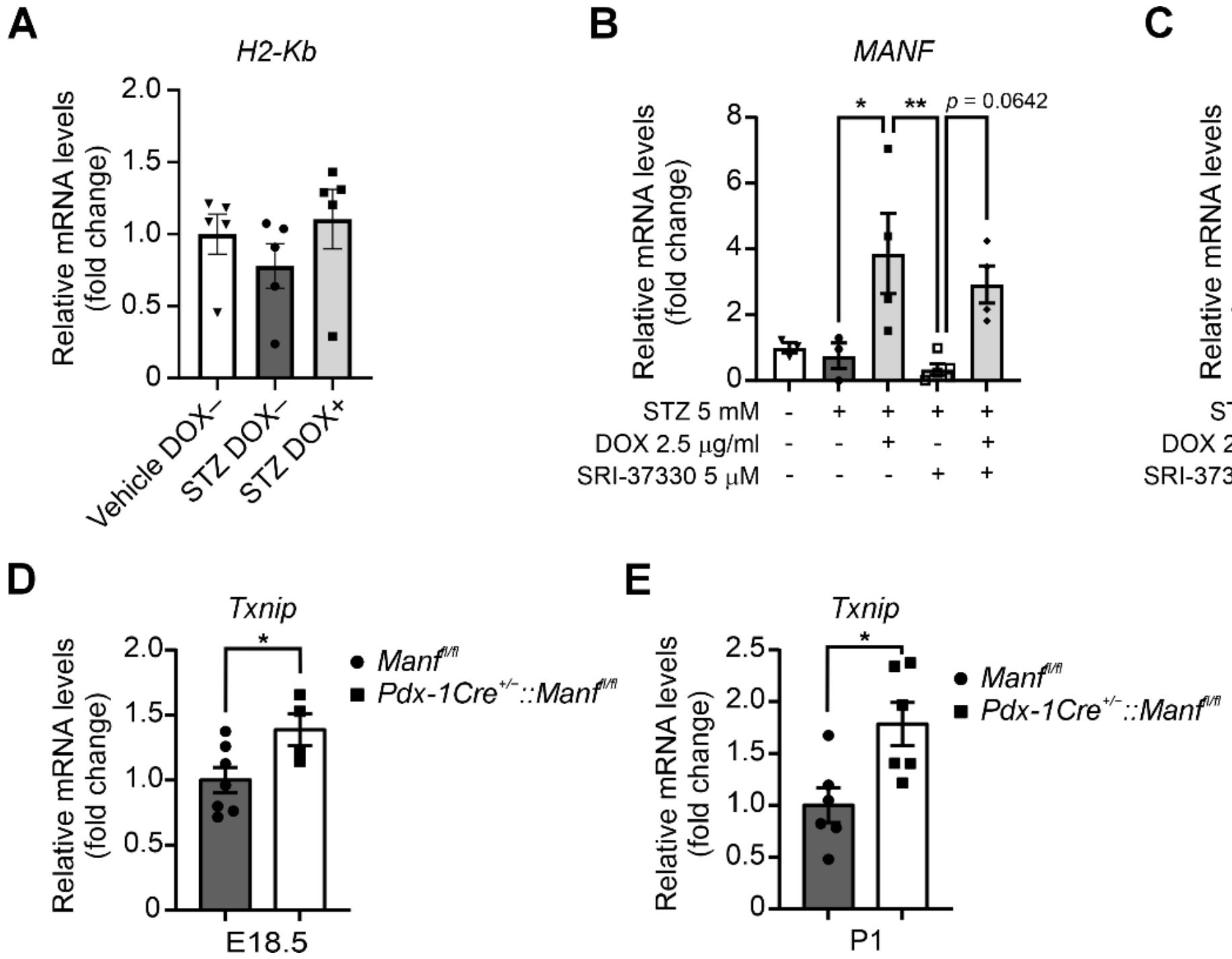

Figure S8. TXNIP mRNA levels are already increased in the pancreas and islets of embryonic and newborn MANF-deficient mice. (**A**) Relative mRNA levels of the MHC I molecule *H2-Kb*, measured by RT-qPCR in islets isolated from vehicle or MLDS-injected INS-MANF DOX+/– mice on day 4 after the first MLDS injection (n = 5 per group). (**B** and **C**) Relative mRNA levels of *MANF* (**B**) and *Txnip* (**C**) after STZ, DOX and TXNIP inhibitor SRI-37330 treatment (n = 3–5 per group). (**D**) *Txnip* mRNA expression in the E18.5 pancreas ($Manf^{fl/fl}$, n = 7; $Pdx\text{-}1Cre^{+/-}::Manf^{fl/fl}$, n = 4) and (**E**) in islets of newborn $Manf^{fl/fl}$ (n = 6) and pancreas-specific $Pdx\text{-}1Cre^{+/-}::Manf^{fl/fl}$ (n = 6) mice. Data expressed as mean ± SEM, and scatter plots show all individual data points. Statistical analyses were performed using one-way ANOVA followed by Tukey's post hoc test for (**A**–**C**). Student's two-tailed unpaired t-tests for (**D**) and (**E**). $*p < 0.05$, $**p < 0.01$.

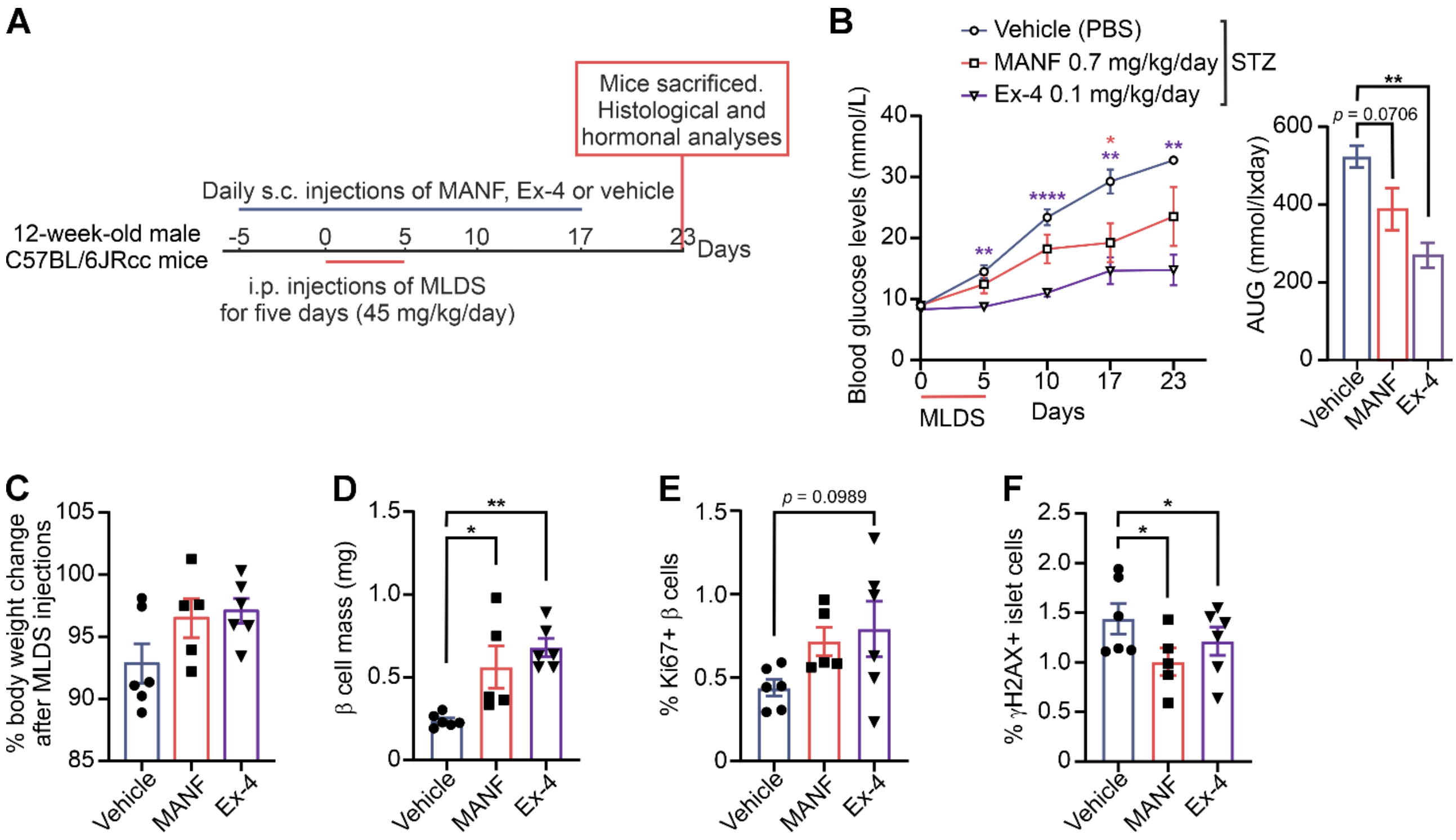

Figure S9. Systemic delivery of recombinant human MANF protein protects against MLDS-induced β cell loss *in vivo*. (**A**) Schematic illustration of the daily subcutaneous (s.c.) injections of MANF (0.7 mg/kg/day), Exendin-4 (Ex-4, 0.1 mg/kg/day) or vehicle (PBS) administered to 12-week-old male C57BL/6 mice starting 5 days before the first STZ intraperitoneal (i.p.) injection (45 mg/kg/day) and continuing until 23 days after the first injection (n = 6 per group). (**B**) *Ad libitum* fed blood glucose levels of MLDS-injected mice with vehicle, MANF or Ex-4 daily injections and area under the curve (AUC) of the mean blood glucose levels for each group. (**C**) Percentage in body weight change at the time of sacrifice compared to the body weight before the first STZ injection (vehicle, n = 6; MANF, n = 5, one mouse died due to an injection error at day 16; Ex-4, n = 6). (**D**) β cell mass quantified from insulin-stained pancreas sections of mice sacrificed 23 days after first STZ injection. (**E**) β cell proliferation quantified from insulin and Ki67 antibody–stained sections in STZ-treated mice. (**F**) Islet cell death assessed from anti-γH2AX antibody-stained pancreas sections of STZ-injected mice. Data expressed as mean ± SEM, and scatter plots show all individual data points. Statistical analyses were performed using two-way ANOVA with Holm-Sidak's multiple comparison test for the different time points in (**B**) and one-way ANOVA with Tukey's post hoc test for AUC in (**B**), (**C–F**). *p < 0.05, **p < 0.01. ****p < 0.0001.

Supplemental Tables

Table S1. Lists of differentially expressed genes, along with GO enrichment and KEGG pathway analyses, for the comparisons shown in Figure 5.

Available as a separate Excel file.

Table S2. Lists of differentially expressed genes, along with GO enrichment and KEGG pathway analyses, for the comparisons shown in Figure 6.

Available as a separate Excel file.

Table S3. List of antibodies.

Target	Catalog No.	Host Species	Clonality	Species Reactivity	Company	Application
MANF	HPA011175	Rabbit	Polyclonal	Human	Sigma-Aldrich	IF, IHC 1:200
TXNIP	ab188865	Rabbit	Monoclonal	Mouse, Rat, Human	Abcam	IF 1:400
GLUT2	sc-7580	Goat	Polyclonal	Mouse, Rat, Human	Santa Cruz	IF 1:200
Insulin	I2018	Mouse	Monoclonal	Mouse, Rat, Human	Sigma-Aldrich	IHC, ICC 1:1000
Insulin	ab181547	Rabbit	Monoclonal	Mouse, Rat, Human	Abcam	IF 1:500
Insulin	A0564	Guinea pig	Polyclonal	Mouse, Rat, Human	Dako	IF, IHC 1:3000
Glucagon	G2654	Mouse	Monoclonal	Feline, Canine, Mouse, Guinea pig, Rat, Rabbit, Human, Pig	Sigma-Aldrich	IF, IHC 1:500
CD45	ab10558	Rabbit	Polyclonal	Mouse, Rat, Human	Abcam	IF 1:200
IL-1β	ab9722	Rabbit	Polyclonal	Mouse, Human	Abcam	IF 1:100
GAPDH	MAB374	Mouse	Monoclonal	Canine, Human, Mouse, Rat, Rabbit, Fish, Feline, Pig	Sigma-Aldrich	WB 1:500
Ki67 (Clone SP6)	RM-9106	Rabbit	Monoclonal	Mouse, Human	Thermo Fisher Scientific	IF 1:200
Phospho-Histone γH2A.X (Ser139) (20E3)	9718	Rabbit	Monoclonal	Mouse, Rat, Human, Monkey	Cell Signaling Technology	IHC, ICC 1:400
MANF	310-100, Batch A12ManfPab-005, 0.45 mg/ml	Rabbit	Polyclonal	Human, Mouse	Icosagen	WB 1:500

Table S4. List of primers used for RT-qPCR.

Gene	Species	Forward primer	Reverse primer
MANF	human	GGC AAA GAG AAT CGG TTG TG	CAG ATC TTC TCC ACA GGG ATG
Manf	mouse	GAC AGC CAG ATC TGT GAA CTA AAA	TTT CAC CCG GAG CTT CTT C
Actin	mouse	CTA AGG CCA ACC GTG AAA AG	ACC AGA GAC ATA CAG GGA
Ddit3	mouse	CCA ACA GAG GTC ACA CGC AC	TGA CTG GAA TCT GGA GAG CGA
Atf4	mouse	ATG GCC GGC TAT GGA TGA T	CGA AGT CAA ACT CTT TCA GAT CCA TT
Atf5	mouse	AGG TGT ATA AGG CCC GAA GC	AGT GTG ACG CTG GAG ACA GA
Txnip	mouse	TCA AGG GCC CCT GGG AAC ATC	GAC ACT GGT GCC ATT AAG TCA G
Glut2	mouse	TGA GCA GAA GGT CTC CGT GA	TGT CGG TAA TTG GCA TCC GT
Ins1/2	mouse	AGC ATC TTT GTG GTC CCC AC	CCC CAC ACA CCA GGT AAA GAG
Pdx1	mouse	GAA ATC CAC CAA AGC TCA CG	CGG GTT CCG CTG TGT AAG
Ccl2	mouse	GCT GGA GCA TCC ACG TGT T	ATC TTG CTG GTG AAT GAG TA
Tnfa	mouse	GGA ACT GGC AGA AGA GGC ACT C	GCA GGA ATG AGA AGA GGC TGA GAC
Il6	mouse	TAG TCC TTC CTA CCC CAA TTT CC	TTG GTC CTT AGC CAC TCC TTC
Cxcl10	mouse	TCC TTG TCC TCC CTA GCT CA	ATA ACC CCT TGG GAA GAT GG
Nrf2	mouse	CAT GAT GGA CTT GGA GTT GC	CCT CCA AAG GAT GTC AAT CAA
Ccng1	mouse	GCG TTG GAG ATC CAA GCA CTG A	GGA AAC AAG CTC TTG CCA GAA GG
Trp53inp1	mouse	CTT CTC CTG TTT ACC TGC ATC TT	TGA TAG TGG TTA ATC CAC CTG CT
Cd48	mouse	GTA CAA AGA CAA TCT TCG AGT C	GCA CTC TCA TGT AGT AGG TAC
Cd84	mouse	ATA TAG CTG GAG TCC CTT TGG AG	AAA GAG CAC GGC CAA TCC TC
H2-Aa	mouse	CAA CCG TGA CTA TTC CTT CC	CCA CAG TCT CTG TCA GCT C
H2-Kb	mouse	GTG ATC TCT GGC TGT GAA GT	GTC TCC ACA AGC TCC ATG TC
Tgfb1	mouse	TGG AGC AAC ATG TGG AAC TC	CAG CAG CCG GTT ACC AAG
sXbp1	mouse	GAG TCC GCA GCA GGT G	GTG TCA GAG TCC ATG GGA
Trp53	mouse	CGC GGG CGT AAA CGC TTC GA	TCT TCA GGT AGC TGG AGT GAG CCC
Cdkn1a	mouse	TCG CTG TCT TGC ACT CTG GTG T	CCA ATC TGC GCT TGG AGT GAT AG
Trib3	mouse	CGC TTT GTC TTC AGC AAC TGT	TCA TCT GAT CCA GTC ATC ACG

Table S5. List of antibodies used for flow cytometry.

Target	Fluorophore	Isotype	Cell type	Catalog No.	Manifacturer
Zombie NIR	Zombie NIR		dead/alive cells	423106	Biolegends
CD45	FITC	Mouse IgG1, κ	leukocytes	157608	Biolegend
CD3	BV750	Rat IgG2b, κ	T-lymphocytes	100249	Biolegend
CD4	BV421	Rat IgG2b, κ	helper T cells	100443	Biolegend
CD8a	PerCP/Cy5.5	Mouse IgG1, κ	cytotoxic T cells	155014	Biolegend
CD19	PE-Cy7	Mouse IgG1, κ	mature B cells	159810	Biolegend
B220/CD45R	BV510	Rat IgG2b, κ	All stages B cells	563103	BD Biosciences
F4/80	BV711	Rat IgG2b, κ	macrophages	123147	Biolegend
CD11c	BV605	Armenian Hamster IgG	DC	117334	Biolegend
CD11b	APC	Rat IgG2b, κ	DC	101212	Biolegend
CD25	PE	Rat IgG2b, κ	Treg cells	101904	Biolegend
CD21/CD35	APC/Cy7	Rat IgG2b, κ	B1b cells	123418	Biolegend
CD5	BV650	Rat LOU/C, LOU/M IgG2a, κ	B1a cells	740444	BD Biosciences
PDCA-1 (tetherin)	PE-eFluor 610	Rat IgG2b, κ	pDC	61-3172-82	Thermo Fisher Scientific

References

1. Luo, Z., Solang, C., Mejia-Cordova, M., Thorvaldson, L., Blixt, M., Sandler, S., and Singh, K. (2019). Kinetics of immune cell responses in the multiple low-dose streptozotocin mouse model of type 1 diabetes. FASEB Bioadv 1, 538-549. 10.1096/fba.2019-00031.

2. Tonne, J.M., Sakuma, T., Deeds, M.C., Munoz-Gomez, M., Barry, M.A., Kudva, Y.C., and Ikeda, Y. (2013). Global gene expression profiling of pancreatic islets in mice during streptozotocin-induced beta-cell damage and pancreatic Glp-1 gene therapy. Dis Model Mech 6, 1236-1245. 10.1242/dmm.012591.

3. Goyal, S.N., Reddy, N.M., Patil, K.R., Nakhate, K.T., Ojha, S., Patil, C.R., and Agrawal, Y.O. (2016). Challenges and issues with streptozotocin-induced diabetes - A clinically relevant animal model to understand the diabetes pathogenesis and evaluate therapeutics. Chem Biol Interact 244, 49-63. 10.1016/j.cbi.2015.11.032.

4. Patra, M., Klochendler, A., Condiotti, R., Kaffe, B., Elgavish, S., Drawshy, Z., Avrahami, D., Narita, M., Hofree, M., Drier, Y., et al. (2024). Senescence of human pancreatic beta cells enhances functional maturation through chromatin reorganization and promotes interferon responsiveness. Nucleic Acids Res 52, 6298-6316. 10.1093/nar/gkae313.

5. Kim, Y.T., and Steinberg, C. (1984). Immunologic studies on the induction of diabetes in experimental animals. Cellular basis for the induction of diabetes by streptozotocin. Diabetes 33, 771-777. 10.2337/diab.33.8.771.

6. Lukic, M.L., Stosic-Grujicic, S., and Shahin, A. (1998). Effector mechanisms in low-dose streptozotocin-induced diabetes. Dev Immunol 6, 119-128. 10.1155/1998/92198.
