# Supplementary material for: Elevated MANF expression in β cells protects mice from streptozotocin-induced diabetes by attenuating islet stress and immunogenicity
Source: Mol Ther. 2026 Feb 27;34(6):3548–67. doi: 10.1016/j.ymthe.2026.02.043 (PMC13239798; doi:10.1016/j.ymthe.2026.02.043)
Supplement: Supplementary file 2 [file mmc4.pdf]

Molecular Therapy
Original Article

Elevated MANF expression in β cells protects mice from streptozotocin-induced diabetes by attenuating islet stress and immunogenicity

Huini Li,[1] Tatiana Danilova,[1] Erik Palm,[1] Emmi Pakarinen,[1] Julia Kiva,[1] Tõnis Org,[2,3] Tapani K. Koppinen,[4] Elina Hakonen,[5,6] Indrek Teino,[1] Merja H. Voutilainen,[4] Timo Otonkoski,[5,6] and Maria Lindahl[1]

[1]Institute of Biotechnology, HiLIFE, University of Helsinki, 00014 Helsinki, Finland; [2]Institute of Molecular and Cell Biology, University of Tartu, 51010 Tartu, Estonia; [3]Institute of Genomics, University of Tartu, 51010 Tartu, Estonia; [4]Division of Pharmacology and Pharmacotherapy, Faculty of Pharmacy, University of Helsinki, 00014 Helsinki, Finland; [5]Stem Cells and Metabolism Research Program, Faculty of Medicine, University of Helsinki, 00014 Helsinki, Finland; [6]Children's Hospital, University of Helsinki and Helsinki University Hospital, 00290 Helsinki, Finland

Type 1 diabetes (T1D) arises from autoimmune-mediated destruction of insulin-producing β cells, driven in part by endoplasmic reticulum (ER) stress and chronic unfolded protein response (UPR). We previously found that mesencephalic astrocyte-derived neurotrophic factor (MANF), an ER stress-regulating protein with protective and immunomodulatory roles, is essential for mouse and human β cell survival and proliferation. To assess the therapeutic potential of elevated endogenous MANF, we generated β cell-specific transgenic MANF-overexpressing mice and induced diabetes using multiple low-dose streptozotocin (MLDS) injections. In this study, we demonstrate that elevated MANF levels protected against MLDS-induced hyperglycemia, preserved β cell mass, enhanced proliferation, and reduced β cell DNA damage responses and islet lymphocyte infiltration. Transcriptomic profiling of MANF-overexpressing islets revealed downregulation of genes linked to ER and oxidative stress, inflammation, immune responses, antigen presentation, and p53-mediated senescence. Immunophenotyping further showed a reduction in CD4+ T cells in pancreatic lymph nodes. Mechanistically, elevated MANF suppressed MLDS-induced terminal UPR markers, including DNA damage inducible transcript 3 (*Ddit3*) and thioredoxin-interacting protein (TXNIP) expression, whereas MANF deficiency elevated their expression in β cells. Collectively, these findings identify MANF as a dual-acting therapeutic target that alleviates β cell stress and reduces immunogenicity in T1D.

INTRODUCTION

Type 1 diabetes (T1D) is an autoimmune disorder characterized by progressive destruction of pancreatic β cells due to islet infiltration by immune cells (insulitis), leading to absolute insulin deficiency. External insults such as viral infections, cytokines, and genetic predisposition contribute to chronic β cell endoplasmic reticulum (ER) stress, dysfunctional unfolded protein response (UPR), and eventual β cell death.[1,2] Emerging evidence suggests that β cells are not just passive participants during T1D pathogenesis. Their intrinsic vulnerability and high demand of insulin production and folding make them more susceptible to ER stress, provoking the autoimmune attack.[3,4] Therefore, novel therapeutic strategies emphasize the importance of reducing β cell stress in T1D in combination with immunotherapy.

Disruptions in ER homeostasis in β cells activate UPR ER transmembrane receptors, conserved inositol-requiring protein 1α (IRE1α), protein kinase RNA (PKR)-like ER kinase (PERK), and activating transcription factor 6 (ATF6).[5] Initially, activation of these receptors promotes an adaptive UPR to restore protein-folding homeostasis.[6] However, under irremediable ER stress, the UPR shifts to a chronic terminal UPR, leading to cell death.[7] Hyperactivated IRE1α engages adapter proteins, triggering apoptosis through the activation of pro-inflammatory genes in pancreatic β cells.[8] Furthermore, regulated IRE1α-dependent decay increases the stability of mRNA encoding thioredoxin-interacting protein (TXNIP), reducing the antioxidant response and activating the nucleotide-binding oligomerization domain-like receptor family pyrin domain-containing 3 (NLRP3) inflammasome and interleukin-1β (IL-1β) secretion.[9] Hyperactivated PERK phosphorylates eukaryotic translation initiation factor 2 subunit α (eIF2α) leading to translation arrest, sustained ATF4 translation, and increased expression of pro-apoptotic C/EBP homologous protein (CHOP), TXNIP, and nuclear factor kappa B (NF-κB), contributing to the autoimmune response and β cell death in T1D.[5,10] Sustained ER stress has been implicated to modulate the immunopeptidome and immunogenicity of β cells.[2,3,11–14] In contrast, mild ER stress increases β cell proliferation by reducing tribbles pseudokinase 3 (TRIB3), a negative regulator of protein kinase B (AKT).[15]

Received 29 September 2025; accepted 24 February 2026;
https://doi.org/10.1016/j.ymthe.2026.02.043.

Correspondence: Maria Lindahl, Institute of Biotechnology, HiLIFE, University of Helsinki, Viikinkaari 5D, 00014 Helsinki, Finland.
E-mail: maria.lindahl@helsinki.fi

 © 2026 The Author(s).
Published by Elsevier Inc. on behalf of The American Society of Gene and Cell Therapy.
This is an open access article under the CC BY license (http://creativecommons.org/licenses/by/4.0/).

Therefore, means of reducing ER stress leading to endogenous β cell protection and regeneration are currently under intensive investigation as therapeutic approaches for diabetes.[3]

Mesencephalic astrocyte-derived neurotrophic factor (MANF) is a small 18 kDa ER-localized protein, highly expressed in metabolically active tissues, that can be secreted from cells in stress.[16–19] Recombinant MANF protein protects and restores damaged cells in various animal models of neurodegenerative, inflammatory, and metabolic diseases.[20] Although its mechanism of action is still unclear, it binds to lipid sulfatides in cell membranes and is suggested to be endocytosed and transported back to the ER.[21] Additionally, it binds the neuroplastin receptor, alleviates NF-κB-regulated inflammatory responses and promotes insulin secretion in β cells.[22] MANF engages UPR regulating ER chaperone protein BiP (GRP78/BiP) to increase protein folding.[23,24] It also binds all three main UPR receptors and reduces IRE1α activation, oligomerization, and downstream signaling.[25] Thus, the quantity of MANF may be critical for regulating cellular ER stress levels.

We have previously shown that MANF is needed for the postnatal survival and expansion of pancreatic β cells in mice, as loss of MANF resulted in diabetes due to reduced β cell proliferation and increased β cell death caused by chronic ER stress.[18,26] Importantly, loss-of-function mutations in the *MANF* gene in humans cause childhood-onset syndromic diabetes due to increased β cell ER stress, and impaired insulin production and secretion.[27] Recombinant MANF protein induced proliferation of human and mouse β cells and protected against chemical- and pro-inflammatory cytokine-induced ER stress and cell death *in vitro*.[17,18,26,28] In addition, exogenous MANF reduced NF-κB activation and its target genes in cytokine-treated human primary β cells.[17] In our gene therapy experiment, increased MANF in the pancreas, delivered by adeno-associated virus vector, was found to partially protect β cells in a multiple low-dose streptozotocin (MLDS) model. However, due to low transduction efficiency, no therapeutic effect was detected.[18] For this study, we developed an inducible bi-transgenic mouse model (INS-MANF) overexpressing MANF specifically in β cells. Streptozotocin (STZ) is a toxic glucose analog that enters β cells through glucose transporter 2 (GLUT2), causing DNA damage, oxidative stress, ER stress, and β cell death.[29,30] It is a well-established model for studying β cell-intrinsic stress responses that precede autoimmunity in T1D and for evaluating preclinical therapeutic strategies in rodents.[30] We induced diabetes in INS-MANF and control mice using both the single high-dose STZ (SHDS) model, causing acute β cell cytotoxicity and death,[31] and the milder MLDS model mimicking immune-mediated T1D through progressive β cell stress and subsequent islet immune cell infiltration.[32]

Together, our results show that elevated MANF efficiently protects against MLDS-induced β cell death, insulitis, and hyperglycemia. RNA sequencing (RNA-seq) of islets from prediabetic mice revealed that the protective effect of MANF was associated with reduced ER and oxidative stress, DNA double-strand break (DSB) repair, p53-mediated senescence, and immune cell recruitment and activation. Importantly, these changes correlated with enhanced β cell survival and regeneration, suggesting that elevating MANF levels in β cells simultaneously resolves β cell stress and reduces immunogenicity in T1D.

RESULTS

Elevated MANF does not affect β cell mass or β cell function

To test the therapeutic effect of local overexpression of human MANF in β cells of STZ-treated diabetic mice, we generated a MANF transgenic mouse line (TET-MANF) utilizing the TET-On system. By crossing this line with an insulin promoter-dependent reverse tetracycline transactivator (INS-rtTA)-expressing mouse line, human MANF expression can be specifically induced in β cells by doxycycline (DOX) administration (Figure 1A). First, the bi-transgenic mice were examined in more detail. We confirmed a significant and specific overexpression of the human transgenic MANF in insulin-positive β cells of bi-transgenic INS-MANF mice on a DOX+ diet compared with INS-MANF DOX– control mice (Figures S1A–S1G). Immunostaining of pancreas sections confirmed that transgenic MANF expression was specific to β cells but not in α cells (Figures S1G and S1H). A small increase in MANF expression was seen in islets from a few bi-transgenic INS-MANF DOX– mice (not shown), indicating minor leakiness of the TET-On system, as previously reported.[33] Continuous MANF overexpression in β cells for 3 months did not lead to detectable systemic or local islet adverse effects, including body weight change, glycemic status, serum insulin levels, β cell function, β cell mass, proliferation or survival, or the expression of β cell-specific genes including *Glut2* (Figures 1B–1K). In some transgenic lines utilizing the insulin 2 promoter, ectopic transgene expression has been found induced in nutrient-sensing neurons of the hypothalamus.[34] However, we confirmed no change in transgenic human MANF expression in the hypothalami of INS-MANF DOX+ mice (Figures S1I and S1J). We further tested the long-term effects of β cell MANF overexpression for 5 months (Figures S2A–S2F) and 11 months (Figure S2G) in male mice and found no β cell hypertrophy or islet morphological or pathological changes *in vivo*.

Transgenic MANF protects against single high-dose STZ-induced diabetes

We then tested whether increased transgenic MANF in β cells protects against SHDS-induced diabetes. Male INS-MANF and control single-transgenic (sTG) mice were fed with a DOX+ diet for 3 months, whereas control INS-MANF mice received normal food (DOX–) prior to SHDS injection (Figure 2A). SHDS resulted in loss of β cells and hyperglycemia in both control single- and bi-transgenic STZ-injected mice within 2 days (Figure 2B). In contrast, MANF-overexpressing mice (INS-MANF DOX+) showed significantly lower blood glucose levels throughout the experiment, which were not significantly higher than the control sTG DOX+ mice not injected with STZ (Figure 2B). Due to hyperglycemia, control STZ-injected mice lost a significant amount of weight (20%–22%) and all mice were sacrificed at day 9 after the STZ injection (Figure 2C). As expected, the weight loss of MANF-overexpressing mice was significantly smaller (13%) compared

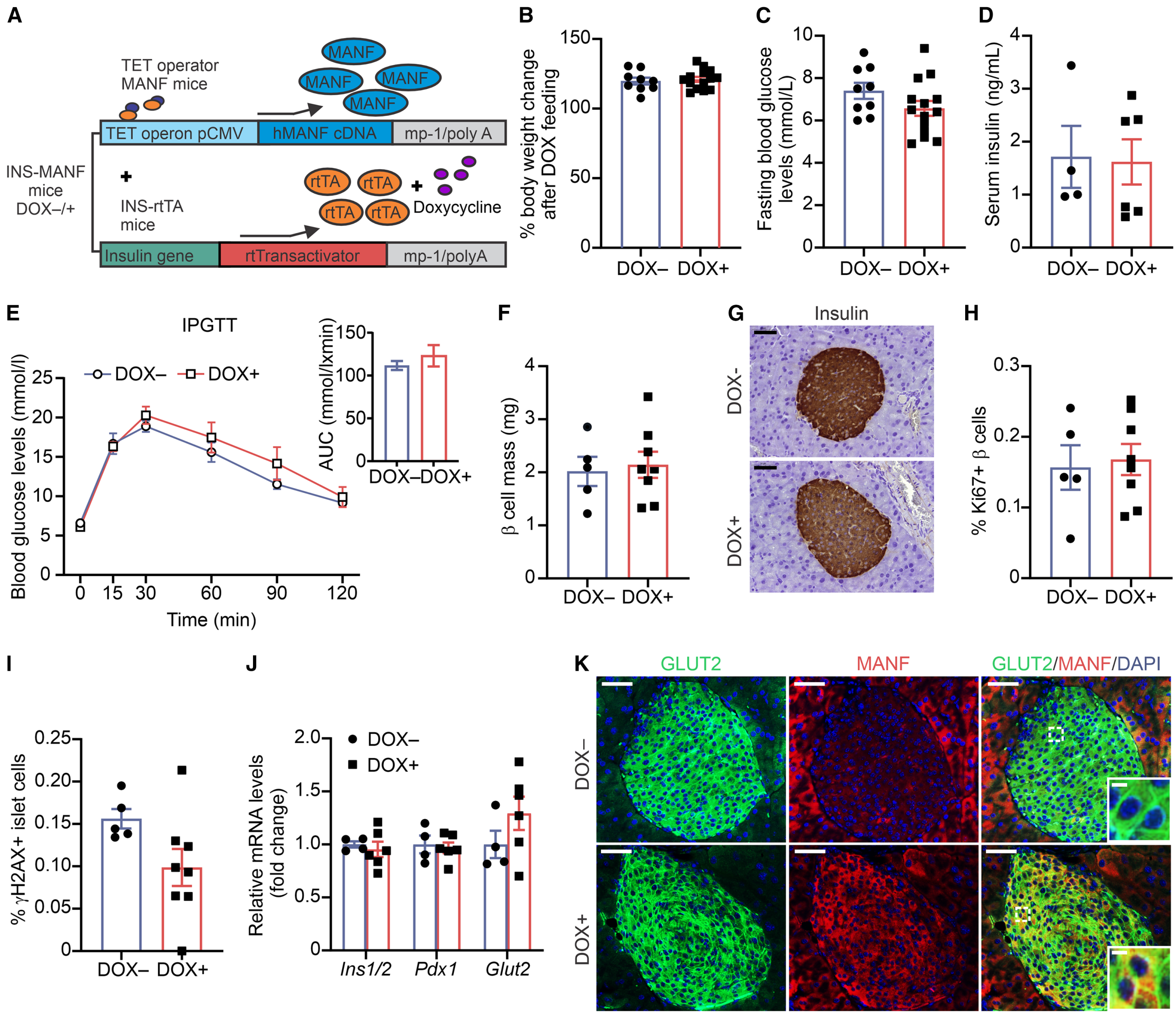

Figure 1. Characterization of male bi-transgenic INS-MANF mice on doxycycline chow for 3 months

(A) Schematic illustration of the transgenic constructs for INS-rtTA and TET-MANF single-transgenic mice used for generating bi-transgenic INS-MANF mice. (B) Body weight gain (%) in MANF-overexpressing (INS-MANF DOX+, $n = 14$) compared with INS-MANF DOX– ($n = 9$) mice 3 months after DOX+ or normal chow feeding. (C) Blood glucose levels from fasted mice (DOX+, $n = 14$; DOX–, $n = 9$). (D) *Ad libitum* serum insulin levels (DOX+, $n = 6$; DOX–, $n = 4$), and (E) intraperitoneal glucose tolerance test (IPGTT) and area under the curve (AUC) of the mean blood glucose levels for each group (DOX+, $n = 14$; DOX–, $n = 9$). (F and G) β cell mass quantified from insulin antibody-stained pancreas sections of INS-MANF DOX+ ($n = 8$) and DOX– ($n = 5$) mice. Scale bars, 50 μm (G). (H) Relative number of proliferating β cells quantified from pancreas sections stained with Ki67 and insulin antibodies (DOX+, $n = 8$; DOX–, $n = 5$). (I) Relative number of γH2AX-positive cells in islets (DOX+, $n = 8$; DOX–, $n = 5$). (J) RT-qPCR analysis of β cell identity genes in islets (DOX+, $n = 6$; DOX–, $n = 4$). (K) Membrane localization of GLUT2 in pancreas sections stained with anti-MANF (red) and anti-GLUT2 (green) antibodies. Nuclei were stained by DAPI (blue). Scale bars, 50 and 5 μm (insets). Data are expressed as mean ± SEM, and scatterplots show all individual data points. Statistical analyses were performed using Student's two-tailed unpaired *t* test for (B–D), AUC in (E), (F), and (H–J) and two-way ANOVA followed by Bonferroni's post hoc test for the different time points in (E).

with the control STZ mice. Immunohistochemical analysis of pancreas sections revealed conserved islet architecture in the MANF-overexpressing mice compared with STZ-treated control mice (Figure 2D). Quantification of the β cell mass revealed preserved β cell mass in the STZ-treated MANF-overexpressing pancreases compared with the STZ-treated control mice (Figure 2E). Interestingly, increased β cell proliferation was found in islets from MANF-overexpressing STZ-treated mice compared with control mice (Figure 2F). DNA damage response (DDR) is activated in β cells of T1D patients.[35] It is also induced by STZ in mice, which leads to DNA DSBs, islet inflammation,

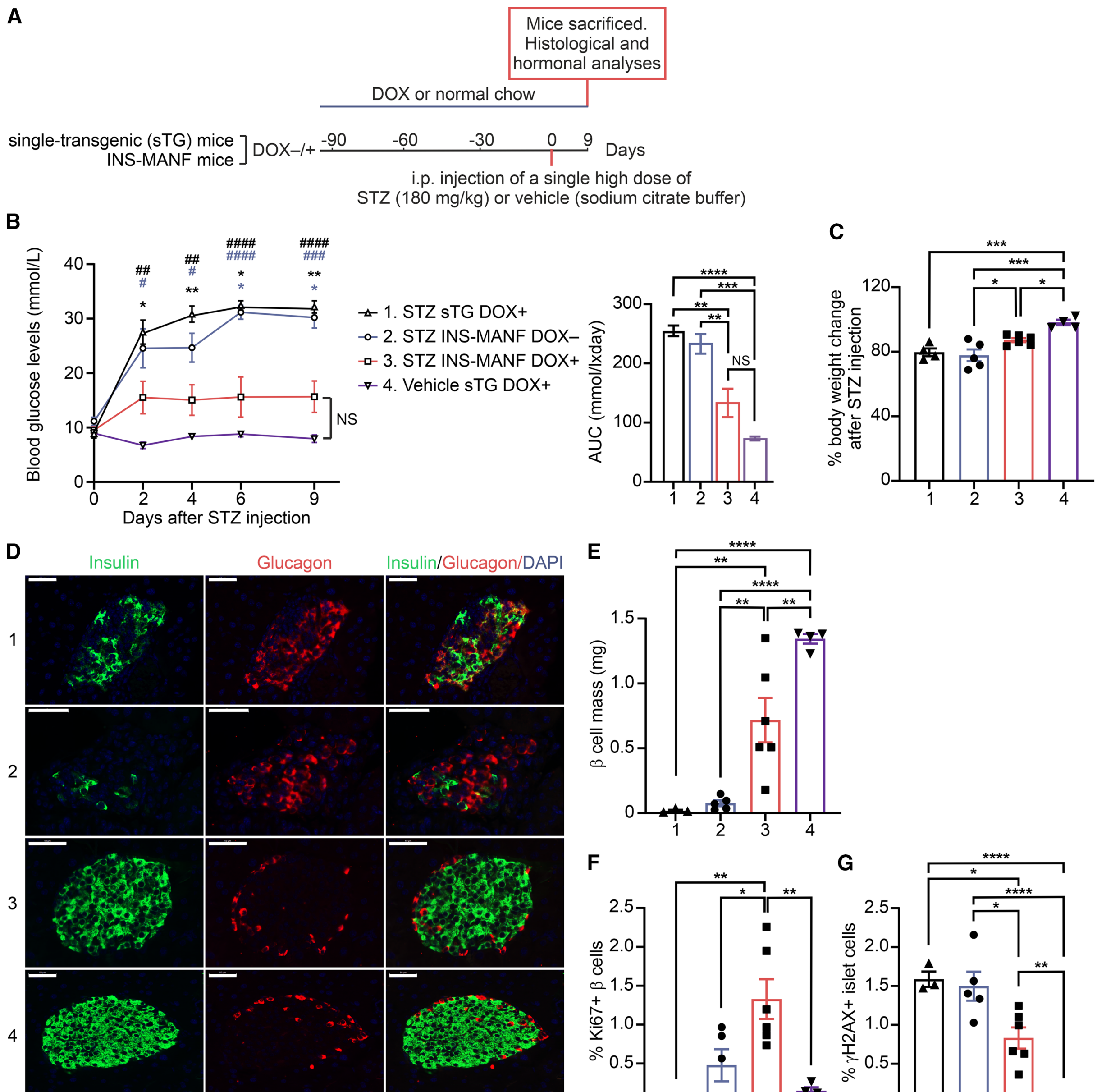

Figure 2. Elevated MANF expression in β cells protects from a single high dose of streptozotocin-induced diabetes

(A) Timeline for DOX or normal chow feeding, followed by a single high dose of STZ injection (180 mg/kg). Non-diabetic controls (INS-rtTA DOX+) received equivalent 25 mM sodium citrate buffer (pH 4.5). (B) *Ad libitum* blood glucose levels measured before STZ injection and at four time points up to 9 days after injection and area under the curve (AUC) of the mean blood glucose levels for each group: (1) STZ TET-MANF sTG DOX+ ($n = 4$), triangles, black line; (2) STZ INS-MANF DOX– ($n = 5$), circles, blue line; (3) STZ INS-MANF DOX+ ($n = 6$), squares, red line; (4) vehicle INS-rtTA sTG DOX+ ($n = 4$), triangles, purple line. Statistical significance is indicated as follows: black * for group 3 vs. group 1; blue * for group 3 vs. group 2; black # for group 4 vs. group 1; and blue # for group 4 vs. group 2. No significant difference (NS) was observed between group 3 and group 4. (C) Body weight change (%) 9 days after STZ or vehicle injection. (D) Anti-insulin and anti-glucagon antibody-stained pancreatic sections from STZ-treated and control mice. Scale bars, 50 μm. (E) β cell mass calculated from insulin-stained pancreas sections. (F) β cell proliferation calculated from Ki67- and insulin-stained pancreas sections. (G) Number of γH2AX-positive cells compared with total islets cells. Data are expressed as mean ± SEM, and scatterplots show all individual data points. Statistical analyses were performed using two-way ANOVA followed by Tukey's post hoc test for the different time points in (B), and one-way ANOVA followed by Tukey's post hoc test for AUC in (B), (C), and (E–G). *$p < 0.05$, **$p < 0.01$, ***$p < 0.001$, ****$p < 0.0001$, #$p < 0.05$, ##$p < 0.01$, ###$p < 0.001$, ####$p < 0.0001$, NS > 0.05.

and β cell death.[35] Phosphorylation of histone variant H2AX at serine 139 (γH2AX) is a widely used marker for DSBs, DDR, senescence, and apoptotic DNA fragmentation.[36] We found less-frequent γH2AX-positive islet cells in STZ-treated MANF-overexpressing pancreases compared with controls (Figure 2G). Importantly, DOX feeding alone did not prevent the STZ-induced β cell death and diabetes in control sTG DOX+ mice (Figures 2B–2G).

Elevated MANF in β cells protects against multiple low doses of STZ-induced diabetes

Next, we studied whether elevated MANF levels in β cells confer protection against MLDS-induced T1D. As previously, INS-MANF mice and control sTG mice were pre-fed with a DOX diet, whereas control INS-MANF mice received normal food (Figure 3A). As expected, the development of hyperglycemia in this model was slower in control mice compared with the SHDS model. Mice were sacrificed when control mice reached severe hyperglycemia 21 days after the first STZ injection (Figure 3B). Blood glucose levels in MANF-overexpressing mice increased marginally but remained low compared with STZ-injected control mice throughout the experiment (Figure 3B). The weight loss of MANF-overexpressing mice was significantly smaller (5.4%) than in the STZ-injected control sTG mice (16%) (Figure 3C). Consequently, endpoint serum insulin levels in fed state animals were significantly higher in MANF-overexpressing mice compared with control mice (Figure 3D). Similarly, the β cell mass was higher in MANF-overexpressing mice compared with control STZ-treated mice (Figure 3E), whereas no difference in α cell mass was detected (Figure 3F). The islet architecture was relatively intact in MANF-overexpressing pancreases (Figure 3G). This result was in line with the increased β cell proliferation (Figure 3H) and reduced β cell DSBs (Figure 3I) in the MANF-overexpressing islets compared with STZ-treated controls, suggesting a protective but also a regenerative capacity for elevated MANF after insult.

A separate set of MANF-overexpressing mice were treated with MLDS and followed for 4.5 months to analyze whether β cell-specific MANF overexpression normalizes blood glucose levels after MLDS injections with time (Figure S3A). Control mice needed to be sacrificed earlier when reaching hyperglycemia (Figure S3B). MANF-overexpressing mice had a transient episode of slightly elevated blood glucose levels but returned to normoglycemia with normal serum insulin levels at 4.5 months after the first STZ injection (Figures S3B and S3C). A relatively higher β cell mass with a trend for increased β cell proliferation and reduced β cell death was detected in the pancreases of MANF-overexpressing mice, indicating sustained β cell protection over time compared with control mice at 45 days after the first STZ injection (Figures S3D–S3F).

MANF-induced β cell protection is associated with reduced islet insulitis, terminal UPR, and CD4+ T cells in pancreatic lymph nodes

Immunostaining revealed that MLDS-injected control mice developed insulitis with destroyed islet architecture infiltrated by CD45+ lymphocytes and a dramatic loss of pancreatic β cells, whereas MLDS-injected MANF-overexpressing mice had more intact islets and β cells (Figure 4A), similarly to MANF-overexpressing mice maintained for 4.5 months after the first STZ injection (Figure 4B). The severity of insulitis (Figure S3G) was measured 3 weeks after the first STZ injection in the MLDS model from hematoxylin and eosin-stained pancreas sections (Figure 4C). Results from scoring revealed that 57.7% ± 4% of the islets in the MANF-overexpressing mice were intact with no insulitis (score 1, Figure 4C), compared with 9.2% ± 4% of the control sTG mice and 17.3% ± 4% of the INS-MANF DOX− mice. In addition, islets from MANF-overexpressing mice rarely showed major insulitis (score 5, Figure 4C). Insulitis assessment of islets from MANF-overexpressing mice, 4.5 months after the first MLDS injection, confirmed that elevated MANF in β cells effectively protects against lymphocyte islet infiltration in the MLDS model (Figure S3H). TXNIP, a key mediator of oxidative stress and inflammasome activation in β cells, is upregulated in terminal UPR driving both β cell pro-inflammatory signaling and apoptosis.[37,38] In contrast, loss of TXNIP in mice leads to increased β cell mass and resistance to STZ-induced diabetes.[39] Co-staining of pancreatic sections from control mice 21 days after the first STZ injection revealed increased expression of TXNIP and IL-1β in insulin-positive β cells, whereas TXNIP and IL-1β staining were weak in MANF-overexpressing islets (Figures 4D and S4).

To further investigate the early effects of elevated β cell-specific MANF on the immune response in the MLDS model, immunophenotyping by flow cytometry was performed. Immune cell populations in the pancreatic lymph nodes (pLNs) and spleens of vehicle-treated non-diabetic control, STZ-treated MANF-overexpressing, and INS-MANF DOX− mice were quantified at days 4 and 10 after the first MLDS injection according to a previous study.[40] A panel of 13 antibodies was used to profile most of the immune cell types described to contribute to the immunopathology in T1D (gating strategy, Figure S5).[41] Islet antigens carried by antigen-presenting cells from inflamed islets to the pLNs are critical contributors to the pathogenesis of T1D by initiating proliferation and priming of autoreactive T cells.[42] As expected, most immune cell populations showed a trend toward increased number in the pLNs at day 4 after STZ treatment. Notably, the number of CD4+ T helper cells was significantly increased after STZ treatment (Figure 4E). Conversely, β cell-specific MANF overexpression significantly reduced the STZ-induced increase in both CD3+ T cells and CD4+ T helper cells in the pLNs (Figure 4E). The number of B cells, CD8+ T cells, macrophages, and pan-dendritic cells showed a similar trend in the pLNs (Figure 4E). However, on day 10 after the STZ treatment no significant alterations in different immune cells in the pLNs were detected (Figure 4F). In addition, elevated β cell-specific MANF expression did not affect immune cell populations in the spleen, except for an increase in the number of CD11c+ CD8+ dendritic cells at day 4 (Figures S6A and S6B).

β cell-specific MANF lowers STZ-induced islet expression of immune, oxidative stress, and p53-mediated senescence genes in prediabetic mice

To further investigate the molecular mechanisms of MANF-induced β cell protection against STZ, we performed bulk RNA-seq following

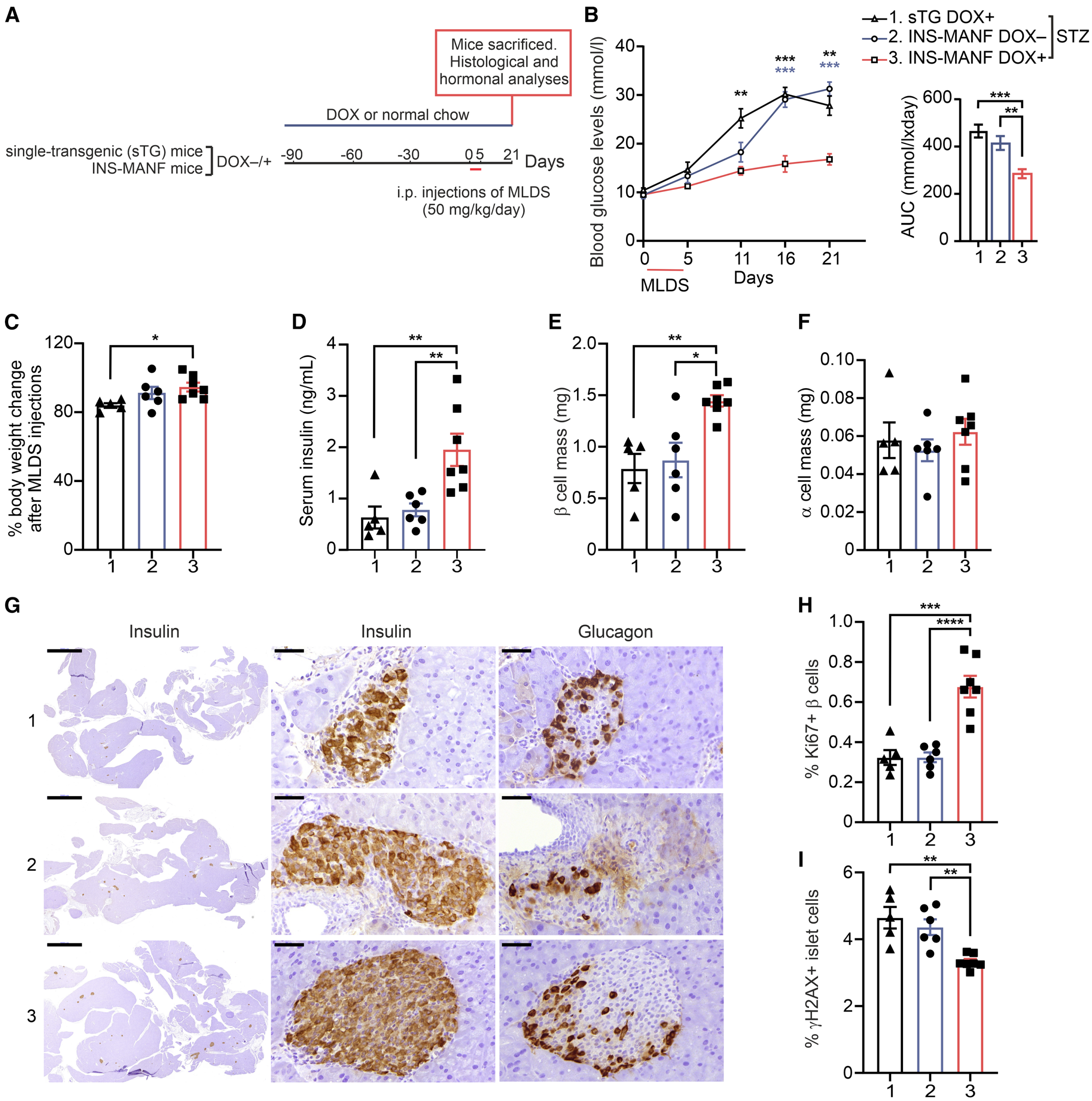

Figure 3. Elevated β cell-specific MANF expression protects from multiple low doses of STZ-induced hyperglycemia, β cell loss, and induces β cell regeneration

(A) Timeline for DOX+ and normal diet feeding, followed by MLDS injections (50 mg/kg/day for 5 consecutive days). (B) Measurements of blood glucose levels of *ad libitum* fed animals and area under the curve (AUC) of the mean blood glucose levels for each group: (1) STZ sTG DOX+ mice including TET-MANF DOX+ (n = 3) and INS-rtTA DOX+ mice (n = 2), triangles, black line; (2) STZ INS-MANF DOX– mice (n = 6), circles, blue line; and (3) STZ INS-MANF DOX+ mice (n = 7), squares, red line. Statistical significance is indicated as follows: black * for group 3 vs. group 1; and blue * for group 3 vs. group 2. (C) Body weight change (%) 21 days after the first STZ injection. (D) *Ad libitum* serum insulin levels from terminal blood. (E) β cell mass quantified from insulin-stained pancreas sections of mice sacrificed 21 days after the first STZ injection. (F) Quantification of α cell mass from glucagon-stained sections. (G) Images from insulin and glucagon antibody-stained pancreatic sections of STZ-treated mice. Scale bars, 2,000, 50, and 50 μm (from left to right). (H) β cell proliferation quantified from insulin and Ki67 antibody-stained sections in STZ-treated mice. (I) β cell DDR assessed by anti-γH2AX antibody staining in islets from STZ-injected mice. Data are expressed as mean ± SEM, and scatterplots show all individual data points. Statistical analyses were performed using two-way ANOVA with Tukey's post hoc test for the different time points in (B) and one-way ANOVA followed by Tukey's post hoc test for AUC in (B), (C–F), and (H–I). *$p < 0.05$, **$p < 0.01$, ***$p < 0.001$, ****$p < 0.0001$.

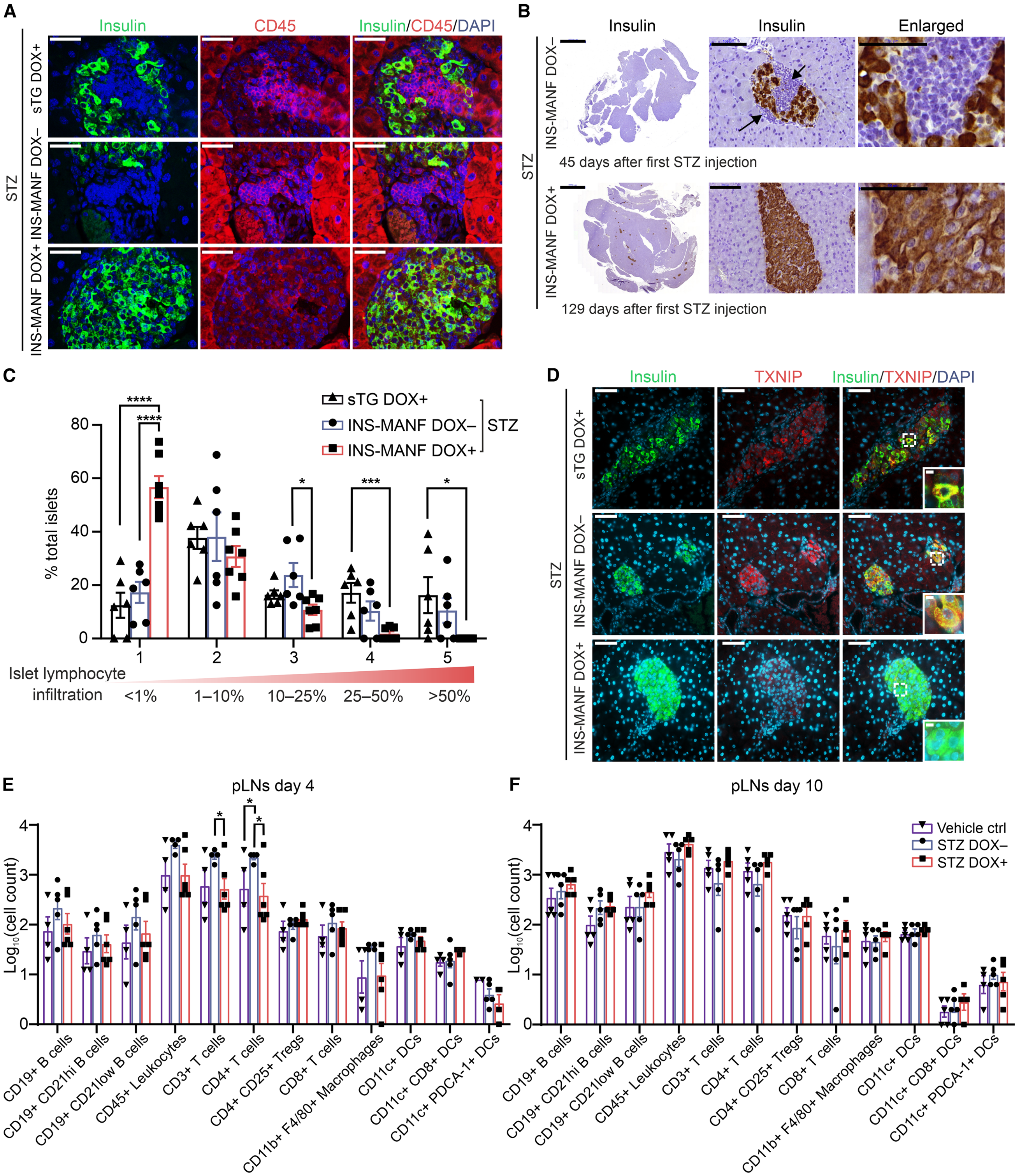

Figure 4. Elevated MANF expression in β cells efficiently protects against islet insulitis, TXNIP upregulation and reduces the number of CD4+ T cells in pancreatic lymph nodes

(A) Representative pictures of CD45- and insulin-stained pancreatic sections from MLDS-injected mice 21 days after the first STZ injection. Scale bars, 50 μm. (B) Representative pictures of insulin- and hematoxylin-counterstained pancreas with islet lymphocyte infiltration (arrows) in MLDS-injected DOX– compared with DOX+

(legend continued on next page)

poly(A) selection of mRNA on islets isolated from the pancreases of vehicle- and STZ-injected control mice (INS-MANF DOX−, sTG DOX+, and sTG DOX−) and MANF-overexpressing mice (INS-MANF DOX+) on the 4th day after 3 consecutive days of low doses of STZ or vehicle injections. We confirmed that transgenic human *MANF* was expressed in all replicates isolated from each INS-MANF DOX+ mouse by reverse-transcription quantitative PCR (RT-qPCR) (Figure S7A). Using principal-component analysis, we demonstrated that DOX treatment itself did not induce major global changes in gene expression compared with the strong effect of STZ (Figure S7B).

The transcriptional effect of STZ alone was first compared between islets from vehicle- and STZ-treated INS-MANF DOX− control mice. We identified 643 differentially expressed genes (DEGs) with Log2 fold change ($|Log_2FC|$) > 0.5 and FDR < 0.05 (Table S1), including 322 upregulated and 321 downregulated genes in response to STZ. Gene Ontology (GO) enrichment analyses of 322 upregulated DEGs by STZ using the DAVID functional annotation tool showed that the top enriched terms describing biological processes (BPs) were involved in inflammatory and innate immune responses, chemokine-mediated signaling, tumor necrosis factor production, ROS metabolic processes, DDRs, and intrinsic apoptotic signaling in response to DNA damage by the p53 class mediator (Figure 5A; Table S1). Among the 321 downregulated genes by STZ, the top BP terms involved locomotor behavior, positive regulation of gene expression, cell adhesion, extracellular matrix organization, intracellular calcium ion homeostasis, and monoatomic cation transmembrane transport (Figure 5B; Table S1). Results on GO-BP terms and KEGG enrichment pathway analyses are shown in more detail in Figures S7C–S7F and Table S1. A heatmap was generated to highlight the relative average expression levels of key islet genes affected by STZ alone, selected from the 643 DEGs (Figure 5C). STZ treatment significantly reduced the expression of genes involved in insulin production, secretion, and glucose sensing, indicating early β cell dysfunction. Meanwhile, genes essential for β cell identity and progenitor markers remained unchanged (Figure 5C). Thus, the time point set for RNA-seq in our prediabetic MLDS model detects stressed β cells without complete loss of β cell identity, but alongside immune responses associated with markers for senescence and apoptosis (supplemental information).[40,43,44]

When comparing the DEGs altered by STZ alone to genes affected by MANF overexpression ($|Log_2FC| > 0.25$, $p < 0.05$), we found 21 genes increased by STZ but decreased by MANF overexpression in the presence of STZ (Figure 5D; Table S1). The genes upregulated by STZ but downregulated by MANF were mostly related to the regulation of immune cell activation and recruitment, cytokine and chemokine production, as well as immune cell adhesion and migration, which are essential processes modulating immune cell interaction with β cells (Figures 5D and 5E). This aligns with our observations of reduced islet infiltration of immune cells and decreased numbers of CD4+ T cells in pLNs of MLDS-injected MANF-overexpressing mice (Figure 4E). In addition, reduced expression of *Hmox1* and *Steap1*, two oxidative response genes, was found in the MANF-overexpressing islets, suggesting a lowered oxidative burden and diminished activation of cellular antioxidant defenses (Figures 5D and 5F). The expression of *Ccng1*, encoding cyclin G1, and *Trp53inp1*, encoding tumor protein 53-inducible nuclear protein 1, both key p53 transcriptional targets involved in G1 cell-cycle arrest and senescence in response to cellular stress, was also downregulated by elevated MANF expression (Figures 5D and 5G). To further investigate whether MANF could reduce targets for cell-cycle arrest and senescence by alleviating ER stress, we treated primary mouse islets with ER stressor thapsigargin (TG), an inhibitor of the sarco/endoplasmic reticulum Ca^{2+}-ATPase (SERCA) pump, alone or in combination with recombinant human MANF (rhMANF) protein. In addition to reducing UPR markers (Figures 5H–5J), MANF also attenuated TG-induced expression of the senescence marker *Cdkn1a* (p21) and showed a trend toward reduced *Trp53* (p53) expression (Figure 5K and 5L) as well as *Trib3*, an AKT inhibitor (Figure 5M).

RNA-seq revealed that many of the genes downregulated by STZ but upregulated by MANF were involved in cell adhesion (*Cntnap2*, *Sdk1*, *Cdhr18*, *Nrxn1*, *Cntn1)*, synaptic function and neurotransmission (*Trim9*, *Sntg1*, *Cntn1*, *Nrxn1*), neuronal plasticity (*Foxp2*, *Npas2*, *Kalrn*, *Cntnap2*, *Sh3pxd2a*), Ca^{2+} signaling (*Trpm3*, *Gucy2c*, *Slc9a9*, *Slc15a2*), and transcriptional regulation and gene expression (*Foxp2*, *Hdac9*, *Mir670hg*) (Figure 5D). The increased expression of neuronal genes is not surprising as the exocytic system involving pulsatile hormonal secretion in islets is very similar to those of neuronal signaling.[45] MANF, as a neurotrophic factor, may also maintain neuronal innervation and survival, and thereby preserve islet endocrine function.[46]

Elevated β cell-specific MANF reduces STZ-induced UPR, antigen presentation, and autoantigen genes

To study the effect of MANF overexpression upon STZ treatment, we compared DEGs solely between STZ-injected INS-MANF

islets. Scale bars, 2 mm, 100 μm, and 50 μm (left to right). (C) Islet insulitis 21 days after the first STZ injection was scored and calculated as percent of islets per mouse at each stage of insulitis, from hematoxylin and eosin-stained pancreas sections, using severity scaling 1–5. Treatment groups include STZ-treated sTG DOX+ mice (STZ sTG DOX+, *n* = 5), triangles, black bar; STZ INS-MANF DOX− (*n* = 6), circles, blue bar; and STZ INS-MANF DOX+ (*n* = 7), squares, red bar. (D) Representative pictures of TXNIP (red) co-stained with insulin (green) in pancreatic sections from MLDS-injected INS-MANF and control mice 21 days after first STZ injection. Scale bars, 50 and 10 μm (insets). (E and F) Immune cell profiling in pLNs on day 4 (E) and day 10 (F) after the first STZ (45 mg/kg/day) or vehicle injections. Treatment groups include non-diabetic control mice injected with vehicle (Vehicle ctrl, *n* = 4), triangles, purple bar including TET-MANF DOX− (*n* = 1), INS-rtTA DOX− (*n* = 2), and INS-MANF DOX− (*n* = 1), INS-MANF DOX− mice injected with STZ (STZ DOX−, *n* = 5), circles, blue bar, and INS-MANF DOX+ mice injected with STZ (STZ DOX+, *n* = 6), squares, red bar. Cell counts were log10-transformed prior to analysis. Data are expressed as mean ± SEM, and scatterplots show all individual data points. Statistical analyses were performed using one-way ANOVA with Tukey's post hoc test for the different scores in (C) and two-way ANOVA followed by Holm-Šidák's multiple comparison test for (E) and (F). *$p < 0.05$, ***$p < 0.001$, ****$p < 0.0001$.

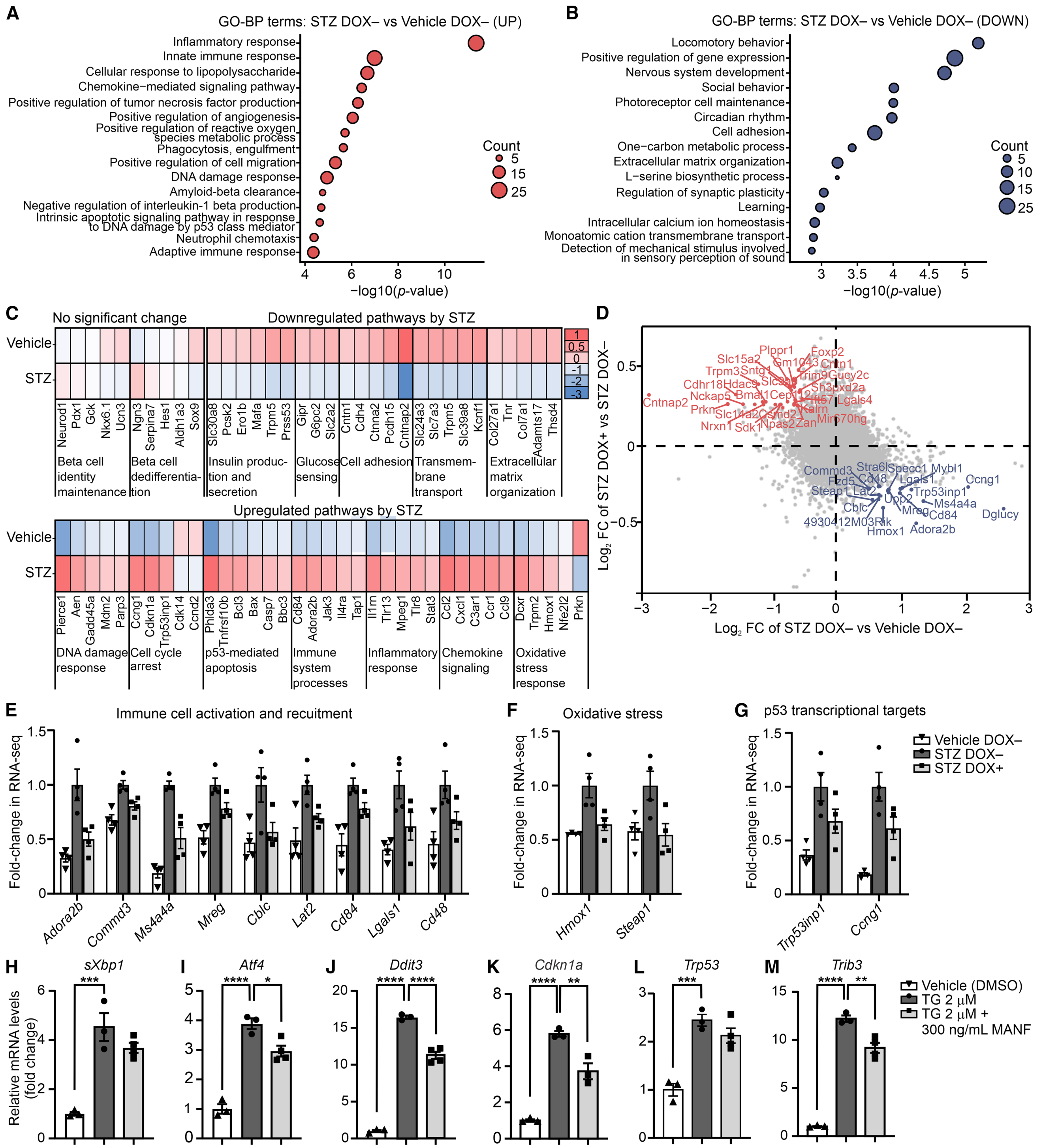

Figure 5. Reduced expression of genes associated with immune cell activation and recruitment, oxidative stress, and p53 transcriptional targets in islets from STZ-injected INS-MANF DOX+ mice

(A and B) Bubble diagrams of the GO enrichment analyses for biological processes (BP) terms by DAVID, on islet mRNA using 322 significantly upregulated DEGs (A) and 321 significantly downregulated DEGs (B) with $|Log2(FC)| > 0.5$ and $FDR < 0.05$ showing the effect of STZ in INS-MANF DOX– islets (STZ DOX–, $n = 4$) compared with vehicle-treated mice (Vehicle DOX–, $n = 4$). (C) Heatmap and relative average expression of genes and pathways differentially regulated by STZ compared with vehicle treatment in islets from INS-MANF DOX– control mice selected from the 643 DEGs ($|Log_2(FC)| > 0.5$, $FDR < 0.05$, $n = 4$). (D) DEGs in islets between STZ and vehicle-injected INS-MANF

(legend continued on next page)

DOX+ and DOX− mice. DEG analyses with DESeq2 identified 64 significantly downregulated genes and 75 upregulated genes by MANF (FDR < 0.05) shown in a volcano plot (Figure 6A; Table S2). Using the DAVID tool, we further performed GO enrichment analyses separately for the downregulated and upregulated genes affected by elevated MANF between the STZ-injected INS-MANF DOX± groups (FDR < 0.05) (Figures 6B and 6C; Table S2). Interestingly, the top enriched terms describing BP using the downregulated genes were mainly involved in ER stress response, UPR, protein UFMylation, reticulophagy, and ER to Golgi vesicle-mediated transport (Figure 6B). Importantly, the pro-apoptotic UPR genes, *Ddit3* (CHOP) and *Nupr1*, were significantly downregulated by MANF overexpression upon STZ (Figures 6A and 6D). In addition, ER stress-related genes encoding proteins involved in UFMylation (*Ufm1*, *Uba5*), reticulophagy (*Crebrf*, *Hspa13)*, ER-associated degradation (*Derl1*, *Serp1*), and ER translocation (*Tram1*) were significantly downregulated by MANF with FDR < 0.05 (Figure 6D). Additional genes associated with ER translocation and translational arrest (*Eif4ebp1*), protein folding (*Dnajb9*), and ER stress-responsive genes (*Stc2*, *Chac1*) were also downregulated ($p < 0.05$). Moreover, *Atf5*, encoding for a regulator of TXNIP expression via the PERK-eIF2α signaling pathway during ER stress, was similarly reduced.[47] In addition, genes regulating innate immune response (*Pparg*, *Adora2b*, and *Ang*, FDR < 0.05) were downregulated by MANF (Figure 6E). Interestingly, a significant decrease in the expression of *H2-Ea* (histocompatibility 2, class II antigen E α, FDR < 0.05), encoding for a member of the major histocompatibility complex (MHC) class II genes and involved in antigen presentation to CD4+ T cells,[48] was found in islets from STZ-injected MANF-overexpressing mice (Figure 6F). In addition, the expression of *Iapp* (islet amyloid peptide, FDR < 0.05) and *G6pc2* (islet-specific glucose-6-phosphatase catalytic subunit-related protein, $p < 0.05$), encoding for autoantigens recognized by autoreactive T cells in both human T1D patients and non-obese diabetic (NOD) mice,[49,50] was reduced by MANF upon STZ (Figure 6F).

Top enriched BP terms in the group of 75 significantly upregulated genes by MANF were involved in cell adhesion and synaptic function (Figures 6C and 6G), as well as small GTPase-mediated and Ras protein signal transduction, related to cell survival, differentiation, adhesion, migration, and proliferation (Figures 6C and 6H). *Gli2*, a Hedgehog (Hh) signaling mediator involved in β cell differentiation and proliferation,[51] was among the top 5 upregulated genes by MANF (Figures 6A and 6H). *Rit2* and *Rapgef4*,[52] regulators of RAS protein signal transduction associated with cell survival and proliferation, were also upregulated by MANF (Figures 6A and 6H).

Elevated β cell-specific MANF protects islets *in vitro* against STZ-induced ER stress, TXNIP upregulation, and inflammation

We validated our mRNA-seq data by RT-qPCR analyses of islets from a new set of STZ-injected mice using the same *in vivo* protocol as above. We confirmed that increased MANF in islets led to a reduced expression of *Cd48* and *Cd84* compared with islets from STZ-injected control mice (Figure 7A). In addition, there was a reduced expression of p53 targets, *Ccng1* and a trend toward reduced expression of *Trp53inp1* in MANF-overexpressing islets (Figure 7B). Elevated MANF in β cells also significantly attenuated STZ-induced upregulation of *H2-Aa*, a component of MHC class II (Figure 7C), and *Tgfb1* (Figure 7D), with a trend toward reduced expression of cytokines and chemokines, including *Tnfa*, *Il6*, *Ccl2*, and *Cxcl10* (Figure 7E), while the expression of *H2-Kb*, an MHC class I molecule, remained unchanged (Figure S8A). To further depict the signaling pathways underlying the protective effect of overexpressed MANF, we induced β cell dysfunction and death in primary mouse β cells using STZ *in vitro*. Islets isolated from INS-MANF DOX+ mice were cultured in the presence of DOX *in vitro*, whereas INS-MANF DOX− islets were cultured in normal medium and treated with or without STZ. In accordance with our RNA-seq data, overexpression of MANF in islets reduced the expression of UPR pro-apoptotic marker *Ddit3*, along with a trend toward decreased expression of *Atf4* and *Atf5* (Figure 7F). Additionally, MANF overexpression reduced the STZ-induced upregulation of *Txnip in vitro* (Figure 7F). This aligns with our *in vivo* observations, showing increased TXNIP staining in MLDS-injected control, compared with MANF-overexpressing β cells (Figure 4D). To further investigate the relationship between MANF and TXNIP, isolated mouse islets from INS-MANF mice were treated with STZ in the presence or absence of a TXNIP inhibitor SRI-37330[53] or DOX. Both MANF overexpression and TXNIP inhibition attenuated STZ-induced *Ddit3* and *Txnip* expression, with no additional reduction upon combined treatment compared with either treatment alone (Figure 7G, S8B, and S8C). Notably, chronic UPR activation in MANF-deficient islets,[18,26] was associated with increased *Txnip* and *Nrf2* expression (Figures 7H, 7I, S8D, and S8E). Furthermore, the TXNIP protein levels were significantly increased in insulin-positive β cells of 5-week-old $Manf^{-/-}$ mice (Figure 7J). These results suggest that increasing MANF levels in β cells alleviate ER stress and oxidative stress, hindering TXNIP-induced terminal UPR activation, thus promoting β cell survival.

DOX− mice ($|Log_2(FC)| > 0.5$, FDR < 0.05), plotted against islet mRNA from STZ-injected INS-MANF DOX+ and INS-MANF DOX− mice (STZ DOX+ vs. STZ DOX−, $|Log_2(FC)| > 0.25$, $p < 0.05$). Red dots mark the 29 genes downregulated by STZ alone but upregulated by MANF in the STZ DOX+ vs. STZ DOX− group. Blue dots mark the 21 genes that were upregulated by STZ alone but downregulated by MANF in the same groups. (E–G) mRNA expression levels of representative genes from RNA-seq data involved in immune cell activation and recruitment (E), oxidative stress (F), and p53 transcriptional targets (G) among the 21 genes that were upregulated by STZ alone and downregulated by MANF overexpression. (H–M) The relative mRNA expression levels of marker genes for the UPR (H–J), senescence genes *Cdkn1a* (p21) (K) and *Trp53* (p53) (L), and *Trib3* (M) measured by RT-qPCR in isolated WT mouse islets treated with ER stressor thapsigargin (TG) or TG together with rhMANF protein for 16 h. Data are expressed as mean ± SEM, and scatterplots show all individual data points. Statistical analyses were performed using one-way ANOVA with Tukey's post hoc test for (H–M). *$p < 0.05$, **$p < 0.01$, ***$p < 0.001$, ****$p < 0.0001$.

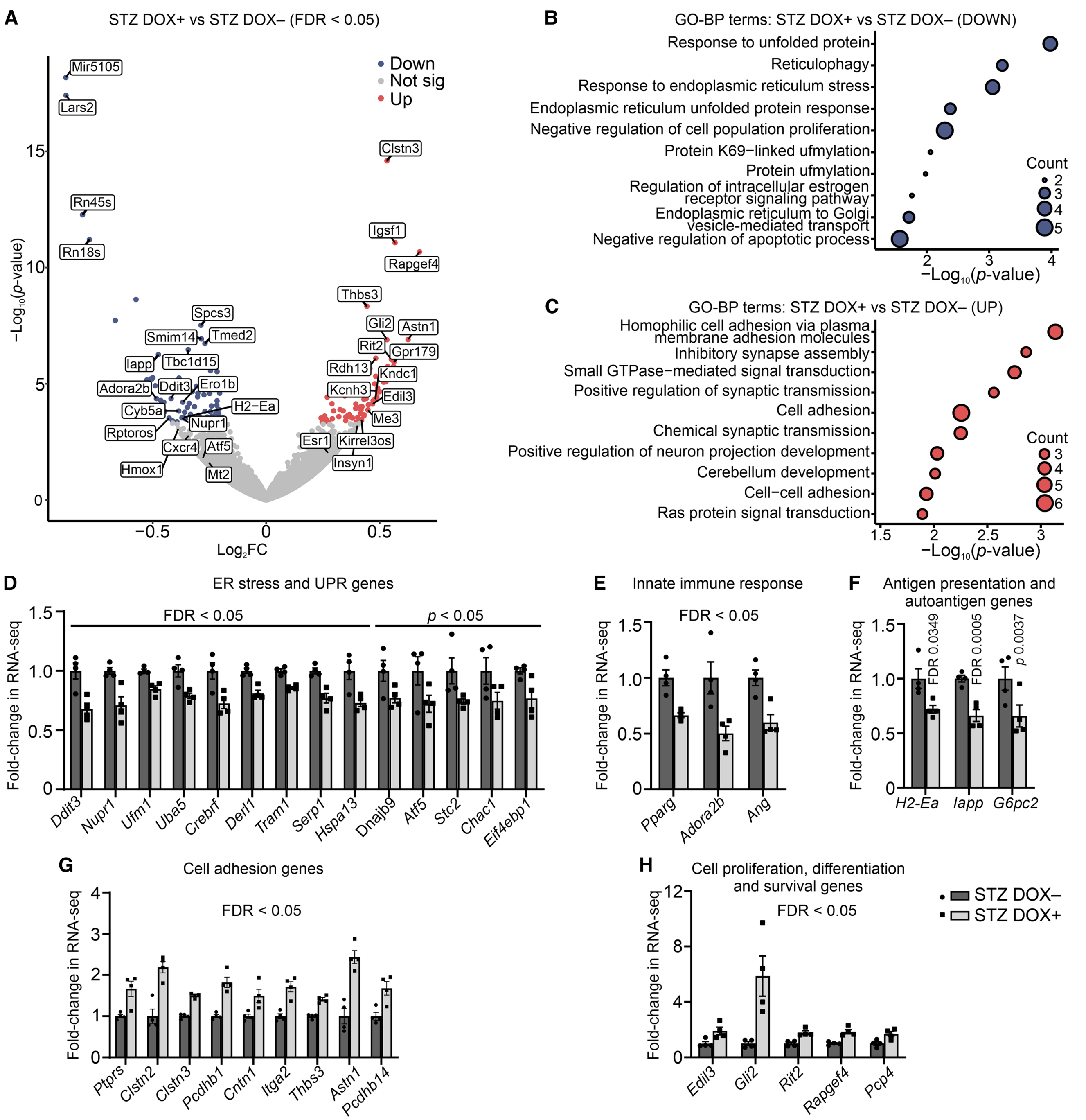

Figure 6. Islets from MANF-overexpressing mice have altered gene expression associated with the UPR, autoantigens, antigen-presenting MHC class II molecule, cell adhesion, and proliferation upon STZ

(A) Volcano plot of the differentially expressed genes in islets analyzed with DESeq2 between STZ (45 mg/kg/day)-treated INS-MANF DOX +/– mice (STZ DOX+ vs. STZ DOX– , $n = 4$ per group). Significantly downregulated DEGs (FDR < 0.05) by MANF overexpression are labeled in blue, while upregulated DEGs are labeled in red. Genes of interest are shown. (B and C) Bubble diagrams of GO enrichment analyses in terms of BP using DAVID tool for the 64 genes downregulated (B), FDR < 0.05, and 75 genes upregulated (C), FDR < 0.05, by MANF overexpression compared with the STZ DOX– group ranked according to p values. (D–H) mRNA expression levels of representative genes from RNA-seq data involved in ER stress and UPR (D), innate immune response (E), antigen presentation and autoantigens (F), cell adhesion (G), and cell proliferation, differentiation, and survival (H). Data are expressed as mean ± SEM, and scatterplots show all individual data points. FDR and p values shown were obtained from differential expression analysis using DESeq2 on RNA-seq data.

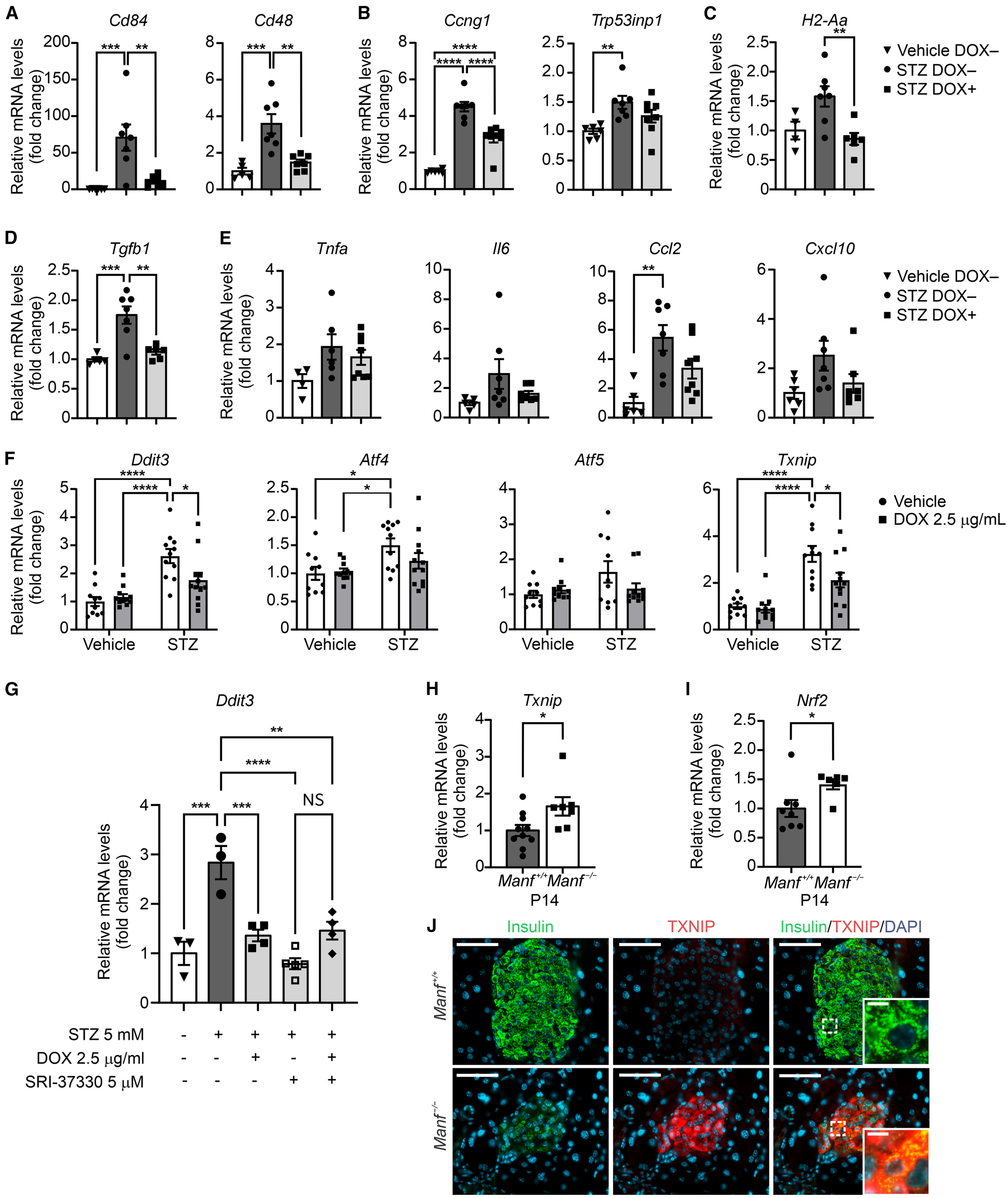

(legend on next page)

Exogenous MANF protein protects mouse β cells against STZ-induced β cell death *in vitro* and *in vivo*

Last, we aimed to study whether exogenously added rhMANF protein, similarly to endogenously overexpressed MANF, confers protection against STZ *in vitro* and *in vivo*. We first evaluated the protective effect of exogenous MANF by treating primary mouse islets isolated from adult wild-type (WT) mice with STZ in the presence of rhMANF protein *in vitro*. Quantification of γH2AX-positive β cells revealed that exogenous MANF efficiently protected against STZ-induced β cell DSBs and death compared with control (Figures 8A and 8B). Similarly, exogenous MANF was able to reduce the expression levels of *Ddit3*, and showed a trend to decrease the expression of *Atf4*, *Atf5*, and *Txnip* in islets treated with STZ (Figure 8C).[9,47] MANF was also found to reduce the expression of the chemokine *Ccl2*, known to recruit immune cells into islets (Figure 8C).[54,55]

We next investigated whether systemic administration of MANF could, similarly to β cell-specific MANF overexpression, confer a therapeutic effect in the MLDS mouse model *in vivo*. Daily subcutaneous MANF treatment for 3 weeks after the first MLDS injection transiently reduced blood glucose levels compared with WT mice, whereas Exendin-4 (Ex-4), a glucagon-like peptide-1 receptor agonist,[56] fully protected against diabetes as previously reported (Figures S9A and S9B). No significant changes in body weight loss were found between groups (Figure S9C). Interestingly, the β cell mass in MANF-injected mice was, similarly to the Ex-4-injected mice, significantly higher than in STZ-injected vehicle-treated mice (Figure S9D). In addition, a tendency toward increased β cell proliferation (Figure S9E), along with significantly reduced β cell death (Figure S9F), was found in the MANF-treated MLDS mice.

DISCUSSION

Our previous studies showed that MANF is indispensable for the survival, proliferation, and function of pancreatic β cells.[17,18,26,27] Interestingly, variants of transcription factor *GLIS3*, associated with both T1D and type 2 diabetes, are known to impede MANF upregulation in stressed β cells resulting in more vulnerable β cells.[57,58] These results indicate that appropriate MANF levels may protect stressed β cells from dysfunction and diabetes. Using our inducible, β cell-specific MANF-overexpressing mouse model, we first showed that MANF overexpressed in β cells effectively hindered β cell death and diabetes after STZ-induced injury, both in the harsher SHDS and the MLDS models resembling T1D. In both models, MANF overexpression preserved the β cell mass, and β cell proliferation was increased after injury compared with control mice. Thus, increased MANF possesses a β cell protective, regenerative, and therapeutic capacity after STZ-induced injury *in vivo*.[18] Furthermore, MANF overexpression in β cells effectively also hindered β cell-mediated insulitis development in the MLDS model. Our data are supported by another report demonstrating that β cell-specific MANF overexpression by gene therapy lowered the rate of insulitis and diabetes incidence in the autoimmune NOD mouse model.[59] In addition, immunophenotyping revealed a reduced number of adaptive immune cells, specifically CD4+ T cells, and a trend toward reduced numbers of macrophages in pLNs of MANF-overexpressing mice at day 4 after the first STZ injection. This likely resulted from attenuated β cell stress and local inflammation, supporting a protective effect of MANF in β cells, limiting early immune recruitment. These results suggest that MANF primarily protects against STZ-induced diabetes by preserving β cell mass, promoting β cell regeneration, and mitigating the local immune response.

STZ induces excess accumulation of ROS causing oxidative and ER stress, DNA damage, and apoptosis.[30] MANF is relatively well studied for its protective effects on damaged cells through alleviation of ER stress.[20] GO enrichment analysis from our RNA-seq data confirmed that the top pathways downregulated by MANF overexpression in islets of STZ-injected mice were related to the UPR and ER stress response. MANF overexpression was associated with reduced expression of genes in the PERK pathway, including pro-apoptotic transcription factor *Ddit3* and stress-inducible *Atf5*, known to regulate the transcription of eukaryotic translation factor 4E-binding protein 1 (*Eif4ebp1*) and *Txnip*. *In vitro*, we confirmed that both exogenous MANF protein and endogenous overexpressed MANF alleviated STZ-induced *Ddit3* and *Txnip* expression in islets. Notably, there is a regulatory interplay between the ER and oxidative stress pathways.[60] IRE1α and PERK hyperactivation induce NRF2, a transcription factor and master regulator of antioxidant cell response.[61,62] In addition, our RNA-seq data showed that the expression of NRF2 targets, *Hmox1* and *Steap1*, induced by STZ, was reduced by MANF overexpression, suggesting attenuated oxidative burden and downregulated compensatory antioxidant defenses. In contrast, MANF-deficient islets showed increased expression of *Nrf2* and *Txnip*. TXNIP is upregulated upon oxidative and ER stress, and binds and oxidizes thioredoxin, a part of the thiol-dependent antioxidant system.[63] Importantly, TXNIP is a promising

Figure 7. Elevated β cell-specific MANF attenuates STZ-induced expression of genes associated with immune activation, p53 targets, ER stress, and oxidative stress, whereas loss of MANF results in increased pro-inflammatory TXNIP expression and oxidative stress in islets

(A–E) Validation of the mRNA levels of marker genes for immune cells (A), p53 transcriptional targets (B), MHC class II molecule *H2-Aa* (C), and cytokines and chemokines (D–E) measured by RT-qPCR in islets isolated from vehicle-injected INS-MANF DOX– mice (Vehicle DOX–, $n = 6$), MLDS-injected (45 mg/kg/day) INS-MANF DOX– (STZ DOX–, $n = 7$), and INS-MANF DOX+ (STZ DOX+, $n = 8$) mice on day 4 after the first MLDS injection. (F) Relative mRNA levels of *Ddit3*, *Atf4*, *Atf5*, and *Txnip* analyzed by RT-qPCR from isolated islets of INS-MANF mice fed with DOX+ or normal chow for 3 months. After recovery in medium ± DOX, islets were exposed to 5 mM STZ for 1 h *in vitro* (n = 10–12 per group). (G) Relative mRNA levels of *Ddit3* after STZ, DOX, and TXNIP inhibitor SRI-37330 treatment (n = 3–5 per group). (H and I) RT-qPCR of *Txnip* (H) and oxidative stress marker *Nrf2* (I) expression in P14 $Manf^{+/+}$ (n = 8–10) and $Manf^{-/-}$ ($n = 7$) islets. (J) Representative pictures of TXNIP (red) and insulin (green) antibody-stained pancreas sections from 5-week-old $Manf^{+/+}$ and littermate $Manf^{-/-}$ mice. Scale bars, 50 and 10 μm (insets). Data are expressed as mean ± SEM, and scatterplots show all individual data points. Statistical analyses were performed using one-way ANOVA with Tukey's post hoc test for (A–E) and (G), two-way ANOVA with Tukey's post hoc test for (F) and Student's two-tailed unpaired t tests for (H) and (I). *$p < 0.05$, **$p < 0.01$, ***$p < 0.001$, ****$p < 0.0001$, NS > 0.05.

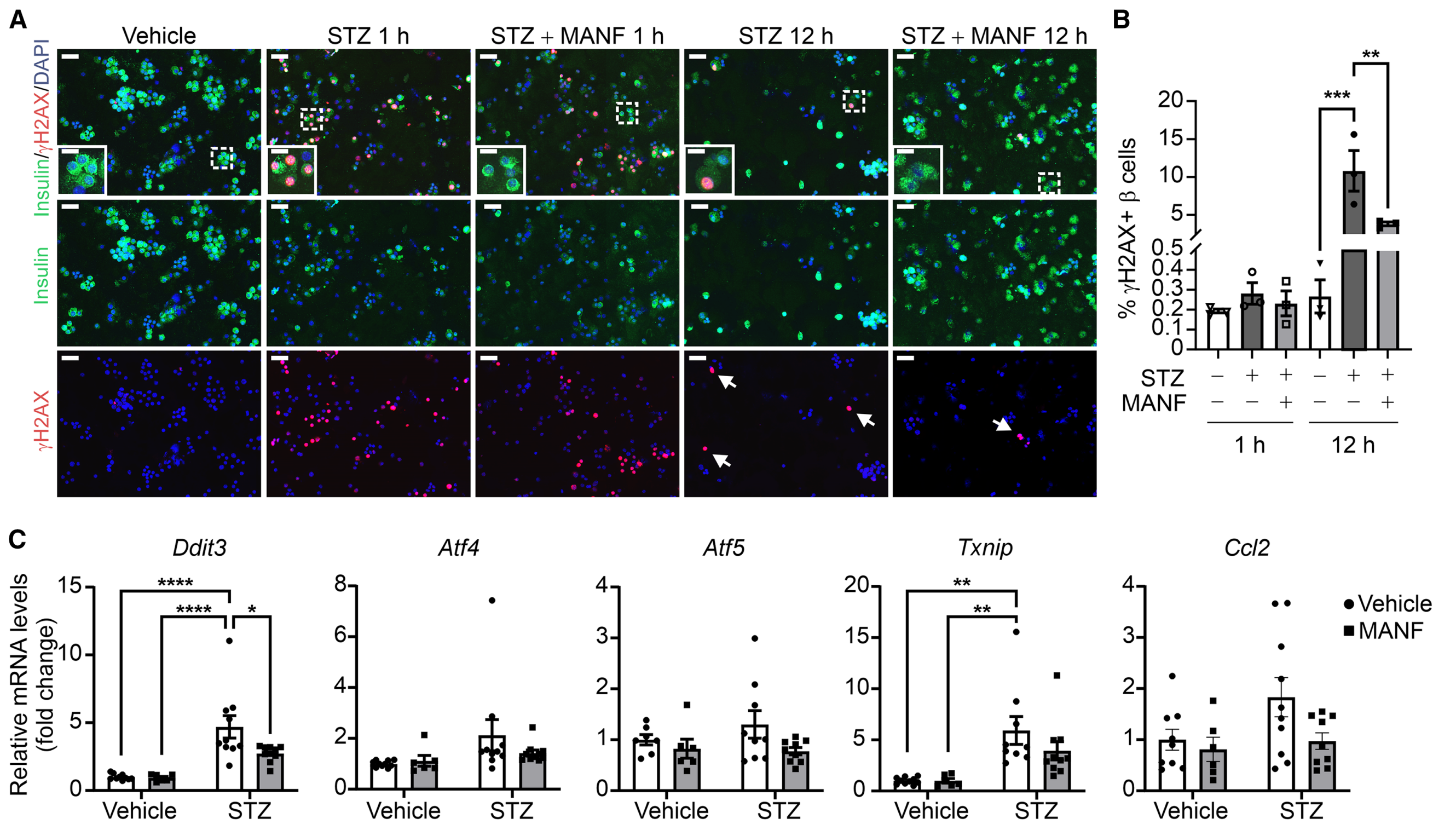

Figure 8. Exogenous MANF protein reduces STZ-induced β cell death, ER stress, and *Txnip* expression *in vitro*

(A) Insulin-stained (green) and γH2AX antibody-stained (red) dissociated islet cells treated with 5 mM STZ in the presence or absence of 200 ng/mL MANF protein for 1 and 12 h *in vitro*. γH2AX-positive cells are marked by white arrows. Scale bars, 50 and 20 μm (insets). (B) Quantification of the ratio of γH2AX-positive β cells 1 and 12 h after STZ treatment (n = 3 per group). (C) Relative mRNA expression levels of *Ddit3*, *Atf4*, *Atf5*, *Txnip*, and *Ccl2* in islets isolated from WT C57BL/6JRccHsd mice stimulated simultaneously with 5 mM STZ with or without 200 ng/mL MANF protein for 1 h *in vitro* (n = 6–10 per group). Data are expressed as mean ± SEM, and scatterplots show all individual data points. Statistical analyses were performed using one-way ANOVA with Tukey's post hoc test for (B) and two-way ANOVA with Tukey's post hoc test for (C). *$p < 0.05$, **$p < 0.01$, ****$p < 0.0001$.

therapeutic target in diabetes. Verapamil, a calcium channel blocker with antidiabetic effect, has been shown to inhibit TXNIP expression.[64] A specific TXNIP inhibitor, TIX100 (SRI-37330), rescues mice from diabetes and is entering phase 1 clinical trials.[53] Using this inhibitor, we observed that MANF overexpression, similarly to TIX100 alone, attenuated STZ-induced *Ddit3* and *Txnip* upregulation, and that combined treatment did not result in further reduction. Our data demonstrate a previously unknown important link between MANF and TXNIP in regulating β cell stress responses. IRE1α and PERK hyperactivation elevates TXNIP expression, leading to a switch from adaptive to terminal UPR and β cell death.[9,47] As MANF binds IRE1α and PERK, and reduces IRE1α oligomerization,[25] our results suggest that appropriate MANF levels in β cells hinder IRE1α and PERK oligomerization, and TXNIP upregulation, thereby preventing terminal UPR, oxidative stress, and β cell death.

STZ directly induces DNA alkylation and the production of free radicals leading to DNA DSBs.[30,65] Phosphorylation of H2AX is one of the first responses to DSBs followed by DDR and phosphorylation of transcription factor p53 to activate p53 responsive genes involved cell-cycle arrest, senescence, and apoptosis in β cells of STZ-injected mice.[66] Importantly, our islet transcriptomics analyses revealed reduced expression of p53-responsive genes in MANF-overexpressing islets, in accordance with the reduced number of γH2AX-positive β cells in pancreas tissue of MLDS-treated MANF-overexpressing mice. In addition, exogenous MANF protein reduced TG-induced *p21* expression in islets *in vitro*. Increased expression of p21 and DDR leads to the accumulation of senescent β cells in T1D.[67] DDR induces an inflammatory response leading to NF-κB activation and the production of pro-inflammatory cytokines and chemokines, contributing to the senescence-associated secretory phenotype (SASP), promoting the innate immune response.[68] Our RNA-seq data show early signs of increased SASP marker expression in islets of MLDS mice (supplemental information). Importantly, transcripts for SASP markers such as *Ccl2*, *Il6* and *Tnfa* were reduced in MANF-overexpressing MLDS islets. In addition, the transcript for TGF-β1, a stress-induced cytokine known to accelerate p53/p21-induced senescence, was reduced.[69] Thus, we have unique evidence that elevated MANF may lead to reduced β cell senescence in T1D. In addition, STZ-induced accumulation of p53 has been shown to inhibit Parkin leading to defect mitophagy, mitochondrial dysfunction, and reduced insulin secretion from β cells.[70] In our

MLDS model, increased MANF in β cells was found to increase the expression of Parkin (*Prkn*) and other autophagy-related genes. Thus, increased MANF may stimulate mitophagy and ER-phagy, thereby relieving cellular stress, in line with our recent study in a tubulointerstitial kidney disease mouse model.[71]

Increased MANF was shown to induce β cell proliferation following STZ-induced injury. Decreasing ER stress and UPR activation by reducing insulin expression in β cells has been shown to promote proliferation by reducing TRIB3 levels, a negative regulator of AKT.[15] Our previous study shows that MANF deficiency in β cells increases ER stress-induced *Trib3*.[26] Therefore, increased MANF may reduce *Trib3* expression and cell-cycle arrest by relieving ER stress, thus inducing β cell proliferation. Additionally, MANF overexpression promoted the expression of components in the RHO/RAS GTPase, RAS/MAPK, and Hedgehog signaling pathways, which are critical for insulin secretion, cell-cycle progression, adhesion, and regeneration.[51,52] Thus, MANF does not only shield β cells from damage but also promotes their functional capacity and proliferation. Clearly, more detailed studies are needed to clarify mitogenic roles for MANF in islets.

STZ triggers a robust autoimmune and inflammatory response, as shown by increased islet expression of immune-related genes in our mRNA-seq data. Several markers for innate and adaptive immune cells and inflammation were upregulated by STZ, in contrast to MANF-overexpressing islets. This is in line with our histological findings of reduced insulitis in MANF-overexpressing islets several weeks after the first STZ injection. Immunophenotyping further revealed a reduced number of activated immune cells in the pLNs. Increased UPR activation in prediabetic T1D human and mouse β cells has been shown to upregulate human leukocyte antigen class I and II expression and increase antigen and neoantigen formation and presentation.[3,14,72,73] Importantly, our data revealed reduced expression of antigen-presenting MHC class II molecules *H2-Ea* and *H2-Aa* in MANF-overexpressing STZ islets. In addition, expression of *G6pc2* and *Iapp*, encoding common autoantigens in T1D,[49,50] was downregulated in islets from STZ-injected MANF-overexpressing mice compared with controls. A trend toward reduced pro-inflammatory cytokine and chemokine expression was detected in MANF-overexpressing islets, consistent with prior findings showing that MANF reduces NF-κB-regulated genes and pathways in human islets *in vitro*.[17] Together, these results confirm that elevated MANF reduces the UPR signaling, autoantigen expression, and antigen presentation, thereby limiting adaptive T cell recruitment, accompanied by the reduced number of CD4+ T cells and macrophages in pLNs of STZ-induced prediabetic mice. Similar data were found in a collaborative study showing the protective effect of the C-terminal fragment of MANF in an *ex vivo* model of multiple sclerosis with transcriptional changes highly similar to our RNA-seq data, confirming the effect of MANF to attenuate stress- and inflammation-related gene programs.[74]

Last, we wanted to explore the protective effect of exogenous MANF compared with endogenously overexpressed MANF in β cells. Consistent with the elevated transgenic MANF, exogenous MANF protected against STZ-induced DSBs, ER stress, and *Txnip* expression in β cells *in vitro*. Despite only a transient and modest reduction in blood glucose levels upon systemic delivery of recombinant MANF *in vivo*, we detected an increase in β cell mass and reduced β cell death, indicating a spatial but insufficient therapeutic benefit in the MLDS model. Nonetheless, we highlight functional similarities between endogenous and exogenous MANF, yet also underscore important limitations related to protein stability and uptake in systemic delivery. Together, these results suggest that sustained, localized expression of MANF may be required for optimal therapeutic efficacy in preserving β cell function and preventing diabetes progression.

However, translation of β cell-specific MANF overexpression to humans is challenged by species-specific immune and metabolic differences, the multifactorial nature of human diabetes, and unresolved issues related to gene delivery, safety, and long-term efficacy. Identifying small-molecule compounds that mimic MANF activity by relieving cellular stress and TXNIP-mediated dysfunction may provide a more readily translatable therapeutic strategy. Furthermore, efforts to restore β cell mass in T1D must combine strategies that stimulate regeneration of residual β cells with approaches that attenuate the β cell-directed autoimmunity. Our data demonstrate that elevated MANF protects β cells by alleviating cellular stress, limiting stress-induced autoimmune responses, and enhancing β cell proliferation. These data position MANF as an attractive candidate for next-generation therapeutic approaches, including engineering stem cell-derived islets with increased stress resilience for replacement therapies or developing gene therapy approaches to selectively increase MANF levels in remaining human β cells.

MATERIALS AND METHODS

Animals and generation of transgenic mice

The cDNA for human MANF (540 bp) was amplified by PCR from a plasmid previously described,[75] using the following primers, F 5′-CCT GCA GCT ACA AAT CGG TCC GTG-3′, R 5′-CGC TGC AGC TAC AAA TCG GTC CGT G-3′, cloned into pCRII (Thermo Fisher Scientific, Waltham, MA) and subcloned into a Tet-op-mp1 pBS-SK vector (a gift from Prof. Katerina Politi, New Haven, CT, and Prof. Harold E. Varmus, New York, NY).[76,77] Functionality of the TET-On system was tested in CHO cells co-transfected with TET-MANF and PB-CA-rtTA-On vectors (Addgene, Cambridge, MA) in the presence of 2.5 μg/mL of DOX (D9891, Sigma-Aldrich, St. Louis, MO) in 70% ethanol, followed by western blotting (Figures S1C and S1D). A transgene fragment of 1,600 bp was purified and injected into fertilized oocytes from FVB mice. TET-MANF transgenic founders were identified by Southern blotting and PCR genotyping, and copy number was estimated by Southern blotting and RT-qPCR using primer sequences for human MANF F 5′-ATG ATG CAG CCA AAA TC-3′, R 5′-CAG ATC TTC TCC ACA GGG ATG-3′ (amplicon 73 nt), and actin F 5′-CAG CCA ACT TTA CGC CTA GC-3′, R 5′-GGG CCC ACG AGT GTC TAC-3′. To generate bi-transgenic INS-MANF mice, hemizygous TET-MANF mice were crossbred with hemi- or homozygous INS-rtTA mice in an FVB background (gift

from Timo Otonkoski, Biomedicum Stem Cell Center, University of Helsinki, Helsinki, Finland).[78,79] MANF overexpression was induced in β cells by a DOX diet (Envigo TD.01306 irradiated Teklad rodent diet 2018 with 625 mg/kg DOX). One line with 4–6 MANF gene copies showing the highest expression was selected.

The WT C57BL/6JRccHsd mice were purchased from Envigo, Indiana. Global *Manf*$^{-/-}$ mice and pancreas-specific MANF KO (*Pdx-1Cre*$^{+/-}$*::Manf*$^{fl/fl}$) were generated as previously described.[18,26] Since we used STZ to induce diabetes and male mice are more sensitive to STZ than female mice, likely due to hormonal differences,[30] we focused on characterizing bi-transgenic male mice with higher β cell-specific MANF overexpression (Figure 1). MANF expression levels were about three times higher in islets isolated from male mice compared with female mice (Figures S1A–S1F).

All experimental procedures involving mice were conducted in accordance with the ARRIVE 2.0 guidelines and Declaration of Helsinki and approved by the Regional State Administrative Agency for Southern Finland, protocols ESAVI/11997/2023, ESAVI/9523/2020, ESAVI/3117/04.10.07/2017, and ESAVI/2629/04.10.07/2017.

Genotyping

Genomic DNA was isolated from earmarks using an Extracta DNA Prep for PCR kit (95091, Quantabio, Beverly, MA) and genotyping was carried out by PCR using the following primers, TET-MANF mice: F 5′-ATC CAC GCT GTT TTG ACC TC-3′, R 5′-ACG CAG GAG TTT TGA TGG AC-3′ generating a 991-bp transgene band, INS-rtTA mice: F 5′-TAG ATG TGC TTT ACT AAG TCA TCG CG-3′, R 5′-GAG ATC GAG CAG GCC CTC GAT GGT AG-3′ generating a 400-bp transgene band. *Pdx-1Cre*$^{+/-}$*::Manf*$^{fl/fl}$, *Manf*$^{fl/fl}$, and *Manf*$^{-/-}$ mice were genotyped as previously described.[18]

Blood parameters and ELISAs

Blood was collected from the tail tip for immediate glucose measurement using a clinical glucometer (Accucheck Aviva Glucometer, Roche Diagnostics, Basel, Switzerland). Fasting glucose levels were measured after 4 h fasting. At endpoint, terminal blood was collected via cardiac puncture for insulin measurement (ultrasensitive mouse insulin ELISA, 90080, Crystal Chem, Elk Grove Village, IL), and human MANF quantification in islets using an in-house sandwich ELISA specific for human MANF as previously described.[80]

Intraperitoneal glucose tolerance tests

Intraperitoneal glucose tolerance tests were performed as previously described.[18] Mice fasted for 6–12 h before intraperitoneal injection of glucose (2 mg/g body weight, 20%, w/v) and tail vein glucose levels were monitored at 0, 30, 60, 90, and 120 min using the Accucheck Aviva Glucometer.

In vivo STZ injections and systemic MANF administration in mice

INS-MANF and sTG mice were fed with DOX or normal chow for 3 months before STZ injections. STZ (S0130, Sigma-Aldrich) was freshly diluted in a 25 mmol/L sodium citrate buffer (pH 4.5) and injected intraperitoneally at 180 mg/kg body weight as a single dose, with buffer as control. In the MLDS model, STZ was administered at 45–50 mg/kg body weight per day for 5 consecutive days. For exogenous MANF therapy, daily subcutaneous injections of MANF (P-101-100, Icosagen, Tartu, Estonia, 0.7 mg/kg/day in PBS) were started 5 days before MLDS injections and continued for 21 days in 12-week-old male C57BL/6JRcc mice. Ex-4 (E7144, Sigma-Aldrich, 0.1 mg/kg/day) was used as a positive control. Mice were maintained for an additional week without MANF or Ex-4 and sacrificed 23 days after the first STZ injection. To prevent hypoglycemia, 10% (w/v) sucrose (S0389, Sigma-Aldrich) water was provided for 1–2 days post-injection. Blood glucose levels and body weight were measured before the first STZ injection and two to three times weekly until sacrifice.

Immunohistochemistry, immunofluorescence, and analyses of α or β cell mass

Pancreas and brain tissues were post-fixed in 4% paraformaldehyde (PFA) for 2–4 days, paraffin-embedded, and sectioned at 5 μm. Sections were subjected to antigen retrieval in 10 mM sodium citrate buffer (pH 6.0) followed by staining with primary antibodies (Table S3). Biotinylated or Alexa Fluor 488/568-conjugated secondary antibodies were used for immunohistochemistry or immunofluorescence, respectively. α and β cell mass was analyzed from four serial pancreatic sections (one per 200 μm) stained with anti-glucagon or anti-insulin antibodies, as described (Table S3), and analyzed using the Image-Pro Plus program. β Cell proliferation and STZ-induced active DDR ultimately leading to apoptosis were assessed via Ki67- or γH2AX co-staining with insulin (Table S3). Images were captured with a 3DHISTECH Panoramic 250 FLASH II (Budapest, Hungary) or a Zeiss AxioImager M2 microscope (Zeiss, Oberkochen, Germany) and analyzed with CaseViewer or ZEN 3.1 software (Zeiss).

Insulitis scoring

Pancreases were dissected, fixed in 4% PFA for 2–4 days, paraffin-embedded, and sectioned in 5 μm sections. Four sections per pancreas, 200 μm apart, were used for hematoxylin and eosin staining to score islet insulitis. From each pancreas, 16–32 islets (4–8 randomly selected per section) were scored blindly by two independent observers. Islets (>20 cells) were classified as: (1) normal (<1% infiltration), (2) minor infiltration (1%–10%), (3) minor peri-insulitis (10%–25%), (4) peri-insulitis (25%–50%), or (5) insulitis (50%–100%). The percentage of islets in each score was calculated relative to the total evaluated.

Islets isolation and *in vitro* treatment

Pancreases were digested with 1 mg/mL collagenase P (11213857001, Roche Diagnostics) and 0.25 mg/mL DNase I (11284932001, Roche Diagnostics), and islets were handpicked as previously described.[18] Islets from 10- to 12-week-old C57BL/6JRcc male mice were recovered overnight in RPMI 1640 with 10% FBS and 1% PenStrep (100 U/mL penicillin and 100 μg/mL streptomycin), then starved in 0.5% BSA for 3 h. They were stimulated with or without 5 mM STZ (in 10 mM citrate buffer [pH 4.5]) with or without 200 ng/mL

MANF for 1 or 12 h. After 1 h, islets were collected for RNA isolation and RT-qPCR; after 12 h, cells were dispersed, cytospun, fixed,[18] and stained for insulin and γH2AX (Table S3) to quantify DNA damage in β cells. Islets from INS-MANF mice fed with DOX or normal chow were treated similarly for STZ experiments, followed by RNA isolation and RT-qPCR. For TXNIP inhibitor treatment, islets isolated from INS-MANF or control mice were treated with 5 mM STZ and 5 μM SRI-37330 (HY-142114, MedChemExpress, Monmouth Junction, NJ) with or without 2.5 μg/mL DOX for 1 h. For TG treatment, islets were treated with 2 μM TG (T7459, Invitrogen, Thermo Fisher Scientific) with or without MANF protein for 16 h.

RT-qPCR

Hypothalamic tissue was collected via cryostat-assisted punches guided by Allen Mouse Brain Atlas (2008) for precise localization. RNA from islets or hypothalamic tissue was isolated by TRIzol Reagent (15596026, Invitrogen, Thermo Fisher Scientific) and quantitated by NanoDrop (NanoDrop Technologies, Wilmington, DE). Reverse transcription was performed with RevertAid Premium Reverse Transcriptase (EP0753, Thermo Fisher Scientific) and oligo(dT)18 Primer (500 μg/mL) at 55°C for 40 min. Quantitative RT-PCR was performed using LightCycler 480 SYBR Green I Master mix (04887352001, Roche Diagnostics) on a LightCycler 480 Real-Time PCR system (Roche Diagnostics, Rotkreuz, Switzerland), with expression levels normalized to β-actin. Primer sequences are listed in Table S4.

Bulk RNA-seq

INS-MANF and sTG mice were fed with DOX or normal chow for 12 weeks, followed by MLDS (45 mg/kg body weight/day) or vehicle for 3 days. Islets were isolated on day 4, and total RNA was extracted using the QIAGEN RNeasy Micro kit (74004, QIAGEN, Hilden, Germany) according to the manufacturer's instructions. Poly(A)-selected mRNA-seq was performed by Azenta Life Sciences (Leipzig, Germany). Data were processed on the Galaxy Community platform[81]; fastq files were quality checked with fastp v.0.23.2, aligned to the built-in mouse mm10 reference genome using HISAT2 v.2.2.1, and transcripts counted with FeatureCounts v.1.6.4. Differential gene expression analyses were performed using DESeq2 v.22.11.40.6 and gene IDs converted via SynGO portal.[82] GO enrichment analysis was performed using DAVID,[83,84] and graphs were created using R package ggplot2. Heatmaps in Figure 5C show relative average expression of selected genes, calculated from the 643 DEGs between STZ DOX– and vehicle DOX– mice (Table S1).

Immunophenotyping by flow cytometry

INS-MANF and sTG mice were fed with DOX or normal chow for 12 weeks, followed by MLDS (45 mg/kg body weight/day) or vehicle injections for 3 or 5 consecutive days. pLNs and spleens were collected on days 4 or 10, and single-cell suspensions were prepared by physical dissociation through 100-μm cell strainers (Corning, New York), followed by red blood cell lysis using RBC Lysis Buffer (00-4300-54, eBioscience, Thermo Fisher Scientific) lysis. Cells (1×10^6/sample) were Fc blocked using Mouse BD Fc Block (BD Biosciences, San Jose, CA) and stained with antibodies (Table S5). Data were acquired on an LSRFortessa Cell Analyzer (BD Biosciences) and analyzed with BD FACSDiva (BD Biosciences) and FlowJo software (Tree Star) using appropriate controls for gating (Figure S5).

Statistical analysis

Data are presented as mean ± SEM. Sample size and statistics for each experiment are included in the figure legends. Statistical comparisons between two groups were performed with Student's unpaired *t* test. One-way analysis of variance (ANOVA) followed by Tukey's post hoc test was used for three or more groups. We conducted two-way ANOVA to evaluate the effects of two independent factors, treatment and time, followed by Bonferroni's post hoc test for comparisons involving two groups, Tukey's post hoc test or Holm-Šidák's multiple comparison test when more than two groups were involved. The p values < 0.05 were considered significant. Statistical analyses were performed with GraphPad Prism 10.

DATA AND CODE AVAILABILITY

- Data are available within the published article and supplemental information. RNA-seq data are available in Tables S1 and S2.
- Additional data are available from the corresponding author upon reasonable request.

ACKNOWLEDGMENTS

We thank Sari Tynkkynen for valuable technical assistance; NIH KOMP for the MANF-targeted embryonic stem cell clone used to develop the MANF knockout mice; T.O. for INSrtTA mice; Diego Balboa (University of Helsinki, Finland) for the PB-CA-rtTA-On vector; Katerina Politi (New Haven, CT) and Harold E. Varmus (New York, NY) for the Tet-op-mp1 pBS-SK vector; Mart Saarma (University of Helsinki, Finland) for valuable support and sharing reagents and equipment; Päivi Lindholm-Pulkkila (University of Helsinki, Finland) for providing in-house sandwich human MANF ELISA and critical comments; Ulla Pirvola (University of Helsinki, Finland) for use of the Zeiss AxioImager M2 epifluorescence microscope. This work was carried out with the support of HiLIFE Laboratory Animal Center Core Facility and HiLIFE Viikki Flow Cytometry unit, both at the University of Helsinki, Finland. This work was supported by grants from the Research Council of Finland (Academy of Finland 333974), the Finnish Diabetes Research Foundation, and Breakthrough T1D (formerly the Juvenile Diabetes Research Foundation) (17-2013-410, 2-SRA-2018-496-A-B, 2-SRA-2022-1202-S-B). The graphical abstract was created in BioRender (https://BioRender.com/5kus9hz).

AUTHOR CONTRIBUTIONS

H.L., T.D., E. Pakarinen, J.K., and M.L. performed *in vivo* experiments and *in vitro* islet experiments, RT-qPCR, and immunostaining. E. Palm generated the TET-MANF transgenic plasmid and tested genotyping primers. E. Pakarinen performed *in vitro* experiments, mouse brain dissections, and antibody staining of brain sections. J.K., E. Pakarinen, and M.L. isolated primary mouse islets for RNA-seq. I.T. provided expertise and practical information on RNA-seq. H.L. and T. Org analyzed and visualized RNA-seq data. T.K.K. and M.H.V. provided expertise in immunophenotyping. H.L. and J.K. performed the immunophenotyping experiment and T.K.K. analyzed the results. E.H. contributed with information for INS-rtTA mice and insulitis-scoring methodologies. T. Otonkoski provided INS-rtTA mice and the initial idea for creating MANF transgenic mice. H.L. performed image processing, statistical analysis of data and interpretation, and figure preparation. M.L. supervised the project, designed experiments, TET-MANF transgenic plasmid construct, animal breeding, performed experiments, and interpreted data. H.L. and M.L. drafted the manuscript. All authors reviewed and edited the manuscript.

DECLARATION OF INTERESTS

The authors declare no competing interests.

SUPPLEMENTAL INFORMATION

Supplemental information can be found online at https://doi.org/10.1016/j.ymthe.2026.02.043.

REFERENCES

1. Piganelli, J.D., Mamula, M.J., and James, E.A. (2020). The Role of beta Cell Stress and Neo-Epitopes in the Immunopathology of Type 1 Diabetes. Front. Endocrinol. *11*, 624590. https://doi.org/10.3389/fendo.2020.624590.
2. Eizirik, D.L., Pasquali, L., and Cnop, M. (2020). Pancreatic beta-cells in type 1 and type 2 diabetes mellitus: different pathways to failure. Nat. Rev. Endocrinol. *16*, 349–362. https://doi.org/10.1038/s41574-020-0355-7.
3. Eizirik, D.L., Szymczak, F., Alvelos, M.I., and Martin, F. (2021). From Pancreatic beta-Cell Gene Networks to Novel Therapies for Type 1 Diabetes. Diabetes *70*, 1915–1925. https://doi.org/10.2337/dbi20-0046.
4. Roep, B.O., Thomaidou, S., van Tienhoven, R., and Zaldumbide, A. (2021). Type 1 diabetes mellitus as a disease of the beta-cell (do not blame the immune system?). Nat. Rev. Endocrinol. *17*, 150–161. https://doi.org/10.1038/s41574-020-00443-4.
5. Wang, M., and Kaufman, R.J. (2016). Protein misfolding in the endoplasmic reticulum as a conduit to human disease. Nature *529*, 326–335. https://doi.org/10.1038/nature17041.
6. Merksamer, P.I., Trusina, A., and Papa, F.R. (2008). Real-time redox measurements during endoplasmic reticulum stress reveal interlinked protein folding functions. Cell *135*, 933–947. https://doi.org/10.1016/j.cell.2008.10.011.
7. Papa, F.R. (2012). Endoplasmic reticulum stress, pancreatic beta-cell degeneration, and diabetes. Cold Spring Harb. Perspect. Med. *2*, a007666. https://doi.org/10.1101/cshperspect.a007666.
8. Urano, F., Wang, X., Bertolotti, A., Zhang, Y., Chung, P., Harding, H.P., and Ron, D. (2000). Coupling of stress in the ER to activation of JNK protein kinases by transmembrane protein kinase IRE1. Science *287*, 664–666. https://doi.org/10.1126/science.287.5453.664.
9. Lerner, A.G., Upton, J.P., Praveen, P.V.K., Ghosh, R., Nakagawa, Y., Igbaria, A., Shen, S., Nguyen, V., Backes, B.J., Heiman, M., et al. (2012). IRE1alpha induces thioredoxin-interacting protein to activate the NLRP3 inflammasome and promote programmed cell death under irremediable ER stress. Cell Metab. *16*, 250–264. https://doi.org/10.1016/j.cmet.2012.07.007.
10. Deng, J., Lu, P.D., Zhang, Y., Scheuner, D., Kaufman, R.J., Sonenberg, N., Harding, H.P., and Ron, D. (2004). Translational repression mediates activation of nuclear factor kappa B by phosphorylated translation initiation factor 2. Mol. Cell. Biol. *24*, 10161–10168. https://doi.org/10.1128/MCB.24.23.10161-10168.2004.
11. Lee, H., Lee, Y.S., Harenda, Q., Pietrzak, S., Oktay, H.Z., Schreiber, S., Liao, Y., Sonthalia, S., Ciecko, A.E., Chen, Y.G., et al. (2020). Beta Cell Dedifferentiation Induced by IRE1alpha Deletion Prevents Type 1 Diabetes. Cell Metab *31*, 822–836.e825. https://doi.org/10.1016/j.cmet.2020.03.002.
12. Thomaidou, S., Kracht, M.J.L., van der Slik, A., Laban, S., de Koning, E.J., Carlotti, F., Hoeben, R.C., Roep, B.O., and Zaldumbide, A. (2020). beta-Cell Stress Shapes CTL Immune Recognition of Preproinsulin Signal Peptide by Posttranscriptional Regulation of Endoplasmic Reticulum Aminopeptidase 1. Diabetes *69*, 670–680. https://doi.org/10.2337/db19-0984.
13. Li, Y., Sun, F., Yue, T.T., Wang, F.X., Yang, C.L., Luo, J.H., Rong, S.J., Xiong, F., Zhang, S., and Wang, C.Y. (2021). Revisiting the Antigen-Presenting Function of beta Cells in T1D Pathogenesis. Front. Immunol. *12*, 690783. https://doi.org/10.3389/fimmu.2021.690783.
14. Thompson, P.J., Pipella, J., Rutter, G.A., Gaisano, H.Y., and Santamaria, P. (2023). Islet autoimmunity in human type 1 diabetes: initiation and progression from the perspective of the beta cell. Diabetologia *66*, 1971–1982. https://doi.org/10.1007/s00125-023-05970-z.
15. Szabat, M., Page, M.M., Panzhinskiy, E., Skovsø, S., Mojibian, M., Fernandez-Tajes, J., Bruin, J.E., Bround, M.J., Lee, J.T.C., Xu, E.E., et al. (2016). Reduced Insulin Production Relieves Endoplasmic Reticulum Stress and Induces beta Cell Proliferation. Cell Metab. *23*, 179–193. https://doi.org/10.1016/j.cmet.2015.10.016.
16. Danilova, T., Galli, E., Pakarinen, E., Palm, E., Lindholm, P., Saarma, M., and Lindahl, M. (2019). Mesencephalic Astrocyte-Derived Neurotrophic Factor (MANF) Is Highly Expressed in Mouse Tissues With Metabolic Function. Front. Endocrinol. *10*, 765. https://doi.org/10.3389/fendo.2019.00765.
17. Hakonen, E., Chandra, V., Fogarty, C.L., Yu, N.Y.L., Ustinov, J., Katayama, S., Galli, E., Danilova, T., Lindholm, P., Vartiainen, A., et al. (2018). MANF protects human pancreatic beta cells against stress-induced cell death. Diabetologia *61*, 2202–2214. https://doi.org/10.1007/s00125-018-4687-y.
18. Lindahl, M., Danilova, T., Palm, E., Lindholm, P., Võikar, V., Hakonen, E., Ustinov, J., Andressoo, J.O., Harvey, B.K., Otonkoski, T., et al. (2014). MANF is indispensable for the proliferation and survival of pancreatic beta cells. Cell Rep. *7*, 366–375. https://doi.org/10.1016/j.celrep.2014.03.023.
19. Jäntti, M., and Harvey, B.K. (2020). Trophic activities of endoplasmic reticulum proteins CDNF and MANF. Cell Tissue Res. *382*, 83–100. https://doi.org/10.1007/s00441-020-03263-0.
20. Danilova, T., and Lindahl, M. (2018). Emerging Roles for Mesencephalic Astrocyte-Derived Neurotrophic Factor (MANF) in Pancreatic Beta Cells and Diabetes. Front. Physiol. *9*, 1457. https://doi.org/10.3389/fphys.2018.01457.
21. Bai, M., Vozdek, R., Hnízda, A., Jiang, C., Wang, B., Kuchar, L., Li, T., Zhang, Y., Wood, C., Feng, L., et al. (2018). Conserved roles of C. elegans and human MANFs in sulfatide binding and cytoprotection. Nat. Commun. *9*, 897. https://doi.org/10.1038/s41467-018-03355-0.
22. Kitamura, R.A., Hummel, D., Ustione, A., Piston, D.W., and Urano, F. (2024). Dual role of neuroplastin in pancreatic beta cells: Regulating insulin secretion and promoting islet inflammation. Proc. Natl. Acad. Sci. USA *121*, e2411234121. https://doi.org/10.1073/pnas.2411234121.
23. Glembotski, C.C., Thuerauf, D.J., Huang, C., Vekich, J.A., Gottlieb, R.A., and Doroudgar, S. (2012). Mesencephalic astrocyte-derived neurotrophic factor protects the heart from ischemic damage and is selectively secreted upon sarco/endoplasmic reticulum calcium depletion. J. Biol. Chem. *287*, 25893–25904. https://doi.org/10.1074/jbc.M112.356345.
24. Eesmaa, A., Yu, L.Y., Göös, H., Nõges, K., Kovaleva, V., Hellman, M., Zimmermann, R., Jung, M., Permi, P., Varjosalo, M., et al. (2021). The cytoprotective protein MANF promotes neuronal survival independently from its role as a GRP78 cofactor. J. Biol. Chem. *296*, 100295. https://doi.org/10.1016/j.jbc.2021.100295.
25. Kovaleva, V., Yu, L.Y., Ivanova, L., Shpironok, O., Nam, J., Eesmaa, A., Kumpula, E.P., Sakson, S., Toots, U., Ustav, M., et al. (2023). MANF regulates neuronal survival and UPR through its ER-located receptor IRE1alpha. Cell Rep. *42*, 112066. https://doi.org/10.1016/j.celrep.2023.112066.
26. Danilova, T., Belevich, I., Li, H., Palm, E., Jokitalo, E., Otonkoski, T., and Lindahl, M. (2019). MANF Is Required for the Postnatal Expansion and Maintenance of Pancreatic beta-Cell Mass in Mice. Diabetes *68*, 66–80. https://doi.org/10.2337/db17-1149.
27. Montaser, H., Patel, K.A., Balboa, D., Ibrahim, H., Lithovius, V., Näätänen, A., Chandra, V., Demir, K., Acar, S., Ben-Omran, T., et al. (2021). Loss of MANF Causes Childhood-Onset Syndromic Diabetes Due to Increased Endoplasmic Reticulum Stress. Diabetes *70*, 1006–1018. https://doi.org/10.2337/db20-1174.
28. Cunha, D.A., Cito, M., Grieco, F.A., Cosentino, C., Danilova, T., Ladrière, L., Lindahl, M., Domanskyi, A., Bugliani, M., Marchetti, P., et al. (2017). Pancreatic beta-cell protection from inflammatory stress by the endoplasmic reticulum proteins thrombospondin 1 and mesencephalic astrocyte-derived neutrotrophic factor (MANF). J. Biol. Chem. *292*, 14977–14988. https://doi.org/10.1074/jbc.M116.769877.
29. Ahn, C., An, B.S., and Jeung, E.B. (2015). Streptozotocin induces endoplasmic reticulum stress and apoptosis via disruption of calcium homeostasis in mouse pancreas. Mol. Cell. Endocrinol. *412*, 302–308. https://doi.org/10.1016/j.mce.2015.05.017.
30. Goyal, S.N., Reddy, N.M., Patil, K.R., Nakhate, K.T., Ojha, S., Patil, C.R., and Agrawal, Y.O. (2016). Challenges and issues with streptozotocin-induced diabetes - A clinically relevant animal model to understand the diabetes pathogenesis and evaluate therapeutics. Chem. Biol. Interact. *244*, 49–63. https://doi.org/10.1016/j.cbi.2015.11.032.
31. Yamamoto, H., Uchigata, Y., and Okamoto, H. (1981). Streptozotocin and alloxan induce DNA strand breaks and poly(ADP-ribose) synthetase in pancreatic islets. Nature *294*, 284–286. https://doi.org/10.1038/294284a0.
32. Kantwerk-Funke, G., Burkart, V., and Kolb, H. (1991). Low dose streptozotocin causes stimulation of the immune system and of anti-islet cytotoxicity in mice. Clin. Exp. Immunol. *86*, 266–270. https://doi.org/10.1111/j.1365-2249.1991.tb05808.x.

33. Sun, Y., Chen, X., and Xiao, D. (2007). Tetracycline-inducible expression systems: new strategies and practices in the transgenic mouse modeling. Acta Biochim. Biophys. Sin. *39*, 235–246. https://doi.org/10.1111/j.1745-7270.2007.00258.x.

34. Wicksteed, B., Brissova, M., Yan, W., Opland, D.M., Plank, J.L., Reinert, R.B., Dickson, L.M., Tamarina, N.A., Philipson, L.H., Shostak, A., et al. (2010). Conditional gene targeting in mouse pancreatic ss-Cells: analysis of ectopic Cre transgene expression in the brain. Diabetes *59*, 3090–3098. https://doi.org/10.2337/db10-0624.

35. Horwitz, E., Krogvold, L., Zhitomirsky, S., Swisa, A., Fischman, M., Lax, T., Dahan, T., Hurvitz, N., Weinberg-Corem, N., Klochendler, A., et al. (2018). beta-Cell DNA Damage Response Promotes Islet Inflammation in Type 1 Diabetes. Diabetes *67*, 2305–2318. https://doi.org/10.2337/db17-1006.

36. Rahmanian, N., Shokrzadeh, M., and Eskandani, M. (2021). Recent advances in gammaH2AX biomarker-based genotoxicity assays: A marker of DNA damage and repair. DNA Repair (Amst) *108*, 103243. https://doi.org/10.1016/j.dnarep.2021.103243.

37. Anthony, T.G., and Wek, R.C. (2012). TXNIP switches tracks toward a terminal UPR. Cell Metab. *16*, 135–137. https://doi.org/10.1016/j.cmet.2012.07.012.

38. Choi, E.H., and Park, S.J. (2023). TXNIP: A key protein in the cellular stress response pathway and a potential therapeutic target. Exp. Mol. Med. *55*, 1348–1356. https://doi.org/10.1038/s12276-023-01019-8.

39. Masson, E., Koren, S., Razik, F., Goldberg, H., Kwan, E.P., Sheu, L., Gaisano, H.Y., and Fantus, I.G. (2009). High beta-cell mass prevents streptozotocin-induced diabetes in thioredoxin-interacting protein-deficient mice. Am. J. Physiol. Endocrinol. Metab. *296*, E1251–E1261. https://doi.org/10.1152/ajpendo.90619.2008.

40. Luo, Z., Soläng, C., Mejia-Cordova, M., Thorvaldson, L., Blixt, M., Sandler, S., and Singh, K. (2019). Kinetics of immune cell responses in the multiple low-dose streptozotocin mouse model of type 1 diabetes. FASEB Bioadv. *1*, 538–549. https://doi.org/10.1096/fba.2019-00031.

41. Wållberg, M., and Cooke, A. (2013). Immune mechanisms in type 1 diabetes. Trends Immunol. *34*, 583–591. https://doi.org/10.1016/j.it.2013.08.005.

42. Gagnerault, M.C., Luan, J.J., Lotton, C., and Lepault, F. (2002). Pancreatic lymph nodes are required for priming of beta cell reactive T cells in NOD mice. J. Exp. Med. *196*, 369–377. https://doi.org/10.1084/jem.20011353.

43. Kim, Y.T., and Steinberg, C. (1984). Immunologic studies on the induction of diabetes in experimental animals. Cellular basis for the induction of diabetes by streptozotocin. Diabetes *33*, 771–777. https://doi.org/10.2337/diab.33.8.771.

44. Lukić, M.L., Stosić-Grujicić, S., and Shahin, A. (1998). Effector mechanisms in low-dose streptozotocin-induced diabetes. Dev. Immunol. *6*, 119–128. https://doi.org/10.1155/1998/92198.

45. Suckow, A.T., Comoletti, D., Waldrop, M.A., Mosedale, M., Egodage, S., Taylor, P., and Chessler, S.D. (2008). Expression of neurexin, neuroligin, and their cytoplasmic binding partners in the pancreatic beta-cells and the involvement of neuroligin in insulin secretion. Endocrinology *149*, 6006–6017. https://doi.org/10.1210/en.2008-0274.

46. Pingitore, A., Caroleo, M.C., Cione, E., Castañera Gonzalez, R., Huang, G.C., and Persaud, S.J. (2016). Fine tuning of insulin secretion by release of nerve growth factor from mouse and human islet beta-cells. Mol. Cell. Endocrinol. *436*, 23–32. https://doi.org/10.1016/j.mce.2016.07.014.

47. Oslowski, C.M., Hara, T., O'Sullivan-Murphy, B., Kanekura, K., Lu, S., Hara, M., Ishigaki, S., Zhu, L.J., Hayashi, E., Hui, S.T., et al. (2012). Thioredoxin-interacting protein mediates ER stress-induced beta cell death through initiation of the inflammasome. Cell Metab. *16*, 265–273. https://doi.org/10.1016/j.cmet.2012.07.005.

48. Logunova, N., Kapina, M., Kondratieva, E., and Apt, A. (2023). The H2-A Class II molecule alpha/beta-chain cis-mismatch severely affects cell surface expression, selection of conventional CD4(+) T cells and protection against TB infection. Front. Immunol. *14*, 1183614. https://doi.org/10.3389/fimmu.2023.1183614.

49. Delong, T., Baker, R.L., Reisdorph, N., Reisdorph, R., Powell, R.L., Armstrong, M., Barbour, G., Bradley, B., and Haskins, K. (2011). Islet amyloid polypeptide is a target antigen for diabetogenic CD4+ T cells. Diabetes *60*, 2325–2330. https://doi.org/10.2337/db11-0288.

50. Yang, M.L., Kibbey, R.G., and Mamula, M.J. (2022). Biomarkers of autoimmunity and beta cell metabolism in type 1 diabetes. Front. Immunol. *13*, 1028130. https://doi.org/10.3389/fimmu.2022.1028130.

51. Lau, J., and Hebrok, M. (2010). Hedgehog signaling in pancreas epithelium regulates embryonic organ formation and adult beta-cell function. Diabetes *59*, 1211–1221. https://doi.org/10.2337/db09-0914.

52. Mozzarelli, A.M., Simanshu, D.K., and Castel, P. (2024). Functional and structural insights into RAS effector proteins. Mol. Cell *84*, 3163–3164. https://doi.org/10.1016/j.molcel.2024.07.024.

53. Jing, G., Jo, S., and Shalev, A. (2024). A novel class of oral, non-immunosuppressive, beta cell-targeting, TXNIP-inhibiting T1D drugs is emerging. Front. Endocrinol. *15*, 1476444. https://doi.org/10.3389/fendo.2024.1476444.

54. Burke, S.J., and Collier, J.J. (2015). Transcriptional regulation of chemokine genes: a link to pancreatic islet inflammation? Biomolecules *5*, 1020–1034. https://doi.org/10.3390/biom5021020.

55. Salem, H.H., Trojanowski, B., Fiedler, K., Maier, H.J., Schirmbeck, R., Wagner, M., Boehm, B.O., Wirth, T., and Baumann, B. (2014). Long-term IKK2/NF-kappaB signaling in pancreatic beta-cells induces immune-mediated diabetes. Diabetes *63*, 960–975. https://doi.org/10.2337/db13-1037.

56. Fuselier, T., Mota de Sa, P., Qadir, M.M.F., Xu, B., Allard, C., Meyers, M.M., Tiano, J.P., Yang, B.S., Gelfanov, V., Lindsey, S.H., et al. (2022). Efficacy of glucagon-like peptide-1 and estrogen dual agonist in pancreatic islets protection and pre-clinical models of insulin-deficient diabetes. Cell Rep. Med. *3*, 100598. https://doi.org/10.1016/j.xcrm.2022.100598.

57. Dooley, J., Tian, L., Schonefeldt, S., Delghingaro-Augusto, V., Garcia-Perez, J.E., Pasciuto, E., Di Marino, D., Carr, E.J., Oskolkov, N., Lyssenko, V., et al. (2016). Genetic predisposition for beta cell fragility underlies type 1 and type 2 diabetes. Nat. Genet. *48*, 519–527. https://doi.org/10.1038/ng.3531.

58. Liston, A., Todd, J.A., and Lagou, V. (2017). Beta-Cell Fragility As a Common Underlying Risk Factor in Type 1 and Type 2 Diabetes. Trends Mol. Med. *23*, 181–194. https://doi.org/10.1016/j.molmed.2016.12.005.

59. Singh, K., Bricard, O., Haughton, J., Björkqvist, M., Thorstensson, M., Luo, Z., Mascali, L., Pasciuto, E., Mathieu, C., Dooley, J., and Liston, A. (2022). Gene Delivery of Manf to Beta-Cells of the Pancreatic Islets Protects NOD Mice from Type 1 Diabetes Development. Biomolecules *12*, 1493. https://doi.org/10.3390/biom12101493.

60. Ong, G., and Logue, S.E. (2023). Unfolding the Interactions between Endoplasmic Reticulum Stress and Oxidative Stress. Antioxidants (Basel) *12*, 981. https://doi.org/10.3390/antiox12050981.

61. Cullinan, S.B., and Diehl, J.A. (2006). Coordination of ER and oxidative stress signaling: the PERK/Nrf2 signaling pathway. Int. J. Biochem. Cell Biol. *38*, 317–332. https://doi.org/10.1016/j.biocel.2005.09.018.

62. Hourihan, J.M., Moronetti Mazzeo, L.E., Fernández-Cárdenas, L.P., and Blackwell, T.K. (2016). Cysteine Sulfenylation Directs IRE-1 to Activate the SKN-1/Nrf2 Antioxidant Response. Mol. Cell *63*, 553–566. https://doi.org/10.1016/j.molcel.2016.07.019.

63. Li, C., Fang, Y., and Chen, Y.M. (2025). Beyond Redox Regulation: Novel Roles of TXNIP in the Pathogenesis and Therapeutic Targeting of Kidney Disease. Am. J. Pathol. *195*, 615–625. https://doi.org/10.1016/j.ajpath.2024.12.011.

64. Borowiec, A.M., Właszczuk, A., Olakowska, E., and Lewin-Kowalik, J. (2022). TXNIP inhibition in the treatment of diabetes. Verapamil as a novel therapeutic modality in diabetic patients. Med. Pharm. Rep. *95*, 243–250. https://doi.org/10.15386/mpr-2187.

65. Bolzán, A.D., and Bianchi, M.S. (2002). Genotoxicity of streptozotocin. Mutat. Res. *512*, 121–134. https://doi.org/10.1016/s1383-5742(02)00044-3.

66. Tonne, J.M., Sakuma, T., Deeds, M.C., Munoz-Gomez, M., Barry, M.A., Kudva, Y.C., and Ikeda, Y. (2013). Global gene expression profiling of pancreatic islets in mice during streptozotocin-induced beta-cell damage and pancreatic Glp-1 gene therapy. Dis. Model. Mech. *6*, 1236–1245. https://doi.org/10.1242/dmm.012591.

67. Lee, H., Sahin, G.S., Chen, C.W., Sonthalia, S., Cañas, S.M., Oktay, H.Z., Duckworth, A.T., Brawerman, G., Thompson, P.J., Hatzoglou, M., et al. (2023). Stress-induced beta cell early senescence confers protection against type 1 diabetes. Cell Metab. *35*, 2200–2215.e9. https://doi.org/10.1016/j.cmet.2023.10.014.

68. Varghese, S.S., and Dhawan, S. (2023). Senescence: a double-edged sword in beta-cell health and failure? Front. Endocrinol. *14*, 1196460. https://doi.org/10.3389/fendo.2023.1196460.

69. Li, Y., Liu, Y., Chiang, Y.J., Huang, F., Li, Y., Li, X., Ning, Y., Zhang, W., Deng, H., and Chen, Y.G. (2019). DNA Damage Activates TGF-beta Signaling via ATM-c-Cbl-Mediated Stabilization of the Type II Receptor TbetaRII. Cell Rep *28*, 735–745.e734. https://doi.org/10.1016/j.celrep.2019.06.045.

70. Hoshino, A., Ariyoshi, M., Okawa, Y., Kaimoto, S., Uchihashi, M., Fukai, K., Iwai-Kanai, E., Ikeda, K., Ueyama, T., Ogata, T., and Matoba, S. (2014). Inhibition of p53 preserves Parkin-mediated mitophagy and pancreatic beta-cell function in diabetes. Proc. Natl. Acad. Sci. USA *111*, 3116–3121. https://doi.org/10.1073/pnas.1318951111.

71. Kim, Y., Li, C., Gu, C., Fang, Y., Tycksen, E., Puri, A., Pietka, T.A., Sivapackiam, J., Kidd, K., Park, S.J., et al. (2023). MANF stimulates autophagy and restores mitochondrial homeostasis to treat autosomal dominant tubulointerstitial kidney disease in mice. Nat. Commun. *14*, 6493. https://doi.org/10.1038/s41467-023-42154-0.

72. Toren, E., Burnette, K.S., Banerjee, R.R., Hunter, C.S., and Tse, H.M. (2021). Partners in Crime: Beta-Cells and Autoimmune Responses Complicit in Type 1 Diabetes Pathogenesis. Front. Immunol. *12*, 756548. https://doi.org/10.3389/fimmu.2021.756548.

73. Quesada-Masachs, E., Zilberman, S., Rajendran, S., Chu, T., McArdle, S., Kiosses, W.B., Lee, J.H.M., Yesildag, B., Benkahla, M.A., Pawlowska, A., et al. (2022). Upregulation of HLA class II in pancreatic beta cells from organ donors with type 1 diabetes. Diabetologia *65*, 387–401. https://doi.org/10.1007/s00125-021-05619-9.

74. Koppinen, T.K., Reyes, C.R., Nam, J., Singh, A., Mandal, S., Beckett, L., Montedeoca, A., Kallionpää, T.A.E., Lindahl, M., Rivera, F.J., and Voutilainen, M.H. (2026). Modulation of the unfolded protein response with a C-terminal fragment of MANF facilitates recovery in models of multiple sclerosis. Mol. Ther. *34*, 1234–1257. https://doi.org/10.1016/j.ymthe.2025.10.023.

75. Mätlik, K., Yu, L.Y., Eesmaa, A., Hellman, M., Lindholm, P., Peränen, J., Galli, E., Anttila, J., Saarma, M., Permi, P., et al. (2015). Role of two sequence motifs of mesencephalic astrocyte-derived neurotrophic factor in its survival-promoting activity. Cell Death Dis. *6*, e2032. https://doi.org/10.1038/cddis.2015.371.

76. Politi, K., Zakowski, M.F., Fan, P.D., Schonfeld, E.A., Pao, W., and Varmus, H.E. (2006). Lung adenocarcinomas induced in mice by mutant EGF receptors found in human lung cancers respond to a tyrosine kinase inhibitor or to down-regulation of the receptors. Genes Dev. *20*, 1496–1510. https://doi.org/10.1101/gad.1417406.

77. Fisher, G.H., Wellen, S.L., Klimstra, D., Lenczowski, J.M., Tichelaar, J.W., Lizak, M.J., Whitsett, J.A., Koretsky, A., and Varmus, H.E. (2001). Induction and apoptotic regression of lung adenocarcinomas by regulation of a K-Ras transgene in the presence and absence of tumor suppressor genes. Genes Dev. *15*, 3249–3262. https://doi.org/10.1101/gad.947701.

78. Nir, T., Melton, D.A., and Dor, Y. (2007). Recovery from diabetes in mice by beta cell regeneration. J. Clin. Invest. *117*, 2553–2561. https://doi.org/10.1172/JCI32959.

79. Hakonen, E., Ustinov, J., Eizirik, D.L., Sariola, H., Miettinen, P.J., and Otonkoski, T. (2014). In vivo activation of the PI3K-Akt pathway in mouse beta cells by the EGFR mutation L858R protects against diabetes. Diabetologia *57*, 970–979. https://doi.org/10.1007/s00125-014-3175-2.

80. Galli, E., Härkönen, T., Sainio, M.T., Ustav, M., Toots, U., Urtti, A., Yliperttula, M., Lindahl, M., Knip, M., Saarma, M., and Lindholm, P. (2016). Increased circulating concentrations of mesencephalic astrocyte-derived neurotrophic factor in children with type 1 diabetes. Sci. Rep. *6*, 29058. https://doi.org/10.1038/srep29058.

81. Galaxy Community (2022). The Galaxy platform for accessible, reproducible and collaborative biomedical analyses: 2022 update. Nucleic Acids Res. *50*, W345–W351. https://doi.org/10.1093/nar/gkac247.

82. Koopmans, F., van Nierop, P., Andres-Alonso, M., Byrnes, A., Cijsouw, T., Coba, M.P., Cornelisse, L.N., Farrell, R.J., Goldschmidt, H.L., Howrigan, D.P., et al. (2019). SynGO: An Evidence-Based, Expert-Curated Knowledge Base for the Synapse. Neuron *103*, 217–234.e4. https://doi.org/10.1016/j.neuron.2019.05.002.

83. Huang, D.W., Sherman, B.T., and Lempicki, R.A. (2009). Systematic and integrative analysis of large gene lists using DAVID bioinformatics resources. Nat. Protoc. *4*, 44–57. https://doi.org/10.1038/nprot.2008.211.

84. Sherman, B.T., Hao, M., Qiu, J., Jiao, X., Baseler, M.W., Lane, H.C., Imamichi, T., and Chang, W. (2022). DAVID: a web server for functional enrichment analysis and functional annotation of gene lists (2021 update). Nucleic Acids Res. *50*, W216–W221. https://doi.org/10.1093/nar/gkac194.

YMTHE, Volume *34*

Supplemental Information

Elevated MANF expression in β cells protects mice from streptozotocin-induced diabetes by attenuating islet stress and immunogenicity

Huini Li, Tatiana Danilova, Erik Palm, Emmi Pakarinen, Julia Kiva, Tõnis Org, Tapani K. Koppinen, Elina Hakonen, Indrek Teino, Merja H. Voutilainen, Timo Otonkoski, and Maria Lindahl

Supplemental Text

Characterization of islet early gene responses in the prediabetic MLDS model by RNA-seq

Despite the extensive use of the multiple low doses of streptozotocin (MLDS) model, the early effects of streptozotocin (STZ) treatment on the global gene expression profile in islets have remained obscure. To investigate the direct effects of STZ on islet gene expression during the early stages of diabetes, we performed bulk RNA-seq analysis on isolated islets from MANF-overexpressing or control mice administered with low-doses of STZ or vehicle (buffer). Islets were harvested on day 4 post-injection to study early transcriptional changes prior to overt β cell loss. We used STZ (S0130, Sigma-Aldrich), which proved to be highly effective in inducing β cell death and hyperglycemia within 3 weeks after the first STZ injection.[1] We reasoned that transcriptomics analyses of islets from prediabetic STZ-injected transgenic mice *in vivo* would reveal the physiological interplay between injured islet β cells and the immune system.

The effect of STZ alone was compared by transcriptional differences between islets from vehicle- and STZ-treated INS-MANF DOX– control mice. Differentially expressed genes (DEGs) were identified using DESeq2, revealing 643 genes with an absolute Log2 fold change ($|Log_2FC|$) greater than 0.5 and FDR less than 0.05 (Table S1), with 322 genes upregulated and 321 genes downregulated in response to STZ.

A heatmap was generated to highlight the expression levels of key islet genes affected by STZ alone, selected from the 643 DEGs (Figure 5C). We first confirmed that the expression of most genes critical for insulin production, processing, secretion and glucose sensing, were significantly reduced by STZ treatment, suggesting a decline in insulin production and compromised β cell function. However, the expression of genes essential for adult β cell identity and maintenance, including *Pdx1*, *Neurod1, Ucn3, Nkx6.1, Gck, Pax6* as well as progenitor cell markers, *Sox9, Hes1, Ngn3, Aldh1a3, Serpina7* did not differ significantly between groups, indicating that β cells remained present and retained their identity in our STZ model. These results suggest that our prediabetic MLDS model resembles an

early disease stage, with β cell dysfunction but not yet a complete loss of β cell identity. Conversely, similar studies have shown that at five to seven days after the first MLDS injection most of the β cell markers are significantly downregulated with vast β cell dysfunction and death.[2]

Gene Ontology (GO) enrichment analyses of 322 upregulated DEGs by STZ using DAVID tool revealed that biological processes (BP) regulating inflammatory and innate immune responses, chemokine-mediated signaling, tumor necrosis factor production, reactive oxygen species metabolic processes, DNA damage response (DDR), and intrinsic apoptotic signaling in response to DNA damage by p53 class mediator, were among the top enriched terms (Figure 5A and Table S1). In addition, KEGG pathway analyses of the 322 upregulated DEGs by STZ revealed that the p53 signaling pathway was the most enriched (Figure S7E and Table S1). STZ directly leads to DNA Alkylation damage.[3] DNA damage response genes are clearly increased in the islets of our STZ-induced diabetic mouse model (*Gadd45a, Parp3, Ddias, Zc3h12a, Pierce1*) as well as p53-mediated cell-cycle arrest and apoptosis genes (*Tnfrs10b, Bax, Bcl3, Casp7, Bbc3 (PUMA), Ccng1, Trp53inp1, Trp53cor1, Wee1, Zmat3, Mdm2*). These features are shared with increased replicative senescence (*Cdkn1a*). Persistent DDR leads to chromatin reorganization (*H2aj, H4c8, H1f3*) and acquisition of a secretome composed of cytokines (*Il6*, $Log_2(FC) < 0.5$), chemokines (*Ccl2, Cxcl1*), growth factors (*Igfbp3*), extracellular matrix factors (*Mmp12*) and other senescence-associated secretory phenotype (SASP) factors (*Serpine1*), and p21-associated secretory phenotype (PASP) factors (*Csf1, Ctsd*).[4] Conversely, GO analysis of 321 downregulated genes by STZ highlighted the top BPs involving positive regulation of gene expression, cell adhesion, extracellular matrix organization, intracellular calcium ion homeostasis, and monoatomic cation transmembrane transport (Figure 5B and Table S1). KEGG pathway analyses further revealed the biosynthesis of amino acids as the top downregulated pathway (Figure S7F and Table S1). In addition, oxidative stress response (*Dcxr, Hmox1, Nfe2l2*) was highly upregulated in response to STZ treatment (Figure 5C), while the expression of UPR genes was not significantly altered at this early stage. Thus, STZ-induced DNA alkylation damage triggers

a robust p53-mediated DNA damage response in β cells, driving cell cycle arrest, apoptosis, and the establishment of a potent senescence-associated inflammatory phenotype characterized by SASP/PASP factor expression. Concurrently, a significant reduction of gene expression and amino acid biosynthesis drives progressive β cell dysfunction and decline in our prediabetic MLDS model. Furthermore, despite the initial DNA damage, β cell dysfunction and p53-mediated cell death, immune response was the most significantly enriched GO pathway from the upregulated genes (Figure 5A and Table S1). The heatmap showed that the expression of genes involved in immune system processes (*Cd84, Jak3, Il4ra, Tap1*), inflammatory response (*Il1rn, Tlr13, Tlr8, Stat3*) and chemokine-mediated signaling pathways (*Ccl2, Cxcl1, Ccr1, Ccl9*) were highly upregulated in response to STZ treatment (Figure 5C). These upregulated genes may be derived from intrinsic β cell inflammation, SASP and invading immune cells. Overall, these results suggest that the prediabetic MLDS model resembles key initiating events of β cell dysfunction, destruction and local immune response associated with T1D β cell pathology as previously reported.[1,5,6]

Supplemental Figures

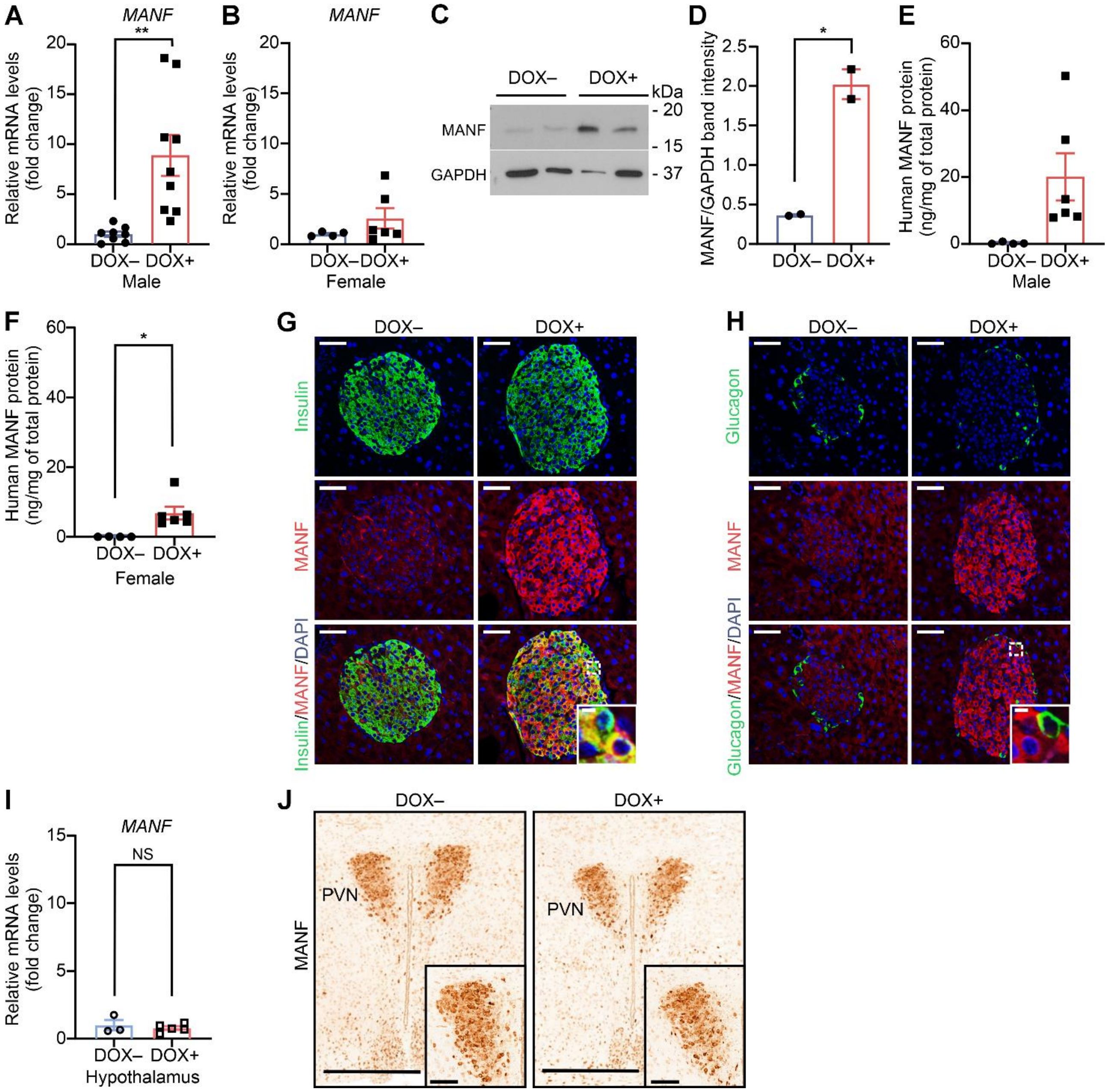

Figure S1. MANF is overexpressed in insulin-positive β cells, but not in glucagon-positive α cells nor the hypothalamic regions of male INS-MANF DOX+ mice. (**A**) A 8.9-fold increase in human *MANF* mRNA levels in β cells of islets from MANF-overexpressing (DOX+, *n* = 9) male mice compared to INS-MANF DOX– (DOX–, *n* = 8) islets, quantified by RT-qPCR. (**B**) Islets isolated from female DOX+ mice express less transgenic human *MANF* (*n* = 6) compared to male islets but 2.6-fold more than control DOX– (*n* = 4) female islets. (**C** and **D**) Representative Western blot showing a 4-fold increase in MANF protein levels in CHO cells transfected with TET-MANF construct and a PB-CA-rtTA-On vector in the presence and absence of

doxycycline (n = 2 per group). (**E** and **F**) Human MANF levels measured by human-specific MANF ELISA in the DOX+ islets from (**E**) male (n = 6) and (**F**) female (n = 6) mice compared to DOX– male (**E**, n = 4) and female mice (**F**, n = 4). (**G** and **H**) Transgenic MANF is expressed in β cells (**G**, yellow) but not in glucagon-positive α cells (**H**, green) in the islets of DOX+ mice shown by immunofluorescence staining of pancreas. Nuclei with DAPI (blue). Scale bar, 50 μm, 5 μm (insets). (**I**) Human *MANF* mRNA expression was not increased in punches dissected from hypothalamic regions of male mice (DOX+, n = 5; DOX–, n = 3). (**J**) MANF immunohistochemistry on brain sections showed no difference in intensity of staining between DOX+ and DOX– mice. Scale bar, 500 μm, 100 μm (insets). Data are expressed as mean ± SEM, and scatter plots show all individual data points. Statistical analyses were performed using Student's two-tailed unpaired *t*-tests. *$p < 0.05$, **$p < 0.01$, NS > 0.05.

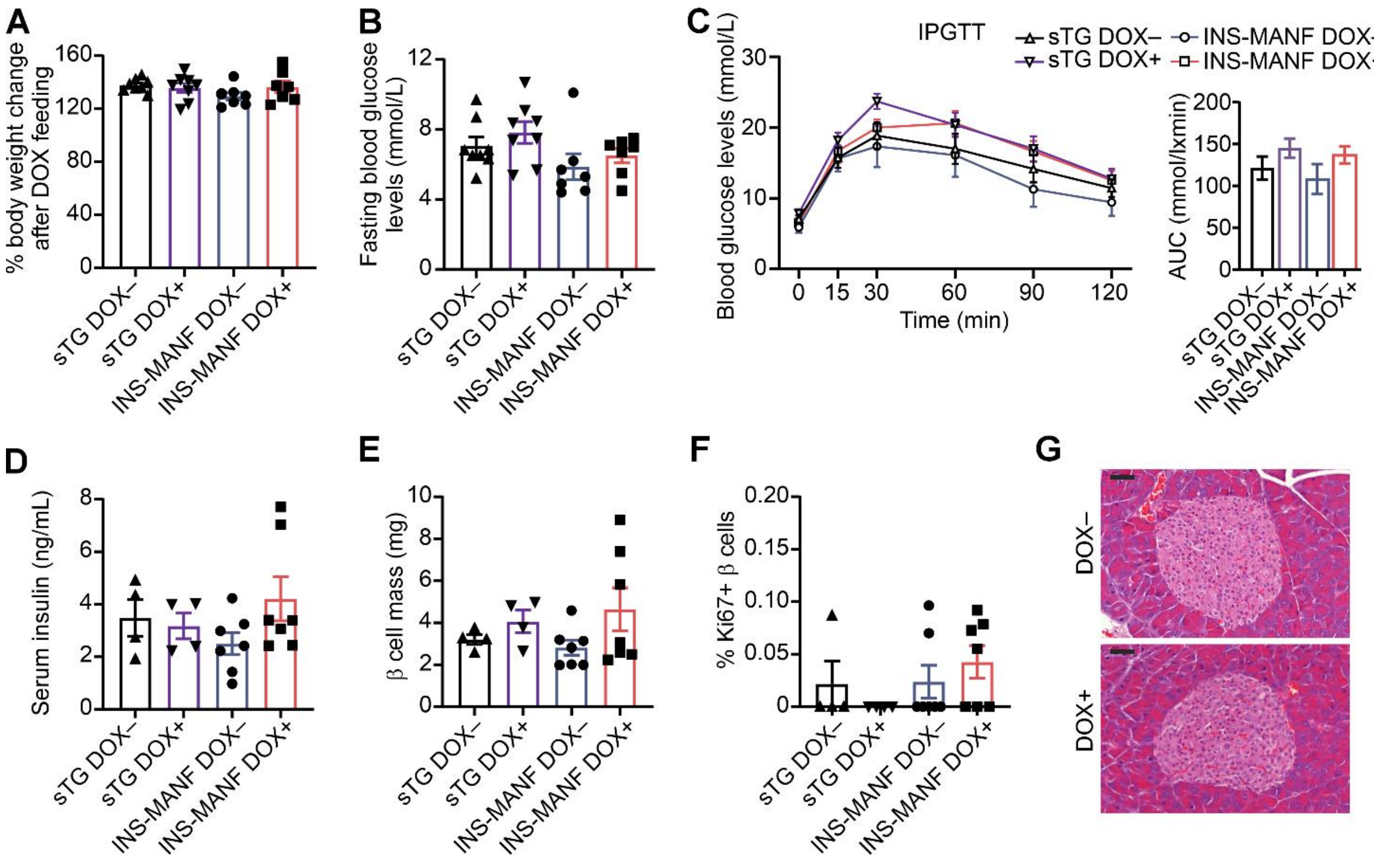

Figure S2. Prolonged MANF overexpression does not lead to significant changes in β cell proliferation nor β cell mass in male INS-MANF DOX+ mice. (**A**) No difference in the body weight change (%) between INS-MANF male mice on doxycycline (DOX) chow for 5 months (INS-MANF DOX+, *n* = 7) compared to INS-MANF mice on normal chow (INS-MANF DOX–, *n* = 7), and control single-transgenic (sTG) mice, on normal (sTG DOX–, *n* = 8), and DOX chow (sTG DOX+, *n* = 8). (**B**) No significant difference in fasting blood glucose levels. (**C**) Intraperitoneal glucose tolerance test (IPGTT) and area under the curve (AUC) of the mean blood glucose levels for each group revealed no significant difference in glucose clearing. (**D**) A trend for increased serum insulin levels in male INS-MANF DOX+ mice (*n* = 7), compared to control mice (sTG DOX–, *n* = 4; sTG DOX+, *n* = 4; INS-MANF DOX–, *n* = 7). (**E** and **F**) Quantification of the β cell mass from insulin antibody-stained sections after 5 months DOX-feeding revealed a trend for increased β cell mass (**E**) and β cell proliferation (**F**) in INS-MANF DOX+ male mice, compared to control mice. (**G**) Hematoxylin-eosin-stained pancreas sections showed no obvious pathological changes in pancreases of INS-MANF mice fed for one year with DOX-containing chow, compared to control. Scale bar, 50 μm. Data are expressed as mean ± SEM, and scatter plots show all individual data points. Statistical analyses were performed using one-way

ANOVA followed by Tukey's post hoc test for (**A**), (**B**), AUC in (**C**), (**D**–**F**) and two-way ANOVA with Tukey's post hoc test for different time points in (**C**).

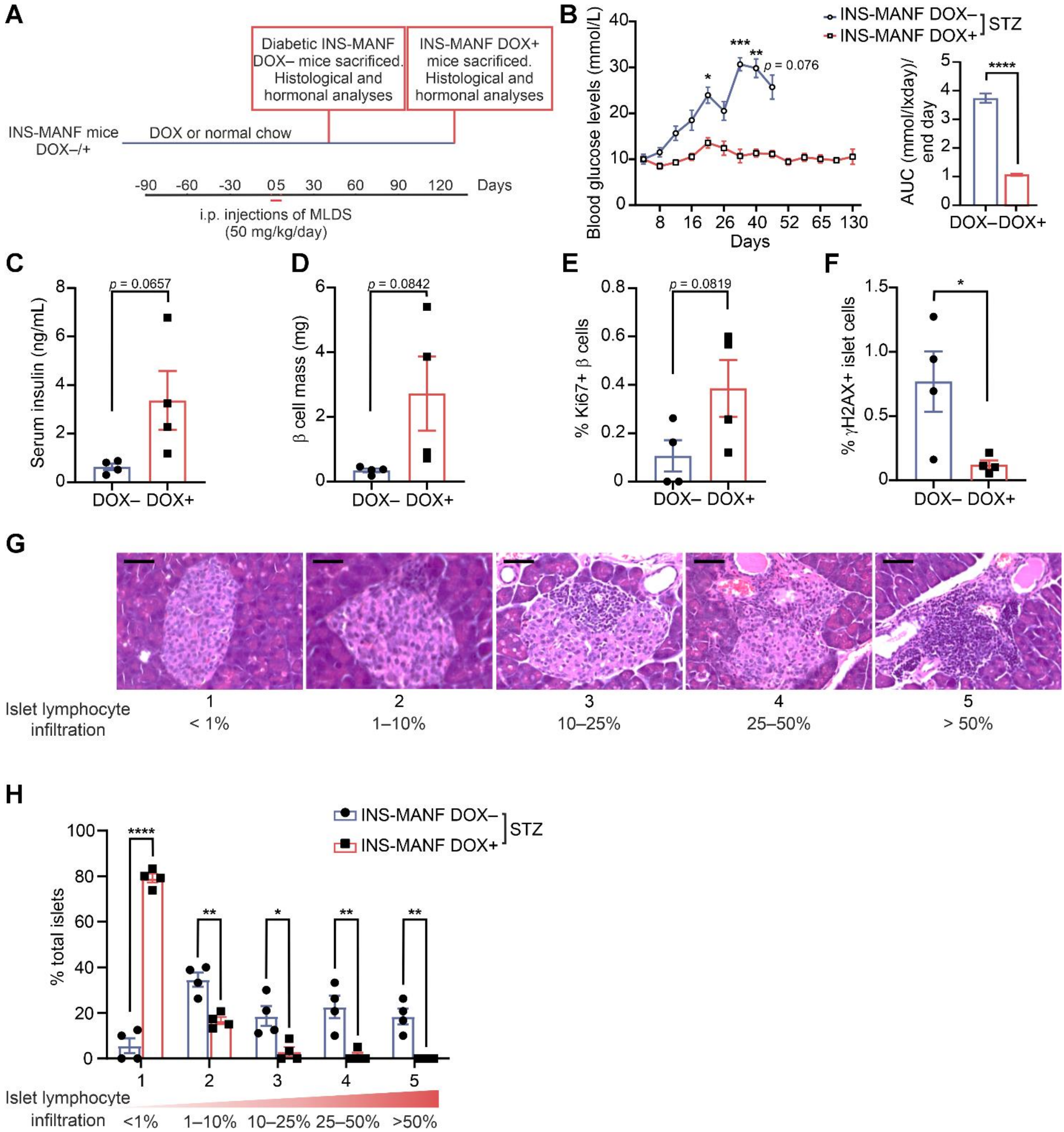

Figure S3. MANF overexpression in β cells restores normoglycemia over the long term associated with reduced insulitis. (**A**) Timeline for the doxycycline (DOX) chow-induced MANF overexpression in β cells 90 days before the multiple low dose STZ injections (MLDS, 50 mg/kg/day) followed by 129 days of DOX+ feeding of INS-MANF mice (DOX+, $n = 4$). Control INS-MANF DOX– mice (DOX–, $n = 4$) were sacrificed 45 days after the first STZ injection. (**B**) Transient small increase in blood glucose levels of DOX+ mice after

MLDS returned to normal about 50 days after the first STZ injection and area under the curve (AUC) of the mean blood glucose levels for each group. (**C**) *Ad libitum* serum insulin levels and (**D**) β cell mass in MLDS injected DOX+ compared to DOX– mice. The lack of significance might be due to variable levels of MANF-overexpression in DOX+ mice. (**E**) A trend for increased β cell proliferation quantified from Ki67 and insulin antibody-stained pancreas sections in MLDS injected DOX+ compared to DOX– mice. (**F**) Significant less γH2AX positive islet cells in pancreases from MLDS injected DOX+ compared to DOX– mice. (**G**) Representative pictures of hematoxylin-eosin-stained pancreas sections showing different grades of islet lymphocyte infiltration. Scale bar, 50 µm. (**H**) Islet insulitis scores in hyperglycemic DOX– and DOX+ mice. Data expressed as mean ± SEM, and scatter plots show all individual data points. Statistical analyses were performed using a two-way ANOVA followed by Bonferroni's post hoc test for different time points in (**B**), Student's two-tailed unpaired *t*-tests for AUC in (**B**), (**C**–**F**) and different scores for (**H**). *$p < 0.05$, **$p < 0.01$, ***$p < 0.001$, ****$p < 0.0001$.

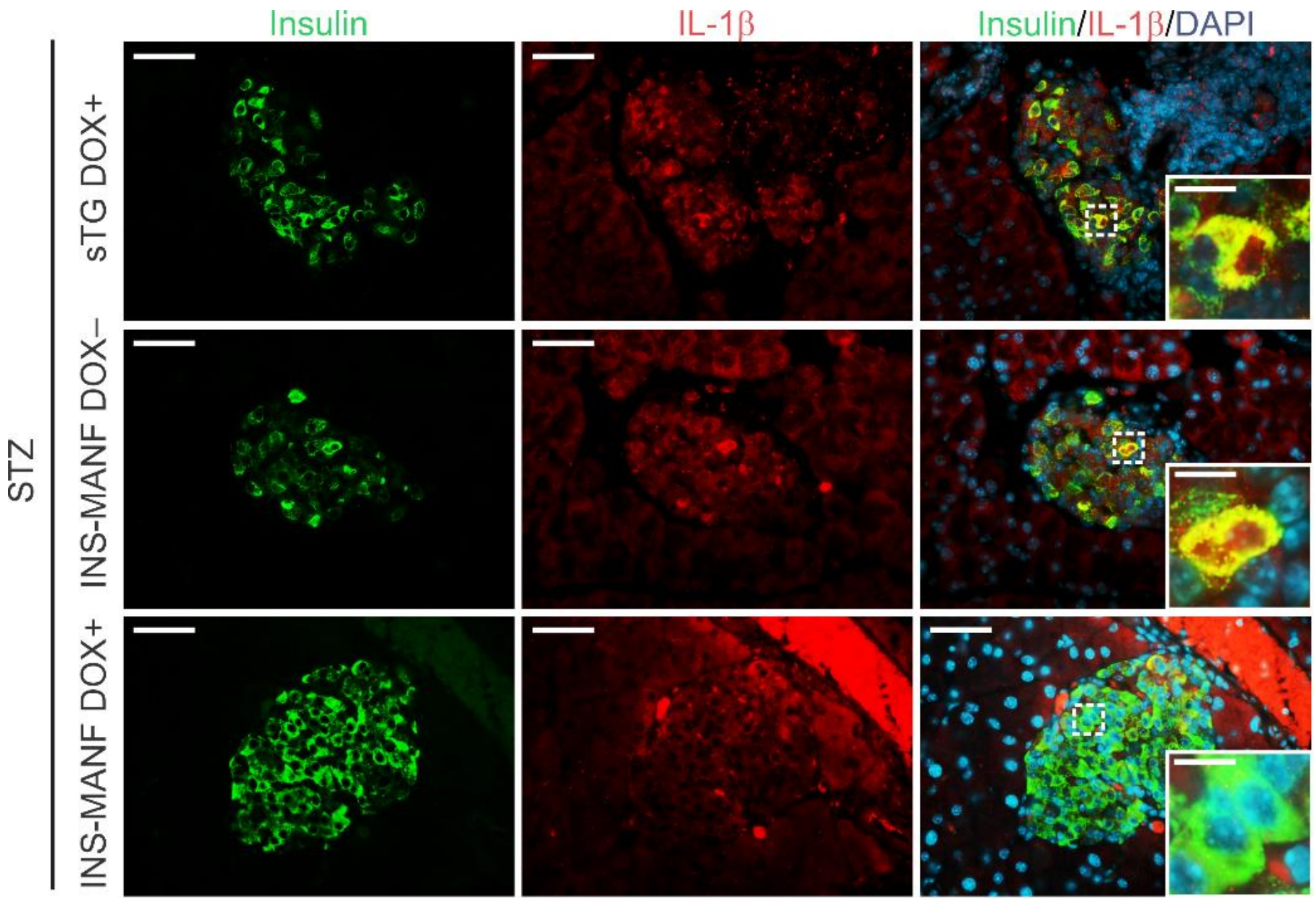

Figure S4. IL-1β staining of pancreatic sections from MLDS injected MANF-overexpressing mice compared to control mice. Representative images of IL-1β (red) and insulin (green) staining from MANF overexpressing and control mice in the MLDS model. Scale bar, 50 μm, 10 μm (insets).

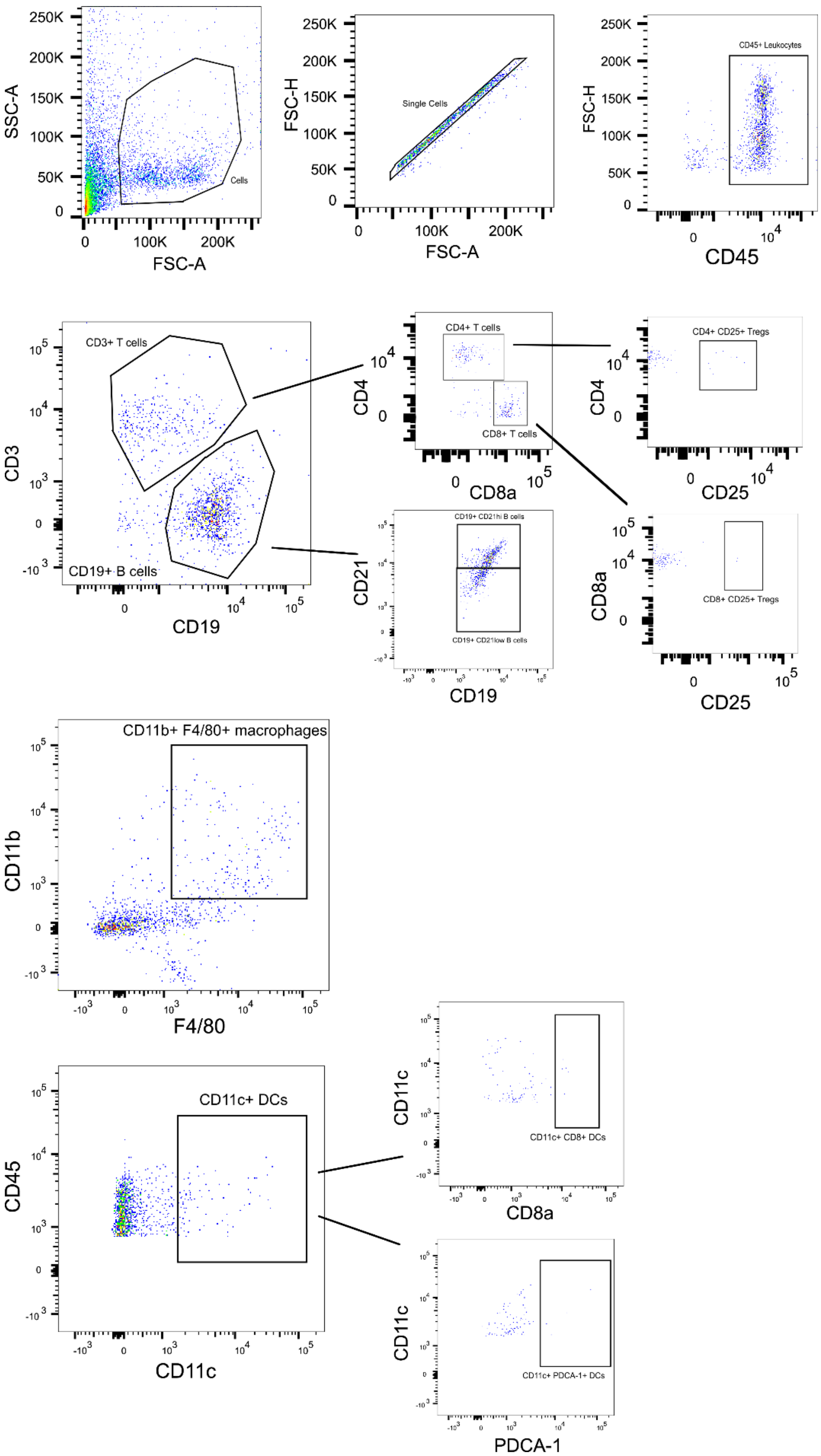

Figure S5. Flow cytometry gating strategy for immunophenotyping analysis.

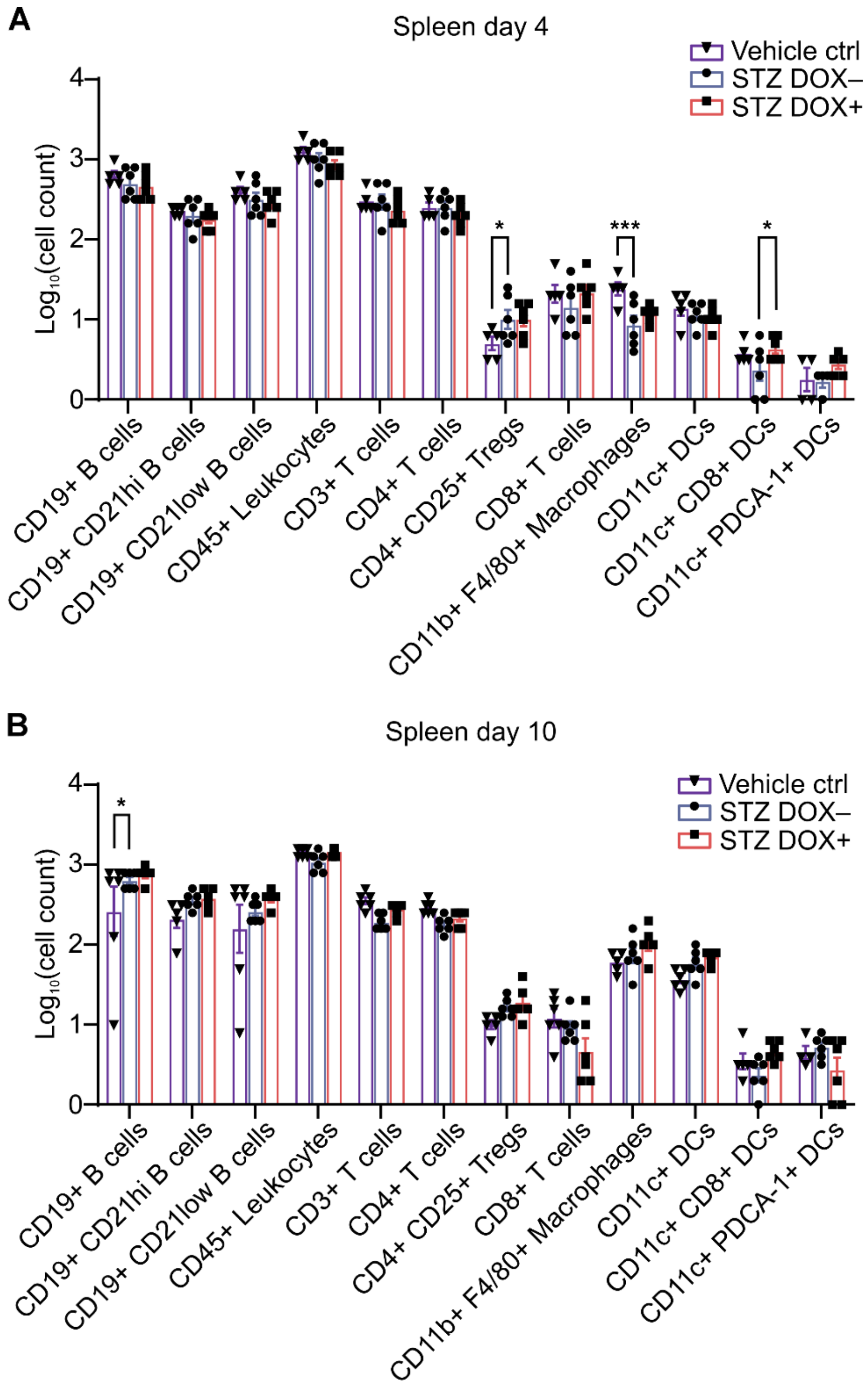

Figure S6. Immunophenotyping analysis of spleen tissue. (**A** and **B**) Immune cell profiling of spleens on day 4 (**A**) and 10 (**B**) after the first STZ (45mg/kg/day) or vehicle injection. Treatment groups include non-diabetic control mice injected with vehicle (Vehicle ctrl, n = 5), triangles, purple bar including TET-MANF DOX– (n = 1), INSrt-TA DOX– (n = 2) and INS-MANF DOX– (n = 2) mice treated with STZ (STZ DOX–, n = 6), circles, blue bar and INS-MANF DOX+ mice treated with STZ (STZ DOX+, n = 6), squares, red bar. Data expressed as mean ± SEM, and scatter plots show all individual data points. Statistical analyses were

performed using two-way ANOVA followed by Holm-Sidak's multiple comparison test. $^{*}p < 0.05$, $^{***}p < 0.001$.

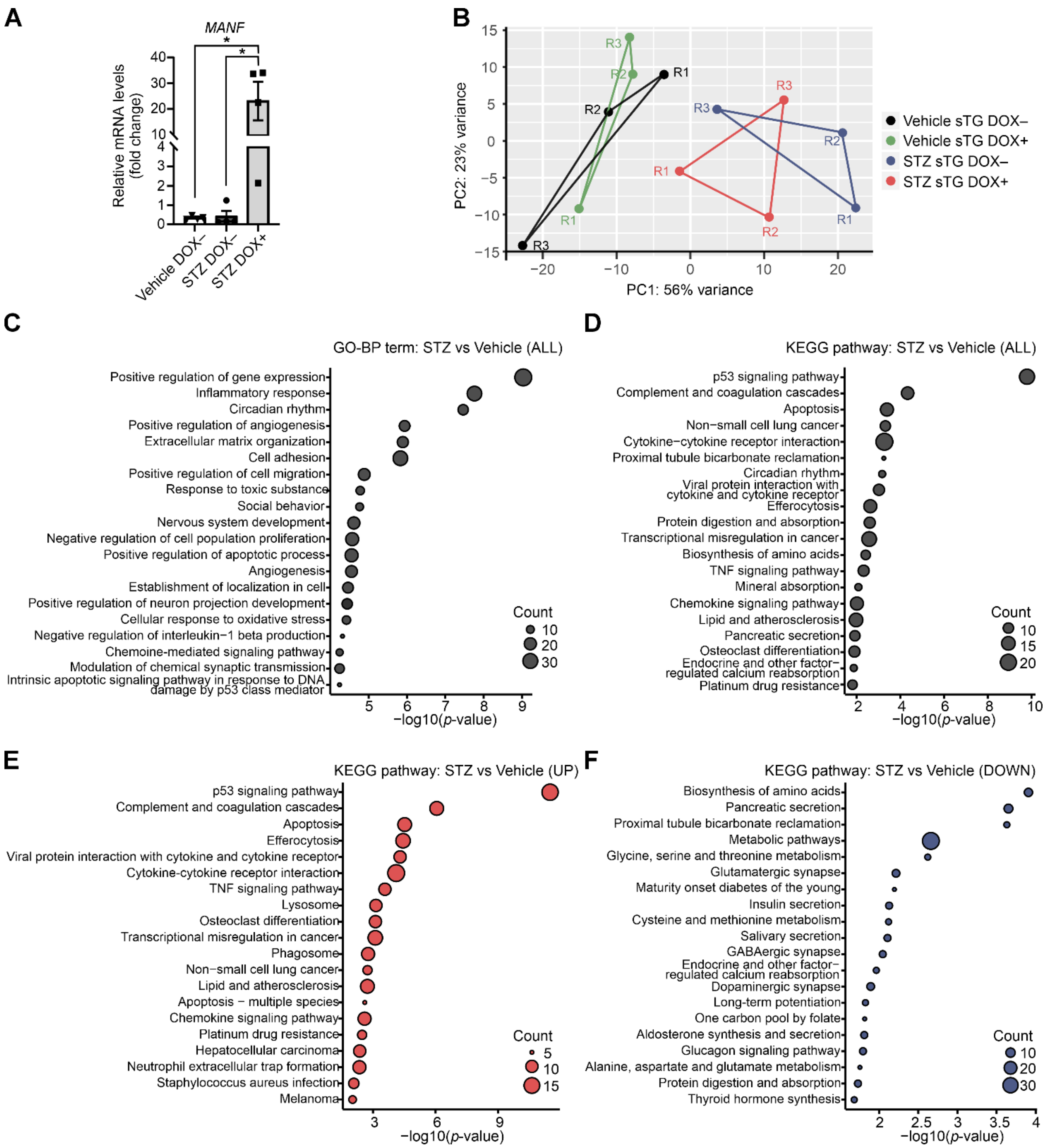

Figure S7. GO-BP terms and KEGG pathway enrichment analysis of differentially expressed genes in islets between STZ- and vehicle-injected INS-MANF DOX– control mice. (**A**) The mRNA levels of human *MANF* in the pancreatic islets of vehicle and STZ-injected INS-MANF DOX+/– mice used in RNA-seq (n = 4 per group), quantified by RT-qPCR. (**B**) PCA of all 12 sTG samples with 3 replicates (R1, R2, R3) in each 4 groups either treated with STZ or vehicle, fed by DOX+ or normal chow (DOX–) to represent the distribution

and relationships of samples in a high-dimensional space reduced to two principal components, PC1 and PC2 ($n = 3$ per group). No major changes in the overall gene expression in islets from mice fed by doxycycline diet compared to controls in the RNA-seq data. Each treatment group is presented in different color indicated in the figure. (**C**) Bubble diagrams of the GO enrichment analyses for BP terms by DAVID, on islet mRNA using 643 significantly altered DEGs with |Log2(FC)| > 0.5 and FDR < 0.05 on the effect of STZ in INS-MANF DOX– islets (STZ DOX–, $n = 4$) compared to islets from buffer treated mice (Vehicle DOX–, $n = 4$). (**D–F**) KEGG pathways analyses with all 643 altered DEGs (**D**), 322 significantly upregulated DEGs (**E**) and 321 downregulated DEGs (**F**) with $|Log_2(FC)| > 0.5$ and FDR < 0.05 by STZ alone between Vehicle DOX– (n = 4) and STZ DOX– ($n = 4$), analyzed by DAVID. Data expressed as mean ± SEM, and scatter plots show all individual data points. Statistical analyses were performed using one-way ANOVA followed by Tukey's post hoc test for (**A**). *$p < 0.05$.

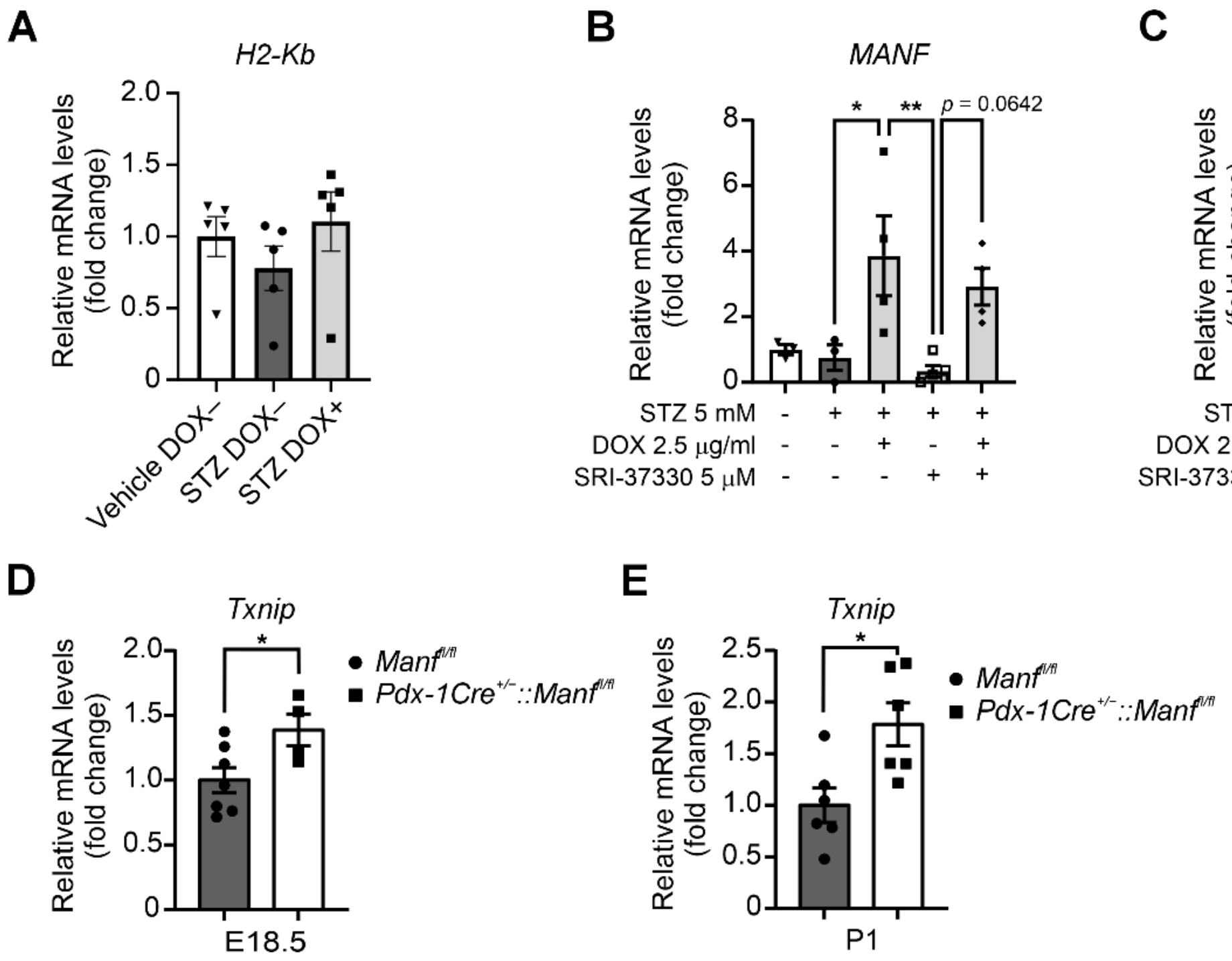

Figure S8. TXNIP mRNA levels are already increased in the pancreas and islets of embryonic and newborn MANF-deficient mice. (**A**) Relative mRNA levels of the MHC I molecule *H2-Kb*, measured by RT-qPCR in islets isolated from vehicle or MLDS-injected INS-MANF DOX+/– mice on day 4 after the first MLDS injection (*n* = 5 per group). (**B** and **C**) Relative mRNA levels of *MANF* (**B**) and *Txnip* (**C**) after STZ, DOX and TXNIP inhibitor SRI-37330 treatment (*n* = 3–5 per group). (**D**) *Txnip* mRNA expression in the E18.5 pancreas ($Manf^{fl/fl}$, *n* = 7; $Pdx\text{-}1Cre^{+/-}::Manf^{fl/fl}$, *n* = 4) and (**E**) in islets of newborn $Manf^{fl/fl}$ (*n* = 6) and pancreas-specific $Pdx\text{-}1Cre^{+/-}::Manf^{fl/fl}$ (*n* = 6) mice. Data expressed as mean ± SEM, and scatter plots show all individual data points. Statistical analyses were performed using one-way ANOVA followed by Tukey's post hoc test for (**A**–**C**). Student's two-tailed unpaired *t*-tests for (**D**) and (**E**). *$p < 0.05$, **$p < 0.01$.

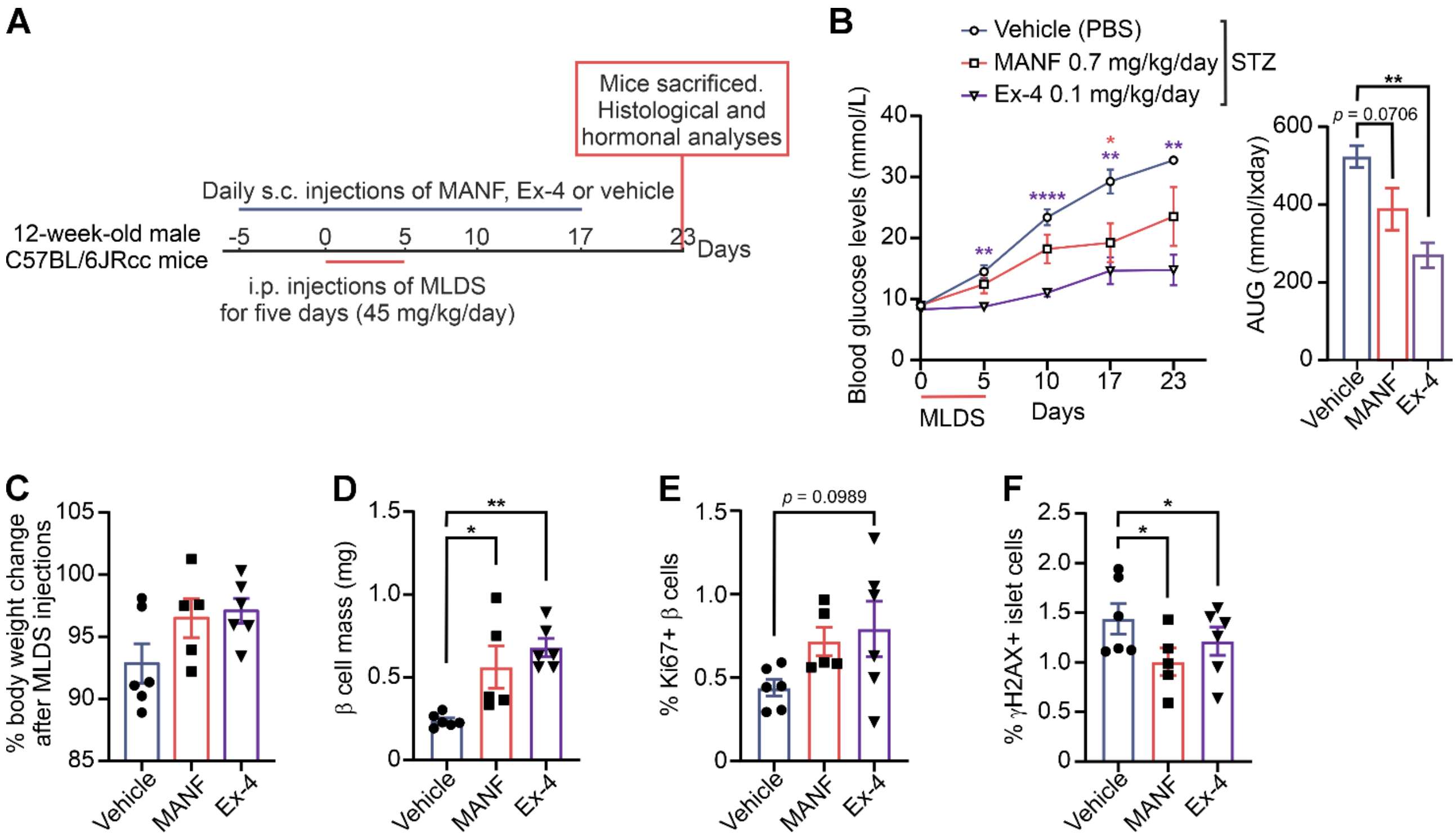

Figure S9. Systemic delivery of recombinant human MANF protein protects against MLDS-induced β cell loss *in vivo*. (**A**) Schematic illustration of the daily subcutaneous (s.c.) injections of MANF (0.7 mg/kg/day), Exendin-4 (Ex-4, 0.1 mg/kg/day) or vehicle (PBS) administered to 12-week-old male C57BL/6 mice starting 5 days before the first STZ intraperitoneal (i.p.) injection (45 mg/kg/day) and continuing until 23 days after the first injection ($n = 6$ per group). (**B**) *Ad libitum* fed blood glucose levels of MLDS-injected mice with vehicle, MANF or Ex-4 daily injections and area under the curve (AUC) of the mean blood glucose levels for each group. (**C**) Percentage in body weight change at the time of sacrifice compared to the body weight before the first STZ injection (vehicle, $n = 6$; MANF, $n = 5$, one mouse died due to an injection error at day 16; Ex-4, $n = 6$). (**D**) β cell mass quantified from insulin-stained pancreas sections of mice sacrificed 23 days after first STZ injection. (**E**) β cell proliferation quantified from insulin and Ki67 antibody–stained sections in STZ-treated mice. (**F**) Islet cell death assessed from anti-γH2AX antibody-stained pancreas sections of STZ-injected mice. Data expressed as mean ± SEM, and scatter plots show all individual data points. Statistical analyses were performed using two-way ANOVA with Holm-Sidak's multiple comparison test for the different time points in (**B**) and one-way ANOVA with Tukey's post hoc test for AUC in (**B**), (**C–F**). *$p < 0.05$, **$p < 0.01$. ****$p < 0.0001$.

Supplemental Tables

Table S1. Lists of differentially expressed genes, along with GO enrichment and KEGG pathway analyses, for the comparisons shown in Figure 5.

Available as a separate Excel file.

Table S2. Lists of differentially expressed genes, along with GO enrichment and KEGG pathway analyses, for the comparisons shown in Figure 6.

Available as a separate Excel file.

Table S3. List of antibodies.

Target	Catalog No.	Host Species	Clonality	Species Reactivity	Company	Application
MANF	HPA011175	Rabbit	Polyclonal	Human	Sigma-Aldrich	IF, IHC 1:200
TXNIP	ab188865	Rabbit	Monoclonal	Mouse, Rat, Human	Abcam	IF 1:400
GLUT2	sc-7580	Goat	Polyclonal	Mouse, Rat, Human	Santa Cruz	IF 1:200
Insulin	I2018	Mouse	Monoclonal	Mouse, Rat, Human	Sigma-Aldrich	IHC, ICC 1:1000
Insulin	ab181547	Rabbit	Monoclonal	Mouse, Rat, Human	Abcam	IF 1:500
Insulin	A0564	Guinea pig	Polyclonal	Mouse, Rat, Human	Dako	IF, IHC 1:3000
Glucagon	G2654	Mouse	Monoclonal	Feline, Canine, Mouse, Guinea pig, Rat, Rabbit, Human, Pig	Sigma-Aldrich	IF, IHC 1:500
CD45	ab10558	Rabbit	Polyclonal	Mouse, Rat, Human	Abcam	IF 1:200
IL-1β	ab9722	Rabbit	Polyclonal	Mouse, Human	Abcam	IF 1:100
GAPDH	MAB374	Mouse	Monoclonal	Canine, Human, Mouse, Rat, Rabbit, Fish, Feline, Pig	Sigma-Aldrich	WB 1:500
Ki67 (Clone SP6)	RM-9106	Rabbit	Monoclonal	Mouse, Human	Thermo Fisher Scientific	IF 1:200
Phospho-Histone γH2A.X (Ser139) (20E3)	9718	Rabbit	Monoclonal	Mouse, Rat, Human, Monkey	Cell Signaling Technology	IHC, ICC 1:400
MANF	310-100, Batch A12ManfPab-005, 0.45 mg/ml	Rabbit	Polyclonal	Human, Mouse	Icosagen	WB 1:500

Table S4. List of primers used for RT-qPCR.

Gene	Species	Forward primer	Reverse primer
MANF	human	GGC AAA GAG AAT CGG TTG TG	CAG ATC TTC TCC ACA GGG ATG
Manf	mouse	GAC AGC CAG ATC TGT GAA CTA AAA	TTT CAC CCG GAG CTT CTT C
Actin	mouse	CTA AGG CCA ACC GTG AAA AG	ACC AGA GAC ATA CAG GGA
Ddit3	mouse	CCA ACA GAG GTC ACA CGC AC	TGA CTG GAA TCT GGA GAG CGA
Atf4	mouse	ATG GCC GGC TAT GGA TGA T	CGA AGT CAA ACT CTT TCA GAT CCA TT
Atf5	mouse	AGG TGT ATA AGG CCC GAA GC	AGT GTG ACG CTG GAG ACA GA
Txnip	mouse	TCA AGG GCC CCT GGG AAC ATC	GAC ACT GGT GCC ATT AAG TCA G
Glut2	mouse	TGA GCA GAA GGT CTC CGT GA	TGT CGG TAA TTG GCA TCC GT
Ins1/2	mouse	AGC ATC TTT GTG GTC CCC AC	CCC CAC ACA CCA GGT AAA GAG
Pdx1	mouse	GAA ATC CAC CAA AGC TCA CG	CGG GTT CCG CTG TGT AAG
Ccl2	mouse	GCT GGA GCA TCC ACG TGT T	ATC TTG CTG GTG AAT GAG TA
Tnfa	mouse	GGA ACT GGC AGA AGA GGC ACT C	GCA GGA ATG AGA AGA GGC TGA GAC
Il6	mouse	TAG TCC TTC CTA CCC CAA TTT CC	TTG GTC CTT AGC CAC TCC TTC
Cxcl10	mouse	TCC TTG TCC TCC CTA GCT CA	ATA ACC CCT TGG GAA GAT GG
Nrf2	mouse	CAT GAT GGA CTT GGA GTT GC	CCT CCA AAG GAT GTC AAT CAA
Ccng1	mouse	GCG TTG GAG ATC CAA GCA CTG A	GGA AAC AAG CTC TTG CCA GAA GG
Trp53inp1	mouse	CTT CTC CTG TTT ACC TGC ATC TT	TGA TAG TGG TTA ATC CAC CTG CT
Cd48	mouse	GTA CAA AGA CAA TCT TCG AGT C	GCA CTC TCA TGT AGT AGG TAC
Cd84	mouse	ATA TAG CTG GAG TCC CTT TGG AG	AAA GAG CAC GGC CAA TCC TC
H2-Aa	mouse	CAA CCG TGA CTA TTC CTT CC	CCA CAG TCT CTG TCA GCT C
H2-Kb	mouse	GTG ATC TCT GGC TGT GAA GT	GTC TCC ACA AGC TCC ATG TC
Tgfb1	mouse	TGG AGC AAC ATG TGG AAC TC	CAG CAG CCG GTT ACC AAG
sXbp1	mouse	GAG TCC GCA GCA GGT G	GTG TCA GAG TCC ATG GGA
Trp53	mouse	CGC GGG CGT AAA CGC TTC GA	TCT TCA GGT AGC TGG AGT GAG CCC
Cdkn1a	mouse	TCG CTG TCT TGC ACT CTG GTG T	CCA ATC TGC GCT TGG AGT GAT AG
Trib3	mouse	CGC TTT GTC TTC AGC AAC TGT	TCA TCT GAT CCA GTC ATC ACG

Table S5. List of antibodies used for flow cytometry.

Target	Fluorophore	Isotype	Cell type	Catalog No.	Manifacturer
Zombie NIR	Zombie NIR		dead/alive cells	423106	Biolegends
CD45	FITC	Mouse IgG1, κ	leukocytes	157608	Biolegend
CD3	BV750	Rat IgG2b, κ	T-lymphocytes	100249	Biolegend
CD4	BV421	Rat IgG2b, κ	helper T cells	100443	Biolegend
CD8a	PerCP/Cy5.5	Mouse IgG1, κ	cytotoxic T cells	155014	Biolegend
CD19	PE-Cy7	Mouse IgG1, κ	mature B cells	159810	Biolegend
B220/CD45R	BV510	Rat IgG2b, κ	All stages B cells	563103	BD Biosciences
F4/80	BV711	Rat IgG2b, κ	macrophages	123147	Biolegend
CD11c	BV605	Armenian Hamster IgG	DC	117334	Biolegend
CD11b	APC	Rat IgG2b, κ	DC	101212	Biolegend
CD25	PE	Rat IgG2b, κ	Treg cells	101904	Biolegend
CD21/CD35	APC/Cy7	Rat IgG2b, κ	B1b cells	123418	Biolegend
CD5	BV650	Rat LOU/C, LOU/M IgG2a, κ	B1a cells	740444	BD Biosciences
PDCA-1 (tetherin)	PE-eFluor 610	Rat IgG2b, κ	pDC	61-3172-82	Thermo Fisher Scientific

References

1. Luo, Z., Solang, C., Mejia-Cordova, M., Thorvaldson, L., Blixt, M., Sandler, S., and Singh, K. (2019). Kinetics of immune cell responses in the multiple low-dose streptozotocin mouse model of type 1 diabetes. FASEB Bioadv 1, 538-549. 10.1096/fba.2019-00031.

2. Tonne, J.M., Sakuma, T., Deeds, M.C., Munoz-Gomez, M., Barry, M.A., Kudva, Y.C., and Ikeda, Y. (2013). Global gene expression profiling of pancreatic islets in mice during streptozotocin-induced beta-cell damage and pancreatic Glp-1 gene therapy. Dis Model Mech 6, 1236-1245. 10.1242/dmm.012591.

3. Goyal, S.N., Reddy, N.M., Patil, K.R., Nakhate, K.T., Ojha, S., Patil, C.R., and Agrawal, Y.O. (2016). Challenges and issues with streptozotocin-induced diabetes - A clinically relevant animal model to understand the diabetes pathogenesis and evaluate therapeutics. Chem Biol Interact 244, 49-63. 10.1016/j.cbi.2015.11.032.

4. Patra, M., Klochendler, A., Condiotti, R., Kaffe, B., Elgavish, S., Drawshy, Z., Avrahami, D., Narita, M., Hofree, M., Drier, Y., et al. (2024). Senescence of human pancreatic beta cells enhances functional maturation through chromatin reorganization and promotes interferon responsiveness. Nucleic Acids Res 52, 6298-6316. 10.1093/nar/gkae313.

5. Kim, Y.T., and Steinberg, C. (1984). Immunologic studies on the induction of diabetes in experimental animals. Cellular basis for the induction of diabetes by streptozotocin. Diabetes 33, 771-777. 10.2337/diab.33.8.771.

6. Lukic, M.L., Stosic-Grujicic, S., and Shahin, A. (1998). Effector mechanisms in low-dose streptozotocin-induced diabetes. Dev Immunol 6, 119-128. 10.1155/1998/92198.
